# Social dynamics of AI adoption in parents’ educational decisions

**DOI:** 10.1073/pnas.2533193123

**Published:** 2026-07-31

**Authors:** Leonardo Bursztyn, Alex Imas, Rafael Jiménez-Durán, Aaron Leonard, Christopher Roth

**Affiliations:** ^a^ Department of Economics, University of Chicago, Chicago, IL 60637; ^b^ Booth School of Business, University of Chicago, Chicago, IL 60637; ^c^ Department of Economics, Bocconi University, Milan 20136, Italy; ^d^ Department of Economics, University of Cologne, Cologne 50923, Germany

**Keywords:** AI, education, social pressure, technology adoption, rat race

## Abstract

When adopting a performance-enhancing technology offers short-run relative advantages, competitive pressure can drive diffusion even if most adopters would prefer a collective ban. We study this dynamic in the context of unrestricted AI tools for teenagers’ education. In preregistered, incentive-compatible experiments with approximately 2,000 parents in the United States, Canada, and the United Kingdom, we elicit willingness to pay for a three-month premium subscription to a leading general-purpose AI platform. Demand increases steeply with the share of other teenagers using AI: Moving perceived peer take-up from 20% to 80% raises willingness to pay by more than 60%. We then test whether information about emerging evidence on potential downstream skill effects changes behavior. Risk information substantially shifts incentivized beliefs about longer-run impacts but has little effect on individual demand, yet it strongly increases support for school-level restrictions. Follow-up experiments show that peer-adoption information does not significantly shift perceived AI quality, the importance parents assign to core skills, or support for bans, pointing to social pressure rather than social learning as the primary channel. The results reveal a rat-race dynamic in which individual adoption responds to peers even as collective preferences shift toward restriction.

## Significance

Our study shows that competitive social pressures, rather than cost–benefit analyses, can increase the diffusion of new technologies before their long-term costs are fully understood. We study this in the context of the unrestricted use of new AI tools for teenagers in education. Using experiments, we show that parental demand for these tools rises steeply as the share of other teenagers using the technology increases. At the same time, information about the potential long-term risks shifts beliefs but has little effect on short-term demand, yet increases support for school-level restrictions. Our work suggests collective policy interventions may be required to overcome these social pressures.

The fear of falling behind can produce a rat race in the adoption of performance-enhancing technologies: As use spreads, nonadopters face a growing relative disadvantage, making individual adoption privately attractive even when many people would prefer a collective ban. We study this dynamic in the context of general-purpose AI tools in education. The evidence on AI’s longer-run impacts on learning is still emerging and actively debated: While AI can improve short-run performance on some tasks (1–3), some studies point to negative effects on downstream skills once the tool is removed (4–6). This uncertainty is precisely what makes competitive dynamics dangerous since adoption may accelerate before long-run effects are well understood.

We study these dynamics among parents of teenagers, who are key gatekeepers for adolescent technology use. We ask: when parents decide whether to provide unrestricted AI tools (i.e. without guardrails) for schoolwork, do their choices reflect beliefs about long-run human capital, or are they primarily shaped by anxiety about losing relative standing in the short run? To answer this question, our paper investigates rat race dynamics in the adoption of AI tools through incentivized, preregistered experiments involving approximately 2,000 parents of teenagers in the United States, Canada, and the United Kingdom.

To measure parental demand for advanced AI tools, we elicit parents’ willingness to pay (WTP) for a three-month premium subscription to an unrestricted leading generative AI model for their teenagers’ use in schoolwork. Drawing on previous work on social externalities (7), we estimate how peer adoption rates causally influence parental demand. Parents report their WTP under scenarios where varying proportions (20% to 80%) of other teenagers in their state already use AI tools.^*^ Increasing proportions of peers’ usage capture the social pressure for their teenagers to avoid falling behind peers (8–13). To test whether information about AI’s risks can counteract social pressure, parents are also randomly assigned to either a control group, receiving information emphasizing AI’s short-term educational benefits, or a treatment group that additionally highlights potential longer-term risks to quantitative reasoning associated with unrestricted reliance on AI systems in education. Notably, this “AI-risk” information is taken from an existing study that documents the risk of unrestricted use, i.e., AI without guardrails (4).^†^

We find that parents’ WTP increases by more than 60% as peer take-up rises from 20% to 80%. Further, the risk information treatment leads to a large negative shift (−0.43 SDs) in incentivized beliefs about the effects of AI on cognitive skills, but does little to curb demand: Parents remain largely responsive to teenage peer adoption. Consistent with rat race dynamics, this same information increases parents’ support for banning AI in education. Open-ended responses confirm that a substantial portion of parents justify allowing their child to use AI due to fears of falling behind, despite supporting a ban.

These patterns are distinct from social learning, where higher peer adoption would signal product quality and reduce support for restrictions. Preregistered mechanism experiments confirm this: Peer-adoption information does not shift perceived AI quality, support for bans, nor the importance parents assign to core cognitive skills—ruling out the most commonly invoked channel in models of technology diffusion (14). Instead, the perceived importance of quantitative reasoning and critical thinking remains stable even as AI adoption increases, consistent with parents knowingly trading off long-term human capital concerns against the short-run costs of falling behind.

To probe the external validity of our findings, we conducted two additional preregistered experiments. The rat race dynamics are robust to substantially stronger risk information: The stronger treatment shifts the level of demand, but the peer-adoption gradient remains positive and significant. The dynamics also generalize beyond unrestricted general-purpose AI to education-specific tools—structured AI tutors and math solvers—though collective ban preferences differ across tool types. To mitigate possible demand effects, we also conduct two between-subject replications of our main experiment, where a respondent is randomized to only one scenario (20% or 80%). We find that the magnitudes for both the peer-adoption gradient and the effect of the erosion information on demand are qualitatively similar to the within-subjects experiments, suggesting these results are not driven by the within-subjects elicitation.

Our findings build on a large literature on peer influence in educational decisions (14–17). Taken together, our results demonstrate that social pressure can sustain adoption of a technology that parents would collectively prefer to restrict—and that this dynamic is resistant to correction through information provision alone. Achieving socially optimal outcomes may require collective action, such as school-level adoption of structured AI tools or coordinated opt-out agreements.

## 1. Framework: The AI Rat Race

We develop a framework that models parents’ demand for AI as a function of its short-run and long-run impact on their children. The formal model can be found in *SI Appendix*, section 2. We present the intuition here. Parents care both about their child’s relative academic performance in the short run and about their long-run human capital. When other teenagers adopt unrestricted AI tools, the nonadopter’s child suffers a short-run relative performance decline—a negative externality. As a result, even parents who believe that unrestricted AI use erodes long-run cognitive skills may still choose to adopt it for their child when other children use it provided that the relative short-run gains are large enough. Further, as more teenagers use the tool, the worse the nonadopter’s child’s relative position becomes, increasing their demand for the tool.

This resulting strategic environment resembles a prisoner’s dilemma: Although parents collectively prefer that no child uses unrestricted AI at school, each parent individually is incentivized to allow use when others do. Behavior tracks descriptive norms—what peers do—while parental support for restrictions captures injunctive norms—perceptions of what ought to be done (18, 19). Our framework predicts that beliefs about long-run risks shape collective preferences: Parents who believe AI harms long-run human capital are more likely to favor school-level bans. However, as long as short-run competition dominates parental concerns, individual demand for AI remains unchanged—even when beliefs about long-run outcomes become more pessimistic.

In our empirical analyses, we test two predictions from this model:

1. Demand for advanced AI tools increases in response to peer take-up.
2. Information about long-run risks increases support for collective bans, without changing individual demand.

## 2. Data and Design

### 2.1. Sample: Parents as AI Gatekeepers.

To test our hypotheses, we conduct experiments with parents of teenagers aged 13 to 18—the key gatekeepers of adolescent technology use.^‡^ Their beliefs and preferences shape both household adoption of AI tools and broader norms around educational technology. The sample was recruited via Prolific, an online research platform, with eligibility limited to parents with children meeting the age criteria. In every experiment, we sampled residents of the United States. We also include residents of the United Kingdom and Canada in Experiments 2, 3, 5, 6, 7, and 8. As prespecified, we only include respondents who correctly complete a comprehension check that is explained in more detail below.

The final sample of our two main experiments consists of 1,992 parents with at least one teenage child aged 13 to 18. The average age of respondents is 48.2 y (SD = 8.8), with a roughly balanced gender distribution: 53% female and 47% male. Respondents report an average of 1.34 children in the 13 to 18 age range. Self-reports indicate that 92.3% of parents and 92.7% of teenagers have been exposed to AI tools, suggesting near-universal knowledge about AI across both groups (see *SI Appendix* for a detailed breakdown).

### 2.2. Experimental Design.

#### 2.2.1. Overview.

Parents report demographic characteristics, beliefs about AI adoption among peers, and their own and their teenager’s AI usage. We then measure how willingness to pay (WTP) for unrestricted use of an advanced AI tool varies in response to i) randomized information about both AI’s potential educational benefits and long-term risks, and ii) varying levels of peer adoption.

#### 2.2.2. Background characteristics.

At the outset, we collect demographic information including the respondent’s year of birth, gender, race/ethnicity, and the number and age of their children. We restrict the analysis to parents with at least one child aged 13 to 18. Respondents report both their own and their teenager’s exposure to AI tools (e.g., ChatGPT, Claude, Gemini, Llama), as well as their beliefs about AI adoption rates among other teenagers.

#### 2.2.3. Pretreatment beliefs about the effects of using AI tools.

We measure respondents’ beliefs about the impact of unrestricted AI use on cognitive skills and human capital using an incentivized question: Participants estimate the treatment effect from a real study.^§^ Our participants receive the following instructions:

A field experiment with high school students tested how access to AI (GPT) for learning affected their quantitative reasoning skills. Students who had access to GPT throughout the semester later took a test on quantitative reasoning without AI.
By what percentage do you think their quantitative reasoning scores increased or decreased compared to students who never used GPT?

To incentivize this elicitation, parents are truthfully told that they will receive a monetary bonus if their estimate is close to the correct answer. We keep the instructions about incentives deliberately simple to avoid participant confusion (21).

#### 2.2.4. Information treatment.

To assess how information about the effects of unrestricted AI on quantitative skills influences beliefs and valuations, we randomly assign participants to one of two information conditions. In the control group, respondents receive information about recent studies that found improvements in writing and language performance when students use AI tools during learning. In particular, respondents are told:

Recent research has shown that using advanced AI models helps improve performance on writing and language tasks while it is being used.

In the treatment group, participants receive the same message, followed by an additional sentence reporting the results of a field experiment in which high school students with semester-long access to unrestricted AI experienced a nearly 20% decline in quantitative reasoning scores when tested without the tool.^¶^ In particular, respondents are additionally told:

On the other hand, a field experiment with high school students found that access to GPT for learning led to a nearly 20% decline in quantitative reasoning scores when students were later tested without AI.

To enhance credibility, parents are informed that declining test scores may reflect students’ overreliance on AI tools like GPT, resulting in reduced independent problem-solving, diminished mental effort, and decreased cognitive engagement.

#### 2.2.5. Willingness to pay elicitation.

To measure parents’ valuation of AI access for their children, we implement an incentive-compatible mechanism to elicit willingness to pay (WTP) for a three-month premium AI subscription, worth $60 in total. The incentive compatibility of the mechanism mitigates concerns about social desirability bias (23). Specifically, parents are asked for their maximum willingness to pay for a premium unrestricted AI subscription for their child. If their stated willingness to pay exceeds a randomly drawn bonus, they receive the subscription; otherwise, they receive the money. This mechanism ensures that it is optimal for participants to truthfully report their valuation (24). To ensure high data quality, parents need to pass a comprehension question on how the mechanism works.

We focus on a premium plan for a leading AI model because it is a real commercial product with a nontrivial price tag, creating meaningful trade-offs in parental valuation.^#^

#### 2.2.6. Social scenarios.

We assess how parental demand varies with AI adoption of their children’s peers. Parents are presented with a series of scenarios in which different proportions of teenagers (20%, 40%, 60%, or 80%) in their state use AI tools. For each scenario, parents report how much they would be willing to pay to obtain the premium AI subscription. We use a within-subjects design to maximize statistical power and to enable natural within-respondent comparisons across scenarios. Existing evidence suggests that demand effects are of similar magnitude in within- and between-subjects designs (25), and our incentive-compatible BDM elicitation further limits social-desirability concerns (23). *SI Appendix*, Fig. S3 reports a between-subjects replication confirming that the peer-adoption gradient is robust to this approach. To ensure incentive compatibility, parents are truthfully told that we will use the empirically closest scenario to their actual peer environment to determine which of the choices is implemented. *SI Appendix*, Fig. S1 shows that respondents attach a high likelihood to each of these scenarios occurring, indicating that they deem them as consequential. While one may be concerned about belief updating, we conduct a related version of this experiment, elaborated on in Section 4.2 to examine whether social learning contributes to our observed effects. Social learning in this context would refer to parents updating on the quality of AI based on the social scenarios revealing information on the private valuations of others.

#### 2.2.7. Posttreatment beliefs about the effects of using AI tools.

To quantify the extent to which information moves beliefs about the effects of AI on cognitive skills, we elicit incentivized posttreatment beliefs similarly to pretreatment beliefs, but using a different underlying study by ref. 26. In particular, parents see the following instructions:

A different field experiment with college undergraduates tested how access to AI (GPT) for learning affected their critical thinking skills. Students who had access to GPT throughout the semester later took a test on critical thinking without AI.
By what percentage do you think their critical thinking scores increased or decreased compared to students who never used GPT?

We now turn from our experimental design to the patterns these measures reveal.

## 3. Results

### 3.1. Individual Demand Rises Steeply with Peer Adoption.

**Fig. 1.**
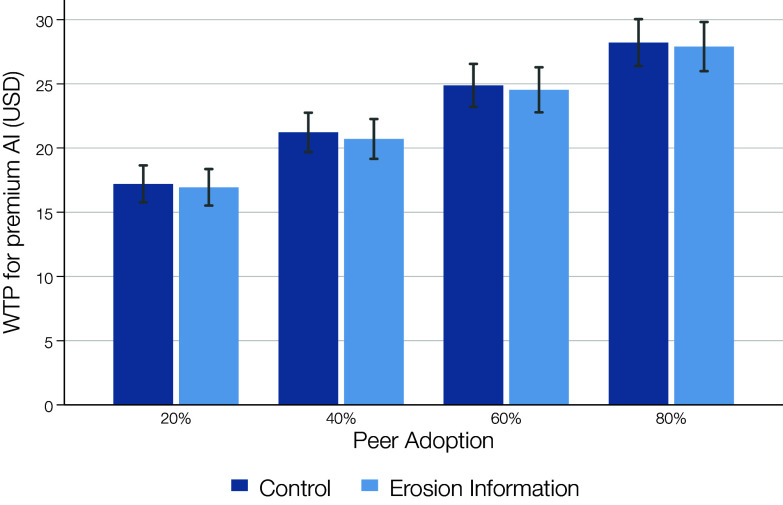
WTP by information treatment and peer adoption. *Notes:* This figure displays the mean willingness to pay for a three-month subscription for premium AI (worth $60), conditional on peer adoption of AI tools (the percentage of other students who use AI) and the information treatment. The analysis uses data from Experiment 1, as defined in *Materials and Methods*, consisting of 993 respondents. Bars in dark blue represent the control condition (n=511); bars in light blue represent the erosion information condition (n=482). Error bars indicate 95% CIs for the group means.

We begin by examining how social context shapes parents’ willingness to pay for AI tools. Fig. 1 shows that parents are highly responsive to social context. When asked to state their WTP for premium AI under different scenarios of peer AI adoption (20%, 40%, 60%, or 80%), average WTP increases as take-up rises. In a pooled linear regression across scenarios, we estimate that WTP rises by $1.83 for a 10 percentage point increase in peer usage. When peer take-up increases from 20% to 80%, WTP increases by more than 60%. This effect is both economically large and highly statistically significant (P<0.001). *SI Appendix*, Table S1 describes these results in regression format. Further, respondents viewed each of these social scenarios as consequential, as shown in *SI Appendix*, Fig. S1.^‖^

### 3.2. AI-Risk Information Leaves Individual Demand Unchanged but Increases Support for Collective Restrictions.

**Fig. 2.**
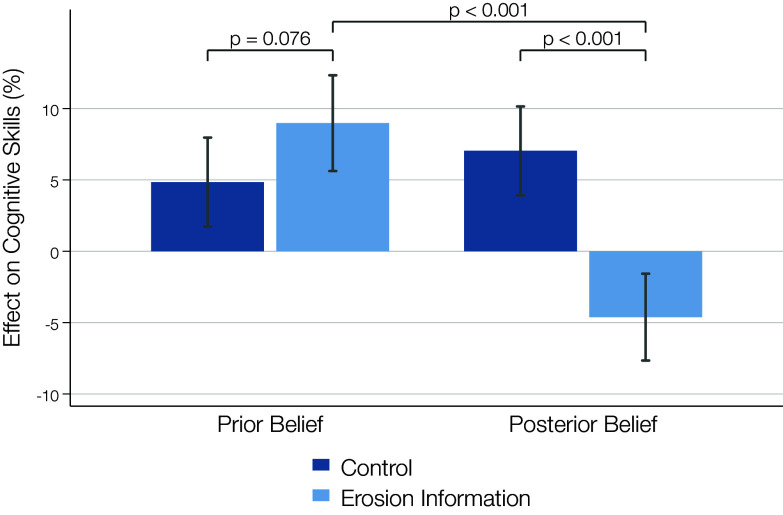
Average prior and posterior beliefs. *Notes:* This figure shows and compares the group means of respondents’ prior and posterior beliefs, and reports *P* values from two-sided *t* tests. The analysis uses data from Experiment 1, as defined in *Materials and Methods*, consisting of 993 respondents. Bars in dark blue represent the control condition (n=511); bars in light blue represent the erosion information condition (n=482). Error bars indicate 95% CIs.

We next turn to the effects of information about AI-risk. The treatment successfully shifts beliefs about the impact of AI on quantitative reasoning skills. Fig. 2 shows that, before treatment, both treatment and control group respondents believe AI has positive effects on quantitative skills. Posttreatment, control group respondents retain this belief, while treatment group respondents, on average, believe that using unrestricted AI tools decreases quantitative skills in the long run. These differences are economically sizable (0.43 SDs) and represent a qualitative shift in perceptions about the long-term risk of AI use in education (P<0.001, two-sided *t* test). *SI Appendix*, Fig. S2 displays the distribution of beliefs.

Despite the qualitative change in beliefs, the risk information has virtually no effect on demand for AI tools (P=0.747). Moreover, the slope of WTP with respect to peer take-up remains unchanged across treatment arms: We find no evidence that risk information reduces susceptibility to peer adoption.

**Fig. 3.**
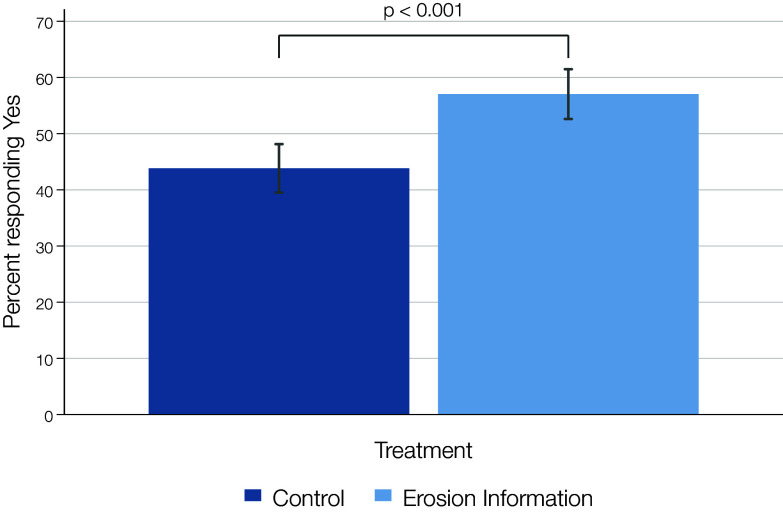
Preference for all students learning without AI. *Notes:* This figure displays the percentage of respondents who answer Yes to the following question: “Would you prefer if no student was allowed to use AI tools and instead learned the way they used to before AI tools were introduced?” The analysis uses data from Experiment 1, as defined in *Materials and Methods*, consisting of 993 respondents. Bars in dark blue represent the control condition (n=511); bars in light blue represent the erosion information condition (n=482). The *P* value is from a two-sided *t* test. Error bars indicate 95% CIs for the group means.

Yet the same information sharply increases support for collective restriction. We ask participants whether they would prefer to live in a world where no students were allowed to use AI tools. Fig. 3 shows that a substantial share expresses such a preference—desiring a return to a pre-AI educational era. Strikingly, 57% of parents in the erosion information group (treatment) prefer a world where no students use AI, compared to 44% of parents in the control group. This difference is sizable and statistically significant (P<0.001) and underscores the conflict between individual and social preferences.

### 3.3. Boundary Conditions and Generalizability.

Two preregistered follow-up experiments examine whether i) stronger risk information can overcome the peer-adoption gradient, and ii) the pattern generalizes across AI tool types.

In Experiment 5, we intensified the treatment beyond the single-study framing used in the main experiment by adding a summary of a policy report synthesizing a broad body of work and warning that, on the current trajectory, the risks of AI in education may outweigh the benefits. The stronger treatment shifts beliefs and—unlike in Experiment 1—also reduces the average WTP, indicating that information can move the level of demand on the margin when presented with stronger evidence. Crucially, however, WTP continues to increase strongly with perceived peer adoption even for those who receive the stronger AI risk information.^**^

We next ask whether the peer-pressure gradient generalizes across AI tools. Our main experiments measure demand for a premium, unrestricted general-purpose AI model, Claude Pro. To assess external validity across product types, we conducted a preregistered experiment (Experiment 6, N=202) in which parents evaluated two education-oriented tools that differ in how directly they substitute for independent problem-solving: a structured tutoring system (Khanmigo, $44/y) versus an answer-engine style math solver (Photomath, $70/y). Across both products, willingness to pay rises sharply with peer take-up, and the magnitudes of the peer-adoption gradients are similar. This pattern suggests that the social-pressure channel is not specific to one model or to a particular pricing tier, but reflects broader competitive anxiety about AI adoption in education. At the same time, parents’ collective preferences differ markedly across tool types: 56% of parents support banning AI math solvers compared with only 39% for the structured tutoring system (P=0.013). The combination of i) similar peer-adoption slopes in individual demand and ii) differing normative preferences across product design reinforces the policy implication that tool architecture and guardrails can shape collective acceptability even when social pressure continues to operate at the individual level. That said, both products are marketed in Anglophone countries and evaluated by parents on an online platform, and the broader landscape of AI in education—such as AI-powered grading systems and tools embedded directly in school curricula—remains untested. Whether the same patterns hold across these product categories, in other educational systems, or among populations with lower digital engagement remains an open question.

### 3.4. Between-Subjects Replication.

To mitigate the concern that the within-subjects experiment could create experimenter demand or consistency bias, we ran a between-subjects version (N=186; *SI Appendix*, Fig. S3). Respondents were assigned either the 20% or 80% adoption scenario. Following best practices in the literature (27, 28), we illustrate this information using an infographic. We then elicited WTP for a three-month premium AI subscription using the same BDM mechanism as the main experiment. Mean WTP is $17.98 in the 20% arm and $26.02 in the 80% arm, an $8.04 difference (P<0.01). The magnitude is qualitatively similar to the 20% to 80% difference in the within-subjects experiment (≈$11), indicating that the peer-adoption gradient is not an artifact of the within-subjects elicitation.

We additionally run a 2 by 2 between-subjects replication of our main experiment (N=410; *SI Appendix*, Fig. S4). Respondents are randomized to either the control or erosion information treatment and then cross-randomized to either the 20% or 80% adoption scenario, using the same elicitation as the previous between-subjects experiment. We find that the peer adoption gradient is statistically significant (P<0.01), and that the gradient does not significantly differ between the information treatment conditions (P=0.78). Moreover, in the between-subjects replication, AI-risk information also leaves individual demand virtually unchanged with point estimates close to zero (P=0.69), suggesting this result is not driven by the within-subjects elicitation (*SI Appendix*, Table S2).

## 4. Mechanisms

### 4.1. Rat Race Dynamics.

The data reveal a tension between parents’ collective preferences and their individual choices: Many allow their children to use AI while also believing that it would be better if nobody used AI in the educational context. Three pieces of evidence support rat-race dynamics as an important driver of these results.

First, we ask parents why they support a ban of AI tools at school even though they allow their child to use them.^††^ A systematic content analysis of these responses reveals that approximately 20% of parents cite concerns about nonusers falling behind, the most frequent category of responses (*SI Appendix*, Fig. S5). Given measurement error in open-ended responses, we view these estimates as lower bounds for the actual importance of these motives. For a detailed description of our coding scheme, see *SI Appendix*, *Methods*. Qualitative comments such as “I let my teenager use AI because I don’t want them to fall behind their peers” and “We let him use AI so he can keep up with his peers,” reinforce the interpretation that competitive anxiety, not purely positive beliefs about AI, drives demand. This pattern aligns with our experimental finding that WTP rises with peer adoption and confirms prior work on how perceived social externalities and competitive pressures shape parents’ educational choices (29–31).

Second, a large fraction of parents explicitly agree with key aspects of the rat race interpretation when asked. *SI Appendix*, Fig. S6 shows that a majority of parents (77.6%) agree with the statement that “I think AI tools will help my child now, but I’m worried about the long-term impact using AI tools will have on my child.” Notably, agreement is above 70% even for the control group, which was not exposed to AI-risk information. In experiment 3 (*SI Appendix*, Fig. S7), we find additional support, including that a majority of parents believe not using AI puts their child at a disadvantage compared with their peers, and would not allow their child to use AI if their peers did not.

Third, we ran a second experiment that is nearly identical to the main design, but where information about the short-run benefits of AI use was not made salient. We use this experiment to establish the robustness of the first study. It replicates the finding that social information strongly shapes WTP for AI learning tools and also confirms that the effects of AI-risk information are relatively muted for demand, though somewhat larger in this experiment. This is consistent with the idea that the anxiety about falling behind is less top-of-mind when the short-run benefits are not mentioned across treatments (*SI Appendix*, Table S3).

Additionally, this second experiment allows us to compare parents’ susceptibility to social information when the risks and short-run benefits are either made salient or not. *SI Appendix*, Table S4 provides suggestive evidence that parents become more susceptible to information about peer take-up when the short-run information is made salient. This further supports the interpretation that anxiety about their child falling behind in the short-run plays an important role in driving the demand for AI tools.

### 4.2. Alternative Mechanism: Social Learning.

An alternative explanation for our results is social learning: Parents may positively revise their beliefs about the quality and benefits of AI tools based on others’ adoption. If this channel is important, higher peer adoption should also reduce support for bans. We test this prediction in two preregistered experiments. Experiment 3 elicits parents’ support for banning AI tools in schools under one of two randomized peer-adoption scenarios in the parent’s state (20%, or 80%). *SI Appendix*, Fig. S8 shows that support for the ban does not significantly differ across these two scenarios with a point estimate close to zero (P=0.747). If anything, the point estimate is positive—opposite to the social learning prediction. This null contrasts sharply with the large effects of AI-risk information on the preference to ban, underscoring that support for the ban is an elastic outcome to begin with.^‡‡^

Experiment 4 tests the same hypothesis with an information treatment design that is more well-powered and that mitigates concerns about the hypothetical nature of Experiment 3. In this experiment, we provide an information treatment on the percentage of teenagers that use AI in their state based on a sample of responses from previous surveys we conducted. We truthfully randomize this percentage to either 40% or 80%, as discussed in *SI Appendix*, *Methods*. This approach credibly conveys information on the private valuations of others, which should shift quality perceptions if social learning is an important channel. In *SI Appendix*, Fig. S9, we show that the information treatment meaningfully shifts participants’ posterior beliefs on teenage AI take-up in their state, validating the first stage from our treatment. However, as shown in *SI Appendix*, Fig. S10, we find no statistically significant difference in either the preference to ban AI or the perceived quality of the AI product.^§§^ Taken together, these results suggest that social learning about AI quality is not the primary driver of the WTP increase documented in Experiments 1 and 2.

### 4.3. Alternative Mechanism: Future Skill Requirements.

Another candidate explanation is that higher peer uptake leads parents to prioritize AI literacy over quantitative reasoning and critical thinking for long-term human capital. Experiment 4 tests this by eliciting the perceived importance of various skills for the long-term development of their teenagers. The information treatment does not significantly affect the perceived importance of AI literacy (*SI Appendix*, Fig. S11) or other skills, including quantitative reasoning (*SI Appendix*, Fig. S12).^¶¶^ In fact, in terms of levels, critical thinking and quantitative reasoning are both viewed as highly valued skills by parents. These null results are inconsistent with a mechanism whereby WTP increases because parents update on the importance of AI skills relative to cognitive skills for long-term human capital. Instead, our results support the rat-race interpretation: Parents prioritize short-term competitive standing over long-term human capital concerns.

## 5. Discussion

Our findings reveal a rat race in AI adoption for education: Parents increase demand in response to peer take-up even as many would prefer collective restraint. This pattern echoes the classic distinction between what people do and what they believe should be done (18, 19), and connects to a long-standing literature on positional externalities and status competition (32–36).

The competitive anxiety we document is consistent with sociological evidence that parental educational decisions are shaped less by private cost–benefit analysis than by perceived competitive stakes and peer networks (15, 16, 37, 38), with peer influence documented across course-taking (17), school transitions (39), and school choice (40). Our contribution is to show that these social forces operate with sufficient strength to sustain adoption of a technology that parents themselves view with ambivalence—and to quantify the disconnect between private behavior and collective preference using incentive-compatible methods.

Our mechanism tests rule out social learning—the most commonly invoked channel in models of technology diffusion (14)—as a driver of these results. Peer-adoption information does not shift perceived AI quality, support for bans, nor the importance parents assign to cognitive skills. This distinction matters for policy: If adoption were driven by learning about product quality, better information would be sufficient to correct inefficient diffusion. The rat-race channel implies that information alone cannot resolve the problem, because each parent’s optimal choice depends on what others do.

Our findings also carry implications for AI tool design. We find that the peer-adoption gradient extends across product types, but parents’ collective preferences differ markedly: Support for bans is substantially lower for structured tutoring tools than for answer-providing tools that substitute for independent problem-solving. Tool architecture can thus shift the normative equilibrium even when competitive pressure persists, suggesting that structured AI tutors may offer a path out of the rat race. At the same time, our experiments are conducted with parents in Anglophone countries with broadly similar educational institutions; the strength of rat-race dynamics may differ in settings with different competitive structures, grading norms, or levels of AI penetration.

More broadly, the mechanisms we document mirror the competitive dynamics currently unfolding in the global race to deploy AI systems: firms, countries, and institutions face pressure to adopt quickly for fear of falling behind, even as they voice concern about long-term risks. For firms, rat race dynamics can generate overinvestment in AI technology: Conditional on other firms investing, the fear of falling behind induces investment even when individual returns are not profitable. This can generate a potential bubble in the industry.^##^ Understanding how rat race dynamics interact with technological change is essential not only for educational policy but for managing broader technological transitions that pose complex trade-offs between short-run gains and long-run consequences.

## 6. Materials and Methods

The human subjects research described in this article was approved by the University of Chicago Social and Behavioral Sciences Institutional Review Board (IRB25-0487). Informed consent was obtained from all participants. Recruitment for all eight experiments took place on Prolific. In *SI Appendix*, *Methods*, we outline each of the seven experiments, including fielding dates, sample size, details, and statistical tests.

## Supplementary Material

Appendix 01 (PDF)

## Data Availability

Anonymized survey data, survey instructions, and data analysis code have been deposited in OSF (41).

## Footnotes

^*^We implement the peer uptake scenario based on the actual rate of AI adoption that we find from our survey, ensuring the WTP is incentivized for every social scenario.

^†^To further test the rat race hypothesis, we also analyze treatments where parents are made aware of the potential long-run costs of adoption, but not of the short-run benefits of AI adoption and find similar results.

^‡^For example, ChatGPT requires users to be at least 13 y of age, and parental consent is required for users 13 to 18 y old (20).

^§^For the pretreatment elicitation of beliefs, we use a field experiment conducted with nearly 1,000 Turkish high school students (4). The authors found a 17% reduction in math test scores for students who used unrestricted AI during the study, after access to the tool was removed.

^¶^The eventual productivity effects of AI are still highly uncertain and subject to considerable academic debate (22). Our analysis is focused on the potential for rat-race dynamics conditional on beliefs about productivity effects.

^#^The premium plan increases usage by a substantial factor over the free plan, which is what the vast majority of participants have access to.

^‖^The peer-adoption gradient does not differ significantly across the three countries in our sample, suggesting that the rat-race dynamic operates similarly across the Anglophone settings we study.

^**^To speak toward external validity, we added a survey question measuring the perceived competitiveness of respondents’ local educational environment. We find modestly larger peer-gradient effects among parents who perceive their educational environment as more competitive, though the differences are not statistically significant—consistent with the idea that rat-race dynamics may be stronger in more competitive settings, while leaving the precise magnitude for future work.

^††^The exact wording of the question was: “You indicated that you would prefer if students were not allowed to use AI. You also indicated that you let your teenager use AI at least sometimes. Why is that?”

^‡‡^The muted difference in support for a ban across the social scenarios provides suggestive evidence that our main results are not driven by experimenter demand effects. Specifically, it suggests that the substantial differences in WTP documented across social scenarios are unlikely to result from participants responding to perceived expectations of the experimenters (25).

^§§^If social learning was the main mechanism for our results, we would expect that people would update on the quality of the AI product, explaining the WTP increase. We find a small positive coefficient that is not statistically significant.

^¶¶^We only find a significant effect on critical thinking, but in the opposite direction of a future skill mechanism since we find that critical thinking skills—which unrestricted AI use may erode—are even more valued in the high peer adoption condition.

^##^The prospect of an AI bubble is an active topic of discussion; see, for example, https://www.wired.com/story/ai-bubble-will-burst.
