# Supplementary material for: Social dynamics of AI adoption in parents’ educational decisions
Source: Proc Natl Acad Sci U S A. 2026 Jul 31;123(31):e2533193123. doi: 10.1073/pnas.2533193123 (PMC13439080; doi:10.1073/pnas.2533193123)
Supplement: Supplementary file 1 [file pnas.2533193123.sapp.pdf]

# Supporting Information for

## Social Dynamics of AI Adoption in Parents' Educational Decisions

**Leonardo Bursztyn, Alex Imas, Rafael Jiménez-Durán, Aaron Leonard and Christopher Roth**

**Corresponding Author: Leonardo Bursztyn.**
**E-mail: bursztyn@uchicago.edu**

### This PDF file includes:

Supporting text
Figs. S1 to S12
Tables S1 to S14
SI References

## Supporting Information Text

## 1. Methods

The human subjects research described in this article was approved by the University of Chicago Social and Behavioral Sciences Institutional Review Board (IRB25-0487). The methods were carried out in accordance with relevant guidelines and regulations. Informed consent was obtained from all participants.

**A. Debriefing.** Because the study provides participants with summaries of emerging research, all participants in the relevant experiments received a debrief message via Prolific after completion. The debrief explained the study's purpose, clarified that the informational statements summarized findings from specific recent studies in an evolving evidence base (not scientific consensus), and noted that effects can differ across AI tools and usage designs (e.g., structured tutoring systems versus unrestricted general-purpose tools).

**B. Samples.** Recruitment for all eight experiments took place on Prolific. Experiments 1 and 2 were fielded between March 31 and April 8, 2025, with a target sample size of n=1,000 each. The actual sample sizes are 993 and 999, respectively. Experiment 3 was fielded on June 3-4, 2025, with a target and actual sample size of n=200. Experiment 4 was fielded between October 24-28, 2025. We collected 500 responses as well as 174 pilot responses that we include to increase statistical power. Experiment 5 was fielded on February 6-12, 2026 with a target sample of 400 and actual sample of 401. Experiment 6 was fielded on February 6, 2026 with a target sample of 200 and actual sample of 202. Experiment 7 was fielded on April 18, 2026 with a sample of 186. Experiment 8 was fielded on June 12, 2026, with a sample of 410. Screeners were used to limit the sample to adults with children. Custom in-survey screening was used to additionally limit the sample to parents of at least one teenager (ages 13-18) and to people who correctly answered comprehension checks, when applicable. Our main experiment and Experiment 4 were fielded with U.S. residents only, while Experiments 2, 3, 5, 6, 7, and 8 additionally include residents of the United Kingdom and Canada. For detailed sample descriptives including demographics, see Supplementary Information.

**C. Pre-registration.** We pre-registered six data collections. The pre-registrations include the experimental design, hypotheses, analysis, sample sizes, and exclusion criteria.

- Experiment 1 (main): https://aspredicted.org/cggm-p9yp.pdf
- Experiment 2: https://aspredicted.org/4x57-6r46.pdf
- Experiment 3: https://aspredicted.org/qmb6-djsw.pdf
- Experiment 4: https://aspredicted.org/si5xq8.pdf
- Experiment 5: https://aspredicted.org/fv6vj2.pdf
- Experiment 6: https://aspredicted.org/7pu8z7.pdf

**D. Details of Experiments 1 and 2.** Experiments 1 and 2 are nearly identical to each other. An initial demographics battery is used to screen out respondents who do not have children in the target age group. Next are questions about the respondent's own self-reported use of AI tools, as well as their teenage child's, measured on a five-item Likert scale from "Never" to "Very frequently".

This is followed by the first of two nearly identical belief elicitations. Respondents are presented with a brief description of a field experiment which tested how AI-supported learning affected the test scores of students. To allow independent elicitation of pre-treatment and post-treatment beliefs, two distinct field experiments are referenced: (1, 2). After reading the brief description, respondents are asked to estimate the percentage by which AI use increased or decreased test scores relative to a control group, by moving a slider from -100 to 100. To incentivize higher effort, respondents are truthfully told that they will receive a bonus payment if they come close to the correct answer. Respondents who came within $\pm 2$ percentage points of the correct answer received a bonus payment of $1.

The next part of the survey is composed of an incentive-compatible mechanism designed to elicit parents' willingness-to-pay (WTP) for AI tools for their children. Respondents are informed that one in every 100 participants will be randomly selected to receive either the premium AI subscription (valued at $60) or a monetary bonus. They are told that the subscription is intended for their teenage child's use, e.g. to help with homework, grammar, or research (although we have no way of enforcing this).

Whether they receive the premium AI subscription or a bonus depends on their stated willingness to pay. This is elicited as follows. Each selected participant's outcome is determined via a Becker–DeGroot–Marschak (BDM) procedure (3): if their stated WTP exceeds a randomly drawn bonus, they receive the subscription; otherwise, they receive the money. This mechanism ensures that it is optimal for participants to truthfully report their valuation. To ensure high data quality, respondents need to correctly answer a simple comprehension question.[*] As pre-registered, only respondents that pass this comprehension question (49.9%) are allowed to proceed with the experiment.

[*] The question was: "Which of the following statements is **true** about how the premium AI subscription or bonus will be allocated? (i) Entering a higher valuation for the premium AI subscription makes it **less likely** that I will receive the subscription and more likely that I will receive the bonus. (ii) The amount I am willing to forgo for the premium AI subscription **does not affect** whether I receive the subscription or the bonus. (iii) Entering a higher valuation for the premium AI subscription makes it **more likely** that I will receive the subscription and less likely that I will receive the bonus."

To allow elicitation of WTP conditional on AI adoption among other teenagers, willingness to pay is elicited in four different scenarios. Specifically, all respondents are given four answer fields to state their WTP, corresponding to four hypothetical scenarios in which the share of other teenagers using AI is 20%, 40%, 60% or 80%. They are told that their prize will be determined by their stated WTP in the scenario that comes closest to the actual share determined by our survey.[†] [‡]

The difference between Experiments 1 and 2 lies in the information conditions (see Table S5), which are shown on the same screen as the answer fields for WTP. While the provision of erosion information is randomized between treatment and control in both experiments, Experiment 1 additionally shows information about potential short-run benefits of AI use to both treatment and control groups (active control). Experiment 2 shows no such additional information.

As stating a WTP higher than \$60 is functionally equivalent to stating a WTP of \$60 (besides being arguably nonsensical), we top-code all values above \$60. This is in accordance with our pre-registrations for both experiments.

The remainder of the survey does not differ between the two experiments. Post-treatment beliefs about the cognitive effects of AI use are elicited as described above, again incentivized with a bonus payment. This is followed by a yes-or-no question whether the respondent would prefer students to be banned from using AI. If the answer is yes, *and* if the respondent self-reported earlier that their child uses AI tools at all (i.e., "Never" was not selected), they are prompted to explain the implied contradiction in an open-ended text box. A direct yes-or-no question asking essentially whether the respondent agrees with our hypothesized short-run vs. long-run trade-off is placed at the very end, as it would otherwise be likely to induce experimenter demand effects.

**E. Details of Experiment 3.** The purpose of Experiment 3 is to rule out social learning as an alternative mechanism driving the positive relationship between WTP for AI and peer adoption.

Experiment 3 starts with a demographics battery to screen out respondents who do not have teenage children. Then all participants receive short-run information and erosion information about the effects of AI use, identical to what the treatment group sees in Experiment 1. Participants are then told about a prospective survey which will estimate the share of teenagers in their country who use AI tools, and are informed that the next question will be hypothetical, based on possible outcomes of that survey.

At this point respondents are randomly assigned into one of two groups: high and low adoption. The high-adoption group receives the prompt

> Imagine the scenario that **80% of teenagers** use AI tools.

while for the low-adoption group, the prompt says "20% of teenagers" instead. Displayed below this prompt is a yes-or-no question whether the respondent would prefer students to be banned from using AI, with the same wording as the other experiments:

> Would you prefer if no student was allowed to use AI tools and instead learned the way they used to before AI tools were introduced?

In this experiment, support for an AI ban is the only outcome of interest. The experiment does not attempt to repeat the willingness-to-pay or belief elicitations from the previous two. We included three Likert-style questions, which were not pre-registered, for exploratory purposes at the end of the survey.

**F. Details of Experiment 4.** The purpose of Experiment 4 was to further rule out social learning using an alternative experiment that additionally collected information on perceived quality of AI products and the importance of long-term skills.

Experiment 4 also starts with a demographics battery to screen out respondents who do not have teenage children. Then all participants receive short-run information and erosion information about the effects of AI use, identical to what the treatment group sees in Experiment 1.

Next, we randomize participants into one of two information treatments about the take-up of AI use among teenagers in their state.[§] The high adoption information treatment receives:

> We recently ran a survey on AI use. Among a sample of these respondents in your state, we found that approximately 80% of teenagers use AI tools.

while the low adoption information treatment says 40% instead of 80%. We then ask the same ban preference question as in Experiment 3. We additionally ask respondents to rate their perceived quality of Claude Pro on a Likert scale from 1 to 10, the AI product used in Experiment 1 and 2.

We also ask a set of questions about parents' perceived importance of long-term skills in the future. In particular, for critical thinking, writing and communication, creativity, quantitative reasoning, teamwork, and self-discipline, we ask:

> How important do you think the following skills will be to the success of your teenager over the next 10 years?

on a Likert scale from Not at all important to Extremely important. We also ask on a scale from 1 to 10:

[†] We use IP addresses to determine the region of parents, and we define adoption as the share of respondents who indicate that their child uses AI at least "rarely". Several regions appear only once or twice in our data. Still, in practice, all regions we observe at all have empirical adoption rates above 70%, except South Dakota.

[‡] Since the company behind the AI model currently offers no way of gifting subscriptions or vouchers, participants who won the subscription ultimately received a payment of \$60 (the cost of the subscription), a link to the AI model's website where they can create a premium account, and instructions to cancel their subscription on time if they do not wish to spend money in excess of their prize.

[§] We only recruit participants for states in which the information treatment is true based on the responses from our previous survey. We then tell all respondents the share of teenage use of AI across the entire US to ensure all respondents receive information on the full distribution of our survey sample.

> How important do you think AI literacy is to the long term success of your teenager?

Finally, we include the same final three Likert questions as in Experiment 3. We collected an additional 174 responses as part of a pilot. We opt to pool our data with the pilot responses in order to increase our statistical power. Our primary outcome variables remain not significant at conventional levels without the pilot responses.

**G. Details of Experiment 5.** The purpose of Experiment 5 is to test whether a stronger information treatment can overcome the social pressure dynamics documented in Experiments 1 and 2. The design closely follows Experiment 1, with the key difference being a stronger information treatment.

As in Experiment 1, participants complete a demographics battery, report their own and their teenager's AI use, and provide pre-treatment beliefs about the effects of AI on cognitive skills. They then receive one of two information conditions before eliciting WTP for a three-month Claude Pro subscription ($60 retail price) under four peer take-up scenarios (20%, 40%, 60%, 80%), using the same BDM procedure as in prior experiments.

In the control condition, respondents learn about the benefits of using AI tools on writing and language tasks while using them, identical to the control condition in Experiment 1. In the treatment condition, respondents receive the same benefit information, plus two additional elements: (1) the erosion information from Experiment 1 (a field experiment showing a nearly 20% decline in quantitative reasoning scores), and (2) information about a report warning that the risks of AI use in education outweigh the benefits. This treatment was designed to provide stronger and more socially salient evidence of harm than the single-study framing in Experiments 1 and 2.

Post-treatment beliefs and ban preferences are elicited as in Experiment 1. The target sample size was 400 parents of teenagers aged 13–18, recruited via Prolific. As in prior experiments, we restrict analysis to respondents who pass all comprehension checks and top-code WTP at $60.

**H. Details of Experiment 6.** The purpose of Experiment 6 is to test whether the peer-adoption gradient generalizes across different types of AI tools in education.

Participants complete a demographics battery and are randomized with equal probability into one of two product conditions: Khanmigo, a structured AI tutoring system (one-year subscription, $44 retail price), or Photomath, an AI-powered math solver (one-year subscription, $70 retail price). Participants receive a brief description of the assigned product and its educational features. Khanmigo provides scaffolded learning support, guiding students through problems step-by-step, while Photomath provides solutions to math problems with worked-out steps.

WTP is elicited using the same BDM procedure as in prior experiments under four peer take-up scenarios (20%, 40%, 60%, 80%). Participants also answer a product-specific ban question: whether they would prefer if no student was allowed to use the assigned type of AI tool—AI tutoring for the Khanmigo condition, AI math solvers for the Photomath condition—and instead learned the way they used to before such tools were introduced.

The target sample size was 200 parents of teenagers aged 13–18, recruited via Prolific. We restrict analysis to respondents who complete the survey and pass all comprehension checks. WTP is top-coded at the retail price of the assigned product ($44 for Khanmigo, $70 for Photomath).

**I. Details of Experiment 7.** The purpose of Experiment 7 is to test whether the results of our initial experiment hold in a between-subjects design. We follow the same procedure as Experiment 1 but we remove any information treatment as the primary goal is to test WTP with the between-subject design. Participants are assigned either the 20% or the 80% peer adoption scenario and state their WTP for the premium AI subscription. We represent the information in a visual info-graphic as this is commonly used to convey social information in between designs (4) and more generally viewed as a best practice in information experiments (5, 6).

The target sample was parents of teenagers aged 13–18, recruited via Prolific. We restrict analysis to respondents who complete the survey and pass all comprehension checks. WTP is top-coded at the retail price of the assigned product ($60 for Claude).

**J. Details of Experiment 8.** The purpose of Experiment 8 is to also add the information treatment to Experiment 7 to test whether the information treatment results also hold in a between-subjects design.

The target sample was parents of teenagers aged 13–18, recruited via Prolific. We restrict analysis to respondents who complete the survey and pass all comprehension checks. WTP is top-coded at the retail price of the assigned product ($60 for Claude). Due to a coding error, we only collect prior and posterior beliefs for part of the sample.

**K. Coding scheme for open-ended responses.** Experiments 1 and 2 include an open-ended question to provide further insight into the reasons for adopting AI tools in spite of potential reservations. The question is shown to respondents who indicate that their children use AI at least rarely, *and* who also indicate that they would "prefer if no student was allowed to use AI tools and instead learned the way they used to before AI tools were introduced." The text of the question is

> You indicated that you would prefer if students were not allowed to use AI. You also indicated that you let your teenager use AI at least sometimes. Why is that?

We do not impose a minimum or maximum length on the open-text box. Most responses are two sentences or less. We find almost no instances of responses appearing to be LLM-generated.

**Rationale** The goal of this analysis is to find further evidence for a rat race dynamic: a situation in which consumers find it individually optimal to use AI because of social pressure, but would prefer if nobody used it. A person may demand AI while simultaneously wanting to ban it for everyone if the negative utility of social externalities given non-adoption outweigh the negative utility of adoption.

Specifically, teenagers compete with their peers in school for good grades. In the "steroids" scenario, AI may be harmful in the long run but boosts performance while it is being used. Thus, there is a pressure to use AI to keep up with AI-using peers in the short run: given that many others already use AI to boost their performance, an individual child is better off if they also use AI, despite potential future risks. The framework in the next section formally outlines these predictions.

A parent outlining this type of scenario is classified as citing "social pressure" as a reason for allowing their child to use AI despite preferring a ban. We aim to quantify the prevalence of such reasoning relative to other types of responses in our sample.

**Hand-coding of responses** We defined seven categories, which are described in the Supplementary Information for reasons of space. We assigned each response to the category that fits best. Categories are not mutually exclusive, but responses are only assigned multiple categories if they fit equally well.

## L. Statistical tests.

**Peer adoption and WTP** This analysis is based on Experiment 1. We compare respondents' willingness to pay for premium AI across four different scenarios of peer AI adoption. Formally, we use ordinary-least-squares (OLS) to estimate the following linear regression model:

$$WTP_{is} = \alpha + \beta X_s + \varepsilon_{is}, \qquad [1]$$

where $WTP_{is}$ is respondent $i$'s willingness to pay for premium AI in scenario $s$, and $X_s$ is the share (in percent) of peers using AI in scenario $s$. No additional covariates are included. Table S1 shows that the results are robust to the inclusion of the treatment indicator and that the slope with respect to peer adoption does not vary across treatment conditions.

The sample consists of 993 respondents. For each respondent we obtain four measurements of WTP, one for each scenario, for a total of 3,972 observations. We use clustered standard errors at the individual level to account for the non-independence of observations of the same respondent. No other corrections or assumption tests were performed.

The estimated coefficient is $\beta = 0.183$ ($SE = 0.009$), 95% CI [0.167, 0.200]. A two-sided t-test with cluster-robust standard errors, using 992 degrees of freedom, yields a test statistic of $t = 21.44$ ($p < 0.001$). Figure 1 visualizes this result (disaggregated across treatment arms).

**Information and beliefs** This analysis is based on Experiment 1. We use a basic difference-in-differences framework to estimate the effect of erosion information on respondents' beliefs. Formally, we use OLS to estimate the following linear regression model:

$$B_{it} = \alpha + \beta Treat_i + \gamma Post_t + \delta Treat_i \times Post_t + \varepsilon_{it}, \qquad [2]$$

where $B_{it}$ is respondent $i$'s belief at time $t$ (pre- or post-treatment) about the effect (in percent) of AI use on students' test scores, $Treat_i$ indicates assignment to treatment or control group, and $Post_t$ indicates the timing of the observation. No additional covariates are included. We are interested in the coefficient $\delta$ on the interaction term.

The sample consists of 993 respondents. The control group consists of 511 respondents and the treatment group consists of 482 respondents. For each respondent we measure beliefs twice, once before treatment and once after, for a total of 1,986 observations. We use clustered standard errors at the individual level to account for the non-independence of observations of the same respondent. No other corrections or assumption tests were performed.

The estimated coefficient of interest is $\delta = -15.775$ ($SE = 2.170$), 95% CI $[-20.033, -11.517]$.[¶] A two-sided t-test with cluster-robust standard errors, using 992 degrees of freedom, yields a test statistic of $t = -7.27$ ($p < 0.001$). Figure 2 visualizes this result.

**Information and WTP** This analysis is based on Experiment 1. We compare respondents' willingness to pay for premium AI across the two treatment conditions (pooled across peer adoption scenarios). Formally, we use OLS to estimate the following linear regression model:

$$WTP_{is} = \alpha + \beta Treat_i + \varepsilon_{is}, \qquad [3]$$

where $WTP_{is}$ is respondent $i$'s willingness to pay for premium AI in scenario $s$, and $Treat_i$ indicates assignment to treatment or control group. No additional covariates are included. Table S1 presents alternate specifications which control for differences in level and slope across scenarios, with near-identical results.

The sample consists of 993 respondents. The control group consists of 511 respondents and the treatment group consists of 482 respondents. For each respondent we obtain four measurements of WTP, one for each scenario, for a total of 3,972 observations. We use clustered standard errors at the individual level to account for the non-independence of observations of the same respondent. No other corrections or assumption tests were performed.

The estimated coefficient is $\beta = -0.359$ ($SE = 1.114$), 95% CI [-2.546, 1.828]. A two-sided t-test with cluster-robust standard errors, using 992 degrees of freedom, yields a test statistic of $t = -0.32$ ($p = 0.747$). Figure 1 visualizes this result (disaggregated across scenarios).

**Information and willingness to ban** This analysis is based on Experiment 1. We compare respondents' support for banning AI in education across the two treatment conditions. Formally, we use OLS to estimate the following linear regression model:

$$Y_i = \alpha + \beta Treat_i + \varepsilon_i, \qquad [4]$$

where $Y_i$ is a dummy that takes the value 1 if respondent $i$ responds Yes to our ban question. No additional covariates are included.

The sample consists of 993 respondents. The control group consists of 511 respondents and the treatment group consists of 482 respondents. Support for a ban is measured only once per respondent, so it is not necessary to cluster standard errors. No other corrections or assumption tests were performed.

The estimated coefficient is $\beta = 0.132$ ($SE = 0.032$), 95% CI [0.070, 0.194]. A two-sided t-test with 991 degrees of freedom yields a test statistic of $t = 4.20$ ($p < 0.001$). Figure 3 visualizes this result.

The p-value in Figure S6 is computed analogously. The estimated coefficient is $\beta = 0.043$ ($SE = 0.026$), 95% CI [-0.009, 0.095] and the t-statistic is $t = 1.64$ ($p = 0.101$).

[¶] In the main text of the paper, we report this coefficient in terms of standard deviations of the prior belief, where $SD = 36.696$.

**Social learning in Experiment 3** This analysis is based on Experiment 3. Recall that this experiment randomly assigns respondents to one of two hypothetical peer adoption scenarios (20% and 80%), holding information constant. We compare respondents' support for banning AI across the two treatment conditions (i.e., across scenarios). Formally, we use OLS to estimate the following linear regression model:

$$Y_i = \alpha + \beta S_i + \varepsilon_i, \quad [5]$$

where $Y_i$ indicates support for banning AI in education, and $S_i$ is a binary dummy indicating the peer adoption scenario to which respondent $i$ was assigned, where $S_i = 1$ corresponds to the 80% scenario.

The sample consists of 200 respondents. The 20% adoption group consists of 101 respondents and the 80% adoption group consists of 99 respondents. Support for a ban is measured only once per respondent, so it is not necessary to cluster standard errors. No other corrections or assumption tests were performed.

The estimated coefficient is $\beta = 0.022$ ($SE = 0.069$), 95% CI [-0.114, 0.159]. A two-sided t-test with 198 degrees of freedom yields a test statistic of $t = 0.32$ ($p = 0.747$). Figure S8 visualizes this result.

**Social learning and skills in Experiment 4** This analysis is based on Experiment 4. Recall that this experiment randomly assigns respondents to one of two information treatments (40% and 80%). We compare respondents' answers for several outcomes variables across the two treatment conditions (i.e., across scenarios). Formally, for each outcome variable, we use OLS to estimate the following linear regression model:

$$Y_i = \alpha + \beta S_i + \varepsilon_i, \quad [6]$$

where $Y_i$ indicates outcome variable, and $S_i$ is a binary dummy indicating the information treatment to which respondent $i$ was assigned, where $S_i = 1$ corresponds to the 80% scenario.

The sample consists of 673 respondents. The 40% adoption group consists of 335 respondents and the 80% adoption group consists of 338 respondents.

For the perceived quality of Claude Pro, the estimated coefficient is $\beta = 0.177$ ($SE = 0.187$), 95% CI [-0.189, 0.543]. A two-sided t-test with 671 degrees of freedom yields a test statistic of $t = 0.95$ ($p = 0.343$). Figure S10 visualizes this result.

For the support for the ban outcome, the estimated coefficient is $\beta = -0.027$ ($SE = 0.037$), 95% CI [-0.099, 0.046]. A two-sided t-test with 671 degrees of freedom yields a test statistic of $t = -0.72$ ($p = 0.471$). Figure S10 visualizes this result.

For the posterior beliefs, the estimated coefficient is $\beta = 14.91$ ($SE = 1.30$), 95% CI [12.360, 17.461]. A two-sided t-test with 671 degrees of freedom yields a test statistic of $t = 11.48$ ($p < 0.001$). Figure S9 visualizes this result.

**Strong information treatment in Experiment 5.** This analysis is based on Experiment 5. The specifications are identical to those used for Experiment 1. We estimate Equation (1) to obtain the peer-adoption gradient, Equation (2) to estimate the effect of the strong information treatment on beliefs, Equation (3) to compare WTP across treatment conditions, and Equation (4) to compare ban preferences. All WTP and belief regressions use clustered standard errors at the individual level.

The sample consists of 401 respondents (217 control, 184 treatment). The estimated effect of the strong information treatment on beliefs is $\hat{\delta} = -12.676$ (SE = 3.191), 95% CI $[-18.950, \ -6.403]$, $t = -3.97$ ($p < 0.001$). The estimated effect on pooled WTP is $\hat{\beta} = -3.989$ (SE = 1.768), 95% CI $[-7.465, \ -0.514]$, $t = -2.26$ ($p = 0.025$). The peer-adoption gradient is $\hat{\beta} = 0.141$ (SE = 0.014), $p < 0.001$. The effect on ban preferences is $\hat{\beta} = 0.172$ (SE = 0.049), $t = 3.53$ ($p < 0.001$).

**Peer adoption across AI tool types in Experiment 6.** This analysis is based on Experiment 6. We estimate Equation (1) separately for the Khanmigo ($n = 108$) and Photomath ($n = 94$) subsamples, and compare ban preferences using Equation (4) with a product indicator replacing the treatment indicator. For Khanmigo, $\hat{\beta} = 0.132$ (SE = 0.020), $p < 0.001$. For Photomath, $\hat{\beta} = 0.150$ (SE = 0.031), $p < 0.001$. The difference in slopes is not statistically significant ($p = 0.631$). The share supporting a ban is 56% for Photomath and 39% for Khanmigo ($p = 0.013$).

**Between-subject design in Experiment 7.** This analysis is based on Experiment 7. Recall that this experiment randomly assigns respondents to one of two peer adoption scenarios (20% or 80%). We compare respondents' WTP across the two treatment conditions (i.e., across scenarios). Formally, for WTP, we use OLS to estimate the following linear regression model:

$$WTP_i = \alpha + \beta S_i + \varepsilon_i, \quad [7]$$

where $WTP_i$ indicates WTP, and $S_i$ is a binary dummy indicating the treatment to which respondent $i$ was assigned, where $S_i = 1$ corresponds to the 80% scenario. The sample consists of 186 respondents. The 20% adoption group consists of 93 respondents and the 80% adoption group consists of 93 respondents. The estimated coefficient is $\beta = 8.043$ ($SE = 2.572$), 95% CI [2.969, 13.116]. A two-sided t-test with 184 degrees of freedom yields a test statistic of $t = 3.13$ ($p = 0.002$). Figure S3 visualizes this result.

**Between-subject design in Experiment 8.** This analysis is based on Experiment 8. Recall that this experiment randomly assigns respondents to one of two peer adoption scenarios (20% or 80%) and also one of two information treatments (same as Experiment 1). We compare respondents' WTP across the two peer adoption scenarios (pooled across information treatments). Formally, for WTP, we use OLS to estimate the following linear regression model:

$$WTP_i = \alpha + \beta S_i + \varepsilon_i, \quad [8]$$

where $WTP_i$ indicates WTP, and $S_i$ is a binary dummy indicating the scenario to which respondent $i$ was assigned, where $S_i = 1$ corresponds to the 80% scenario. Table S2 shows that the results are robust to the inclusion of the treatment indicator and that the slope with respect to peer adoption does not vary across treatment conditions.

The sample consists of 410 respondents. The 20% adoption group consists of 207 respondents and the 80% adoption group consists of 203 respondents. The estimated coefficient is $\beta = 5.961$ (SE = 1.835), 95% CI $[2.353, 9.568]$. A two-sided $t$-test with 408 degrees of freedom yields a test statistic of $t = 3.25$ ($p = 0.001$). Figure S4 visualizes this result.

We then compare respondents' willingness to pay for premium AI across the two information treatment conditions (pooled across peer adoption scenarios). Formally, we use OLS to estimate the following linear regression model:

$$WTP_i = \alpha + \beta Treat_i + \varepsilon_i, \qquad [9]$$

where $WTP_i$ is respondent $i$'s willingness to pay for premium AI, and $Treat_i$ indicates assignment to the information (erosion) treatment or control group. No additional covariates are included. Table S2 presents alternate specifications which control for differences in level and slope across scenarios, with near-identical results.

The sample consists of 410 respondents. The control group consists of 205 respondents and the information treatment group consists of 205 respondents. The estimated coefficient is $\beta = -0.568$ (SE = 1.858), 95% CI $[-4.221, 3.085]$. A two-sided $t$-test with 408 degrees of freedom yields a test statistic of $t = -0.31$ ($p = 0.760$). Figure S4 visualizes this result (disaggregated across scenarios).

## 2. Conceptual Framework

There is a continuum of parents, $\mathcal{I}$. Each parent $i \in \mathcal{I}$ can choose whether their kids use an AI tool or not, which we denote by the indicator variable $x_i \in \{0, 1\}$. Let $X$ denote the aggregate use of AI among students. Each parent has two sources of utility: a short-run component that depends on the kid's current academic performance and a long-run component that depends on their future human capital. This framework is intentionally stylized; its purpose is to illustrate the interaction between short-run social externalities and long-run potential risk in driving current demand. We allow both of these components to depend on others' AI consumption decisions, to capture important spillovers in this setting.

Concretely, $i$'s utility is:

$$v_i(x_i, X, \delta) = u_i^{sr}(x_i, X) + \beta u_i^{lr}(h_i - \delta x_i, H - \delta X),$$

where $u_i^{sr}$ is the short-run component and $u_i^{lr}$ is the long-run one. The term $h_i$ denotes individual $i$'s long-run human capital in the absence of AI use and $\delta$ represents any depreciation caused by AI use. Likewise, $H$ denotes the aggregate human capital of all students. Note that short-run utility depends on other's AI use, to capture, for example, that students who do not use AI might fall behind as the usage of others is higher. Similarly, long-run utility depends on the human capital of all students, to capture potential complementarities due to knowledge spillovers.

For convenience, define the short-run and long-run gains of using AI (relative to not using it) as $\Delta u_i^{sr}(X) = u_i^{sr}(1, X) - u_i^{sr}(0, X)$ and $\Delta u_i^{lr}(X, \delta) = u_i^{lr}(h_i - \delta, H - \delta X) - u_i^{lr}(h_i, H - \delta X)$, respectively. We make the following assumptions about these utilities:

1. Parents believe there is a short-run benefit of allowing their kids to use AI. That is, $u_i^{sr}$ is increasing in their kids' AI use, or there are short run gains from AI, $\Delta u_i^{sr}(X) > 0$.

2. In the short run, parents exhibit negative non-user externalities; for example, parents' believe that their kids might fall behind when they do not use AI and others do. That is, $\frac{\partial u_i^{sr}(0,X)}{\partial X} < 0$. Moreover, the negative externalities are worse among non-AI users than among AI users, $\frac{\partial u_i^{sr}(1,X)}{\partial X} > \frac{\partial u_i^{sr}(0,X)}{\partial X}$.

3. If AI use depreciates human capital ($\delta > 0$), there is a long-term cost of allowing their kids to use AI. That is, $u_i^{lr}$ is increasing in their kids' human capital, or there are long-run losses from AI $\Delta u_i^{lr}(X, \delta) < 0$.

4. There are human capital complementarities between individual and aggregate human capital, $\frac{\partial^2 u_i^{sr}}{\partial h_i \partial H} > 0$.

The parents' willingness to pay to give their kids access to AI is:

$$WTP_i(X, \delta) = v_i(1, X, \delta) - v_i(0, X, \delta) = \Delta u_i^{sr}(X) + \beta \Delta u_i^{lr}(X, \delta).$$

For a given market price of AI $p$ and an aggregate level of AI use $X$, parents choose to give their kids access to AI if $WTP_i(X, \delta) \geq p$.

We also introduce notation to denote the parents' willingness to ban AI, given by:

$$WTB_i(X, \delta, p) = v_i(0, 0, \delta) - v_i(1, X, \delta) + p.$$

**AI rat race** We say that parent $i$ is in an *AI rat race* when the parent is willing to pay to give AI to their child ($WTP_i(X, \delta) \geq p$) but would simultaneously prefer AI to not exist ($WTB_i(X, \delta, p) \geq 0$). Both inequalities can be rewritten as bounds on the short-run gains from AI: $LB_i \leq \Delta u_i^{sr}(X) \leq UB_i$, where:

$$LB_i = \underbrace{\beta |\Delta u_i^{lr}(X, \delta)|}_{\text{LR losses}} + p$$

$$UB_i = \underbrace{\beta |\Delta u_i^{lr}(X, \delta)|}_{\text{LR losses}} + p + \underbrace{[u_i^{sr}(0, 0) - u_i^{sr}(0, X)]}_{\text{FOMO avoidance}} + \underbrace{\beta \left[u_i^{lr}(h, H) - u_i^{lr}(h, H - \delta)\right]}_{\text{Human capital externalities}}$$

These expressions say that parent $i$ is in a rat race when the short-run gains from using AI compensate the long-term losses in human capital (and the price of AI). At the same time, the rat race requires these short run gains to be lower than the gains from banning AI, given by the sum of three positive terms: 1) the reduction in long-term losses in human capital, 2) the avoidance of short-run anxiety that their kid falls behind, and 3) the externalities from an increased aggregate human capital.

**Positive network effects** The parents' AI decisions exhibit positive network effects when their WTP is increasing in $X$. This occurs when: $\frac{\partial \Delta u_i^{sr}(X)}{\partial X} \geq -\beta \frac{\partial \Delta u_i^{lr}(X,\delta)}{\partial X}$. This expression is equivalent to the following inequality:

$$\frac{\partial u_i^{sr}(1, X)}{\partial X} - \frac{\partial u_i^{sr}(0, X)}{\partial X} \geq -\beta \delta \left| \frac{\partial u_i^{lr}(h_i - \delta, H - \delta X)}{\partial H} - \frac{\partial u_i^{lr}(h_i, H - \delta X)}{\partial H} \right|,$$

which holds when AI depreciates long-run human capital ($\delta > 0$) since the left-hand side is positive due to our assumption (2).

**Impact of information about long-run skill decay** Lastly, we are interested in understanding the impact of providing information about the long-run skill decay due to AI, which we interpret as an increase in $\delta$ (where $\delta$ is positive).

First, the impact on the parents' WTP for AI is:

$$\frac{\partial WTP_i(X,\delta)}{\partial \delta} = -\beta \frac{\partial u_i^{lr}(h_i-\delta, H-\delta X)}{\partial h} + \beta X \left| \frac{\partial u_i^{lr}(h_i-\delta, H-\delta X)}{\partial H} - \frac{\partial u_i^{lr}(h_i, H-\delta X)}{\partial H} \right|.$$

Thus, the effect has a sign of ambiguous effect since it is the sum of two components with opposite sign. The first component results from a higher perceived loss in the kids' human capital and the second component results from the fact that the damage from the aggregate loss in human capital is now lower due to the knowledge spillovers. On net, both effects can cancel out, as we observe empirically.

The impact on the parent's willingness to ban AI is:

$$\frac{\partial WTB_i(X,\delta)}{\partial \delta} = \beta \left[ \frac{\partial u_i^{lr}(h_i-\delta, H-\delta X)}{\partial h} + X \frac{\partial u_i^{lr}(h_i-\delta, H-\delta X)}{\partial H} \right].$$

This impact is unambiguously positive since a higher long-run skill decay decreases both the kids' human capital and aggregate human capital.

## 3. Supplementary Figures

**Fig. S1. Beliefs about likelihood of different adoption levels.**

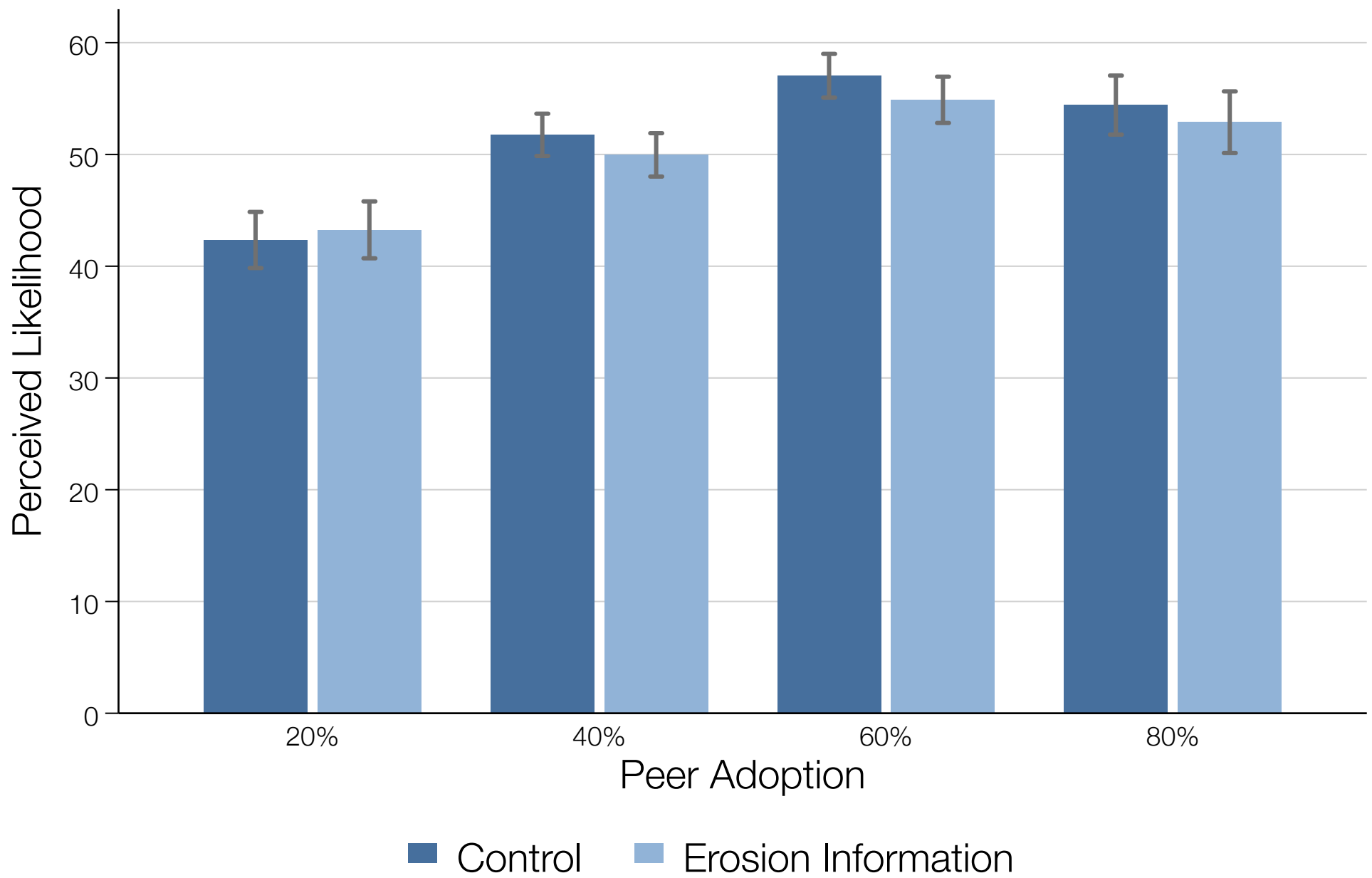


*Notes:* This figure shows the mean stated likelihood of each social scenario as perceived by respondents. These perceived likelihoods were elicited immediately following the willingness-to-pay elicitation. The analysis uses data from Experiment 1, as defined in Methods, consisting of 993 respondents. Bars in dark blue represent the control condition (n=511); bars in light blue represent the erosion information condition (n=482). Error bars indicate 95% confidence intervals for the group means.

**Fig. S2. Distribution of posterior beliefs.**

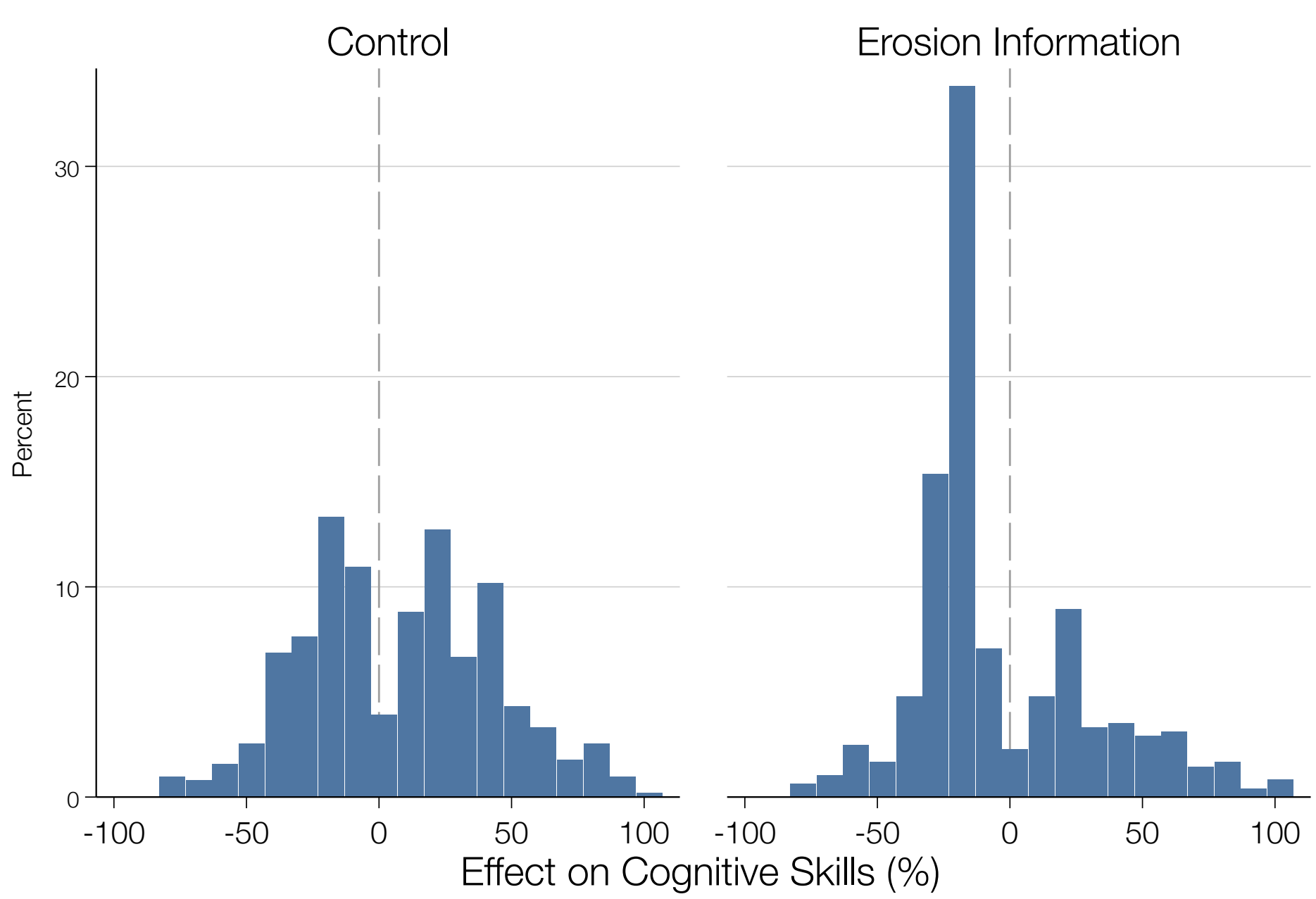


*Notes:* This figure shows the distribution of posterior beliefs by treatment group. The left panel shows the belief distribution for control group respondents, while the right panel shows the belief distribution for respondents in the erosion information group. The analysis uses data from Experiment 1, as defined in Methods, consisting of 993 respondents. The bins of each histogram are 10 percentage points wide. A vertical line is drawn at 0 as a visual reference.

**Fig. S3. Between-subjects replication of peer adoption scenarios.**

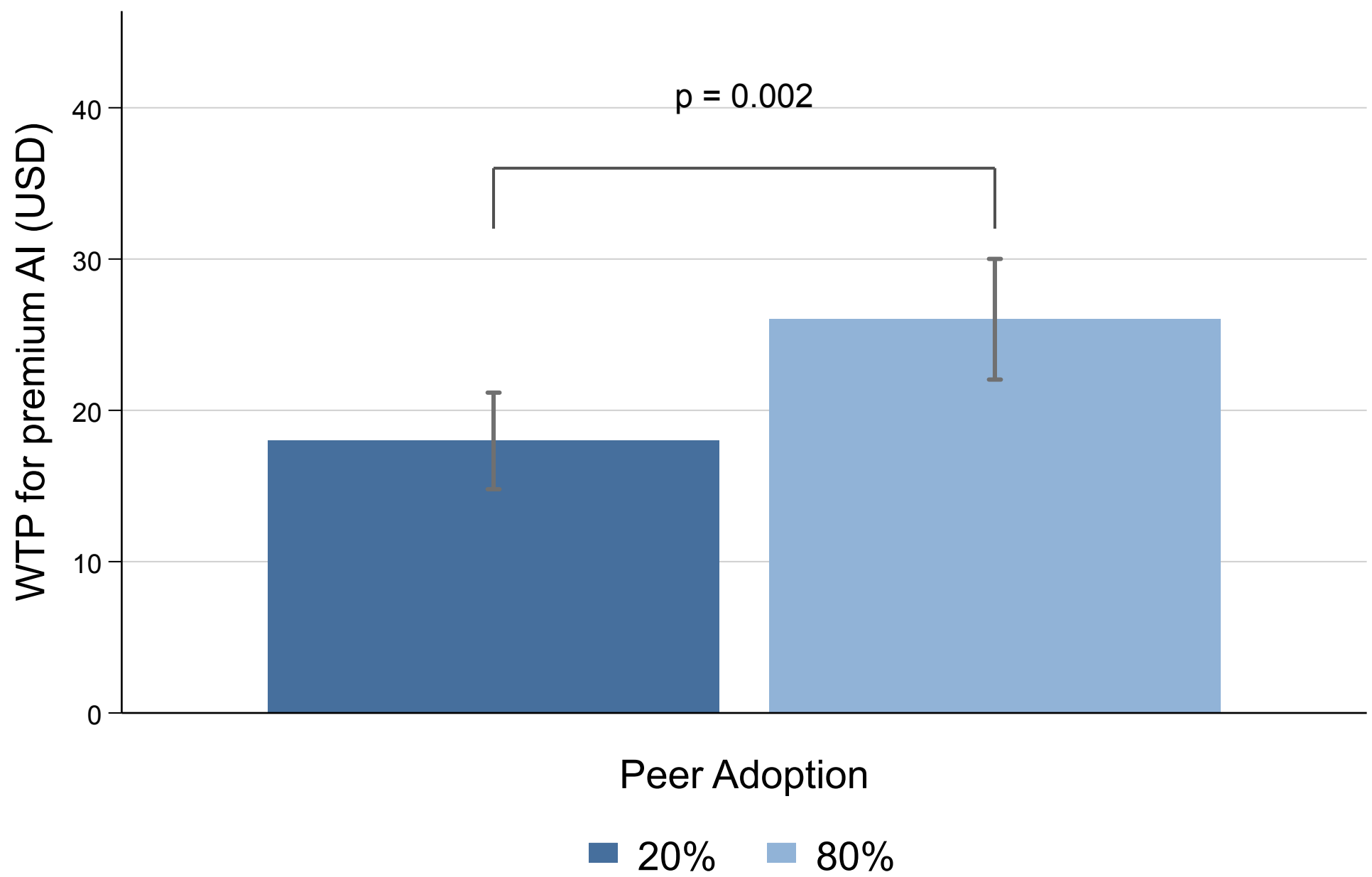


*Notes:* This figure displays the mean willingness to pay for a three-month subscription for premium AI (worth $60), conditional on one assigned peer adoption scenario of AI tools (the percentage of other students who use AI). The analysis uses data from Experiment 7, as defined in Methods, consisting of 186 respondents. The bar in dark blue represents the 20% condition (n=93); the bar in light blue represents the 80% condition (n=93). Error bars indicate 95% confidence intervals for the group means.

**Fig. S4. Between-subjects replication of full main experiment.**

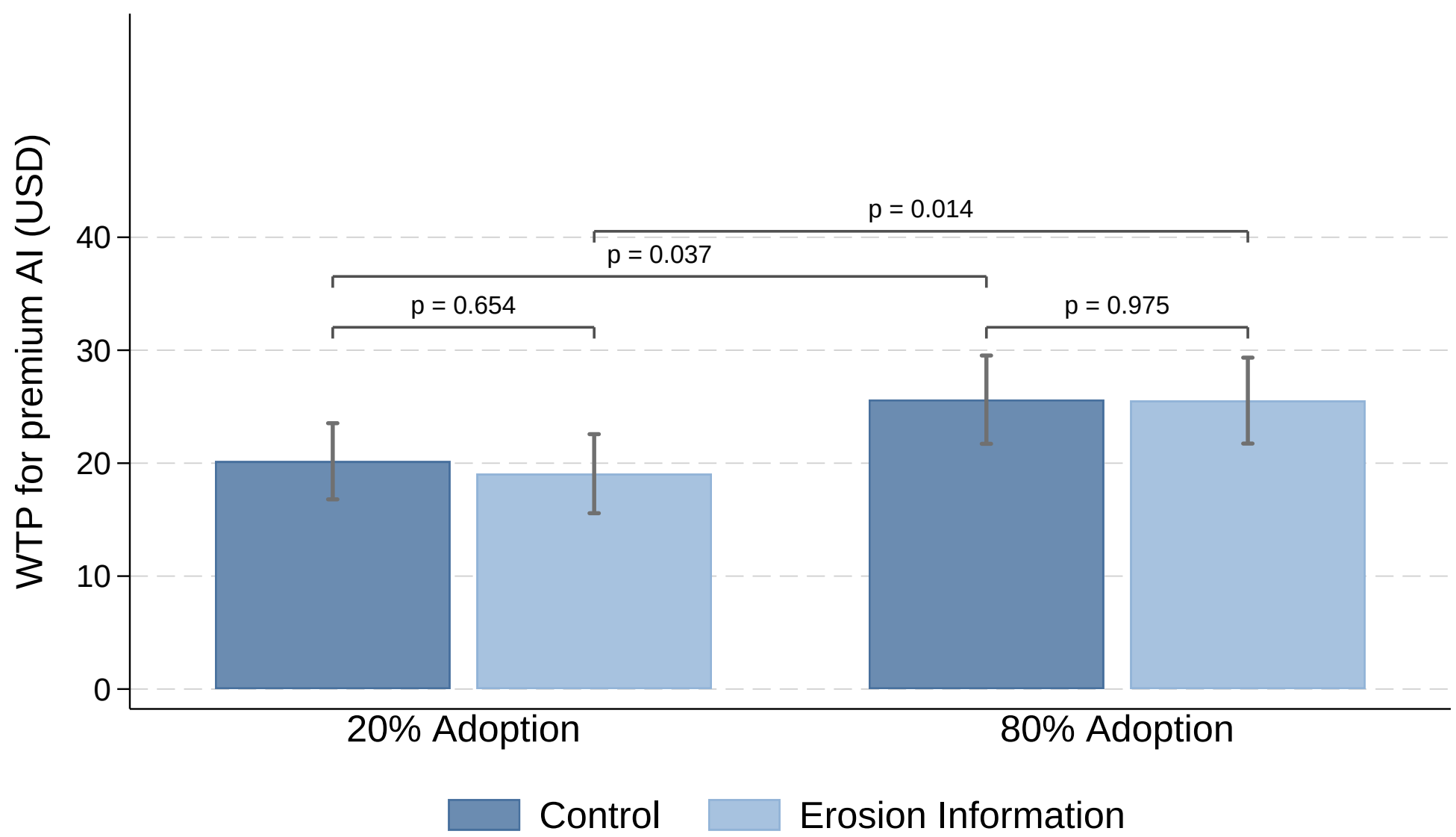


*Notes:* This figure displays the mean willingness to pay for a three-month subscription for premium AI (worth $60), conditional on one assigned peer adoption scenario of AI tools (the percentage of other students who use AI) and the information treatment assigned. The analysis uses data from Experiment 8, as defined in Methods, consisting of 410 respondents. Error bars indicate 95% confidence intervals for the group means.

**Fig. S5. Reasons behind preference for no student being allowed to use AI.**

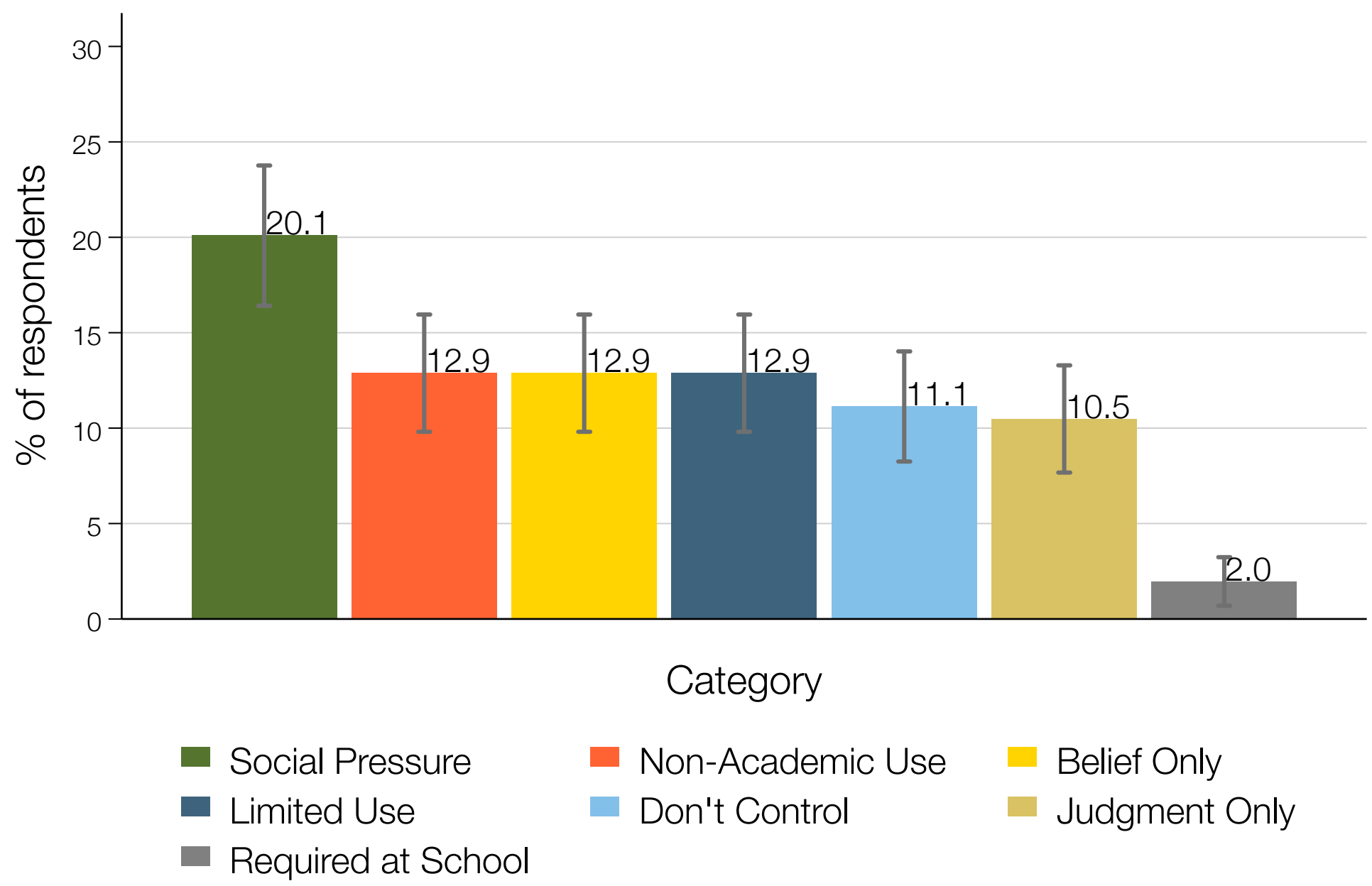


*Notes:* This figure shows the results of our open-ended analysis for the question "You indicated that you would prefer if students were not allowed to use AI. You also indicated that you let your teenager use AI at least sometimes. Why is that?". The analysis is based on the subset of respondents from Experiment 1 to whom this question was shown because of their previous answers (n=458). Responses were hand-coded by a research assistant. *Category definitions:* Social Pressure: allows use to avoid peer disadvantage; Non-Academic Use: allows only outside schoolwork; Belief Only: belief stated without justification; Limited Use: restricted or supervised access; Don't Control: no parental oversight; Judgment Only: normative view without rationale; Required at School: mandated by school.

**Fig. S6. Agreement with AI helps now, but worried about long-term impact.**

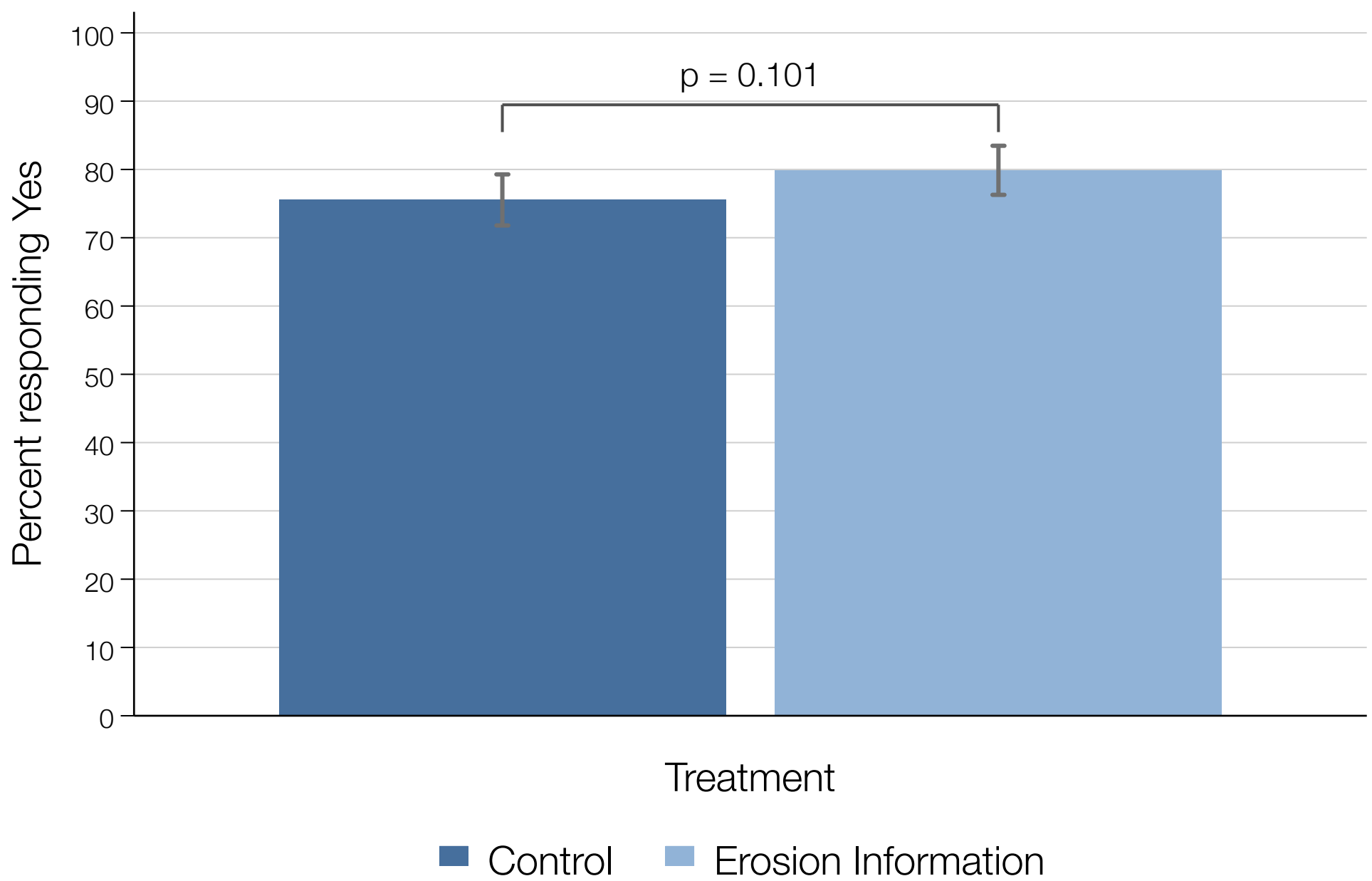


*Notes:* This figure displays the percentage of respondents who indicate agreeing with the following statement: "I think AI tools will help my child now, but I'm worried about the long-term impact using AI tools will have on my child." The analysis uses data from Experiment 1, as defined in Methods, consisting of 993 respondents. Bars in dark blue represent the control condition (n=511); bars in light blue represent the erosion information condition (n=482). The p-value is from a two-sided t-test. Error bars indicate 95% confidence intervals for the group means.

**Fig. S7. More evidence for mechanisms**.

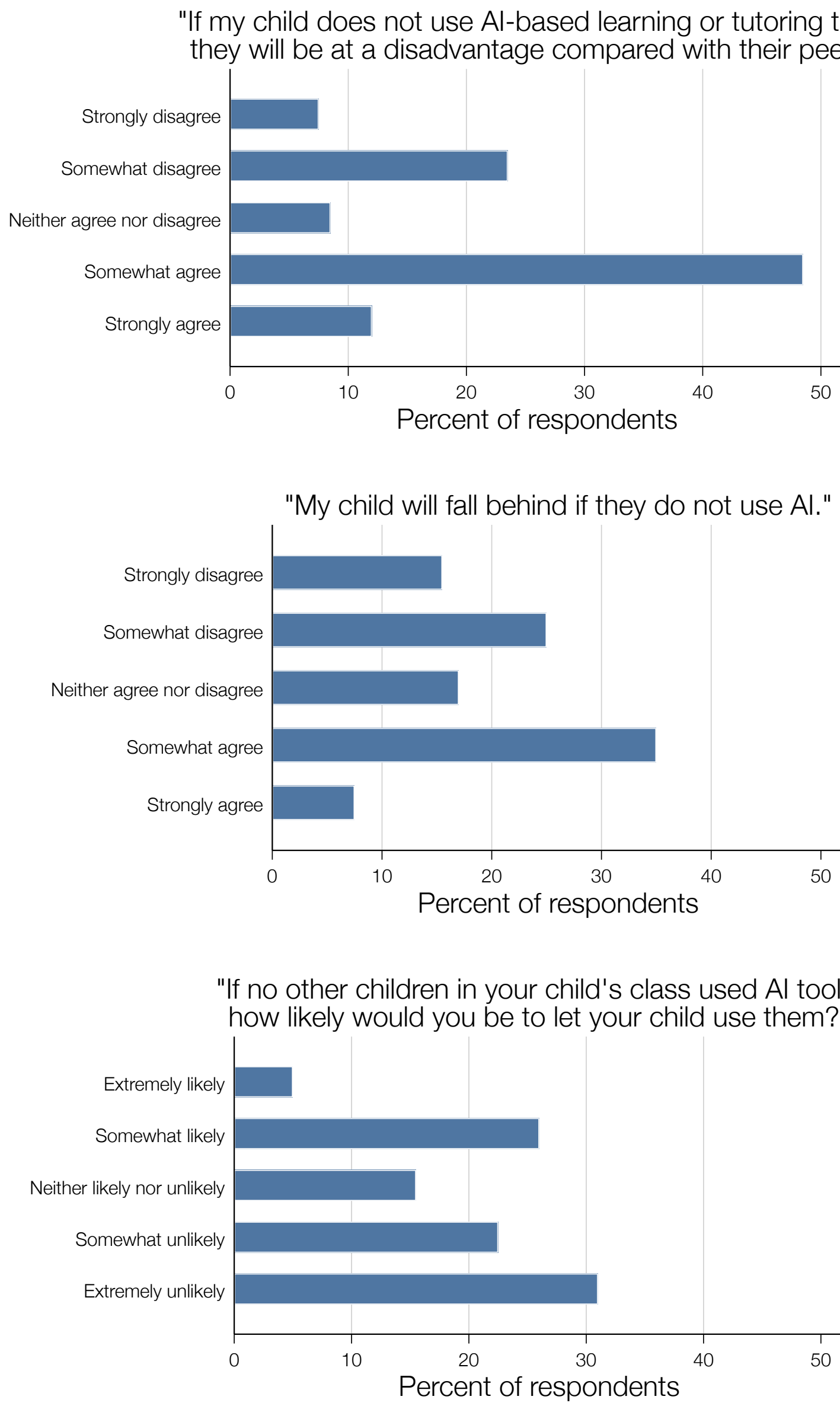


*Notes:* This figure displays results of three additional questions that were asked at the end of Experiment 3.

**Fig. S8. No evidence for social learning**.

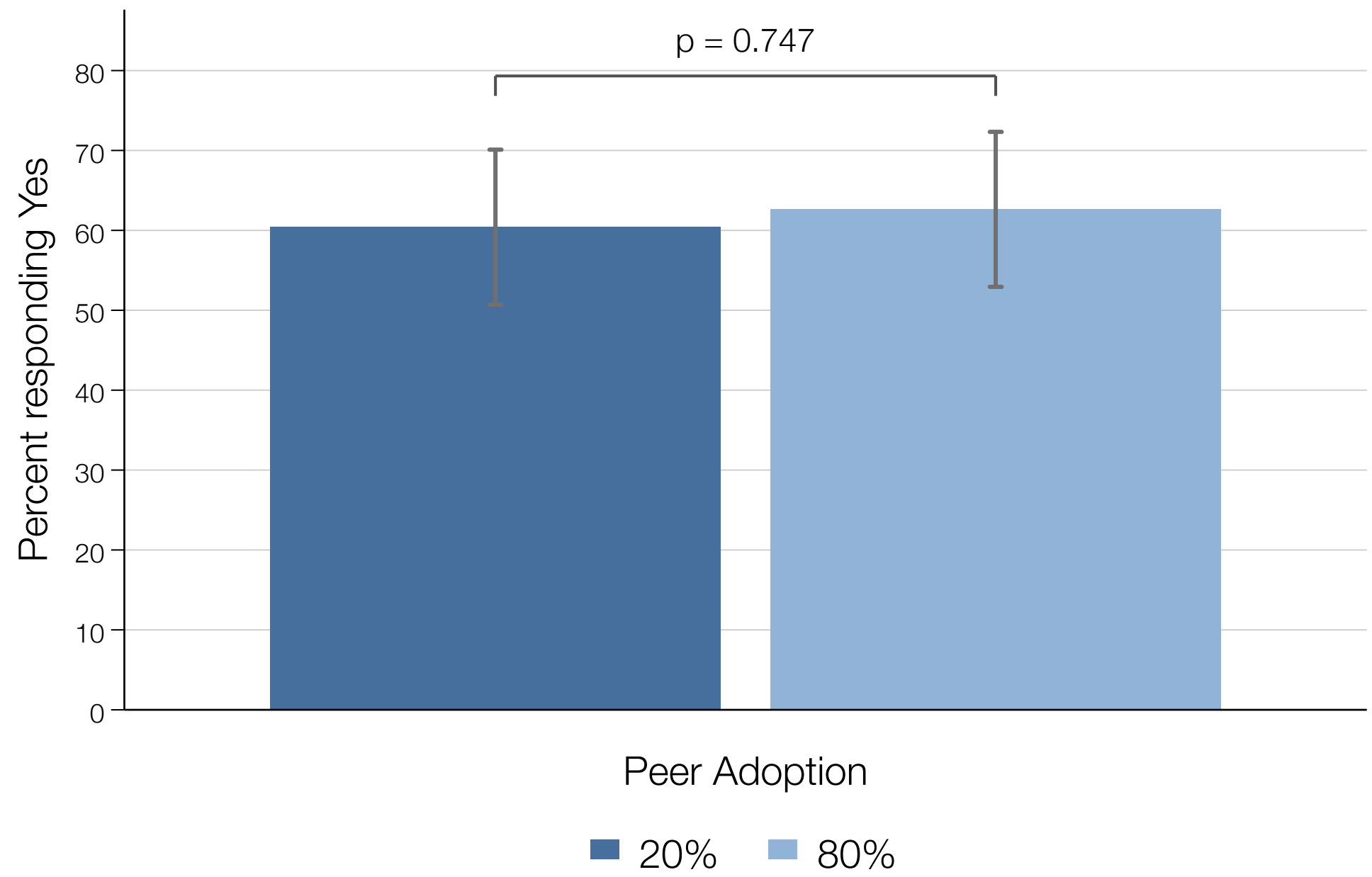


*Notes:* This figure displays the results of our third pre-registered experiment. After seeing the erosion information, respondents were asked to consider one of two hypothetical scenarios in which adoption of AI among teenagers is randomized between 20% and 80%. We then asked respondents (using language identical to the other experiments) whether they would support an AI ban in this scenario. The figure displays the percentage of respondents answering Yes, i.e. supporting a ban. The analysis uses data from Experiment 3, as defined in Methods, consisting of 200 respondents. Bars in dark blue represent the low-adoption condition (n=101); bars in light blue represent the high-adoption condition (n=99). The p-value is from a two-sided t-test. Error bars indicate 95% confidence intervals for the group means.

**Fig. S9. Difference in posterior beliefs.**

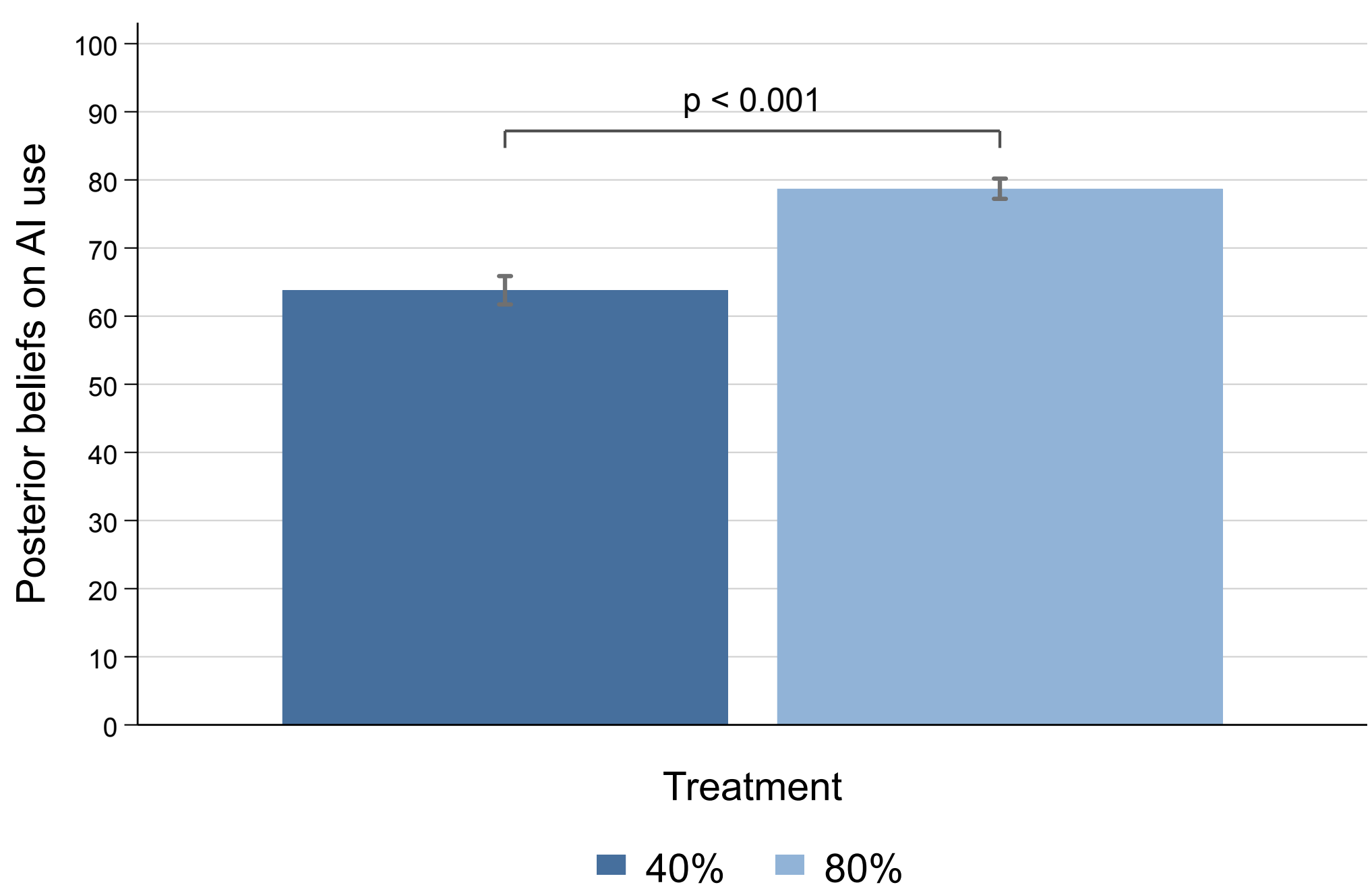


*Notes:* This figure displays the posterior beliefs regarding AI use from our fourth pre-registered experiment. After seeing the erosion information, respondents were shown one of two information treatments in which adoption of AI among teenagers is either 40% or 80%. At the end of the survey we ask participants about their belief on the percent of teenagers who use AI in their state to validate that our information treatment was effective. The analysis uses data from Experiment 4, as defined in Methods, consisting of 673 respondents. Bars in dark blue represent the low-adoption (40%) condition (n=335); bars in light blue represent the high-adoption (80%) condition (n=338). The p-value is from a two-sided t-test. Error bars indicate 95% confidence intervals for the group means.

**Fig. S10. Minimal evidence of quality updating and social learning**

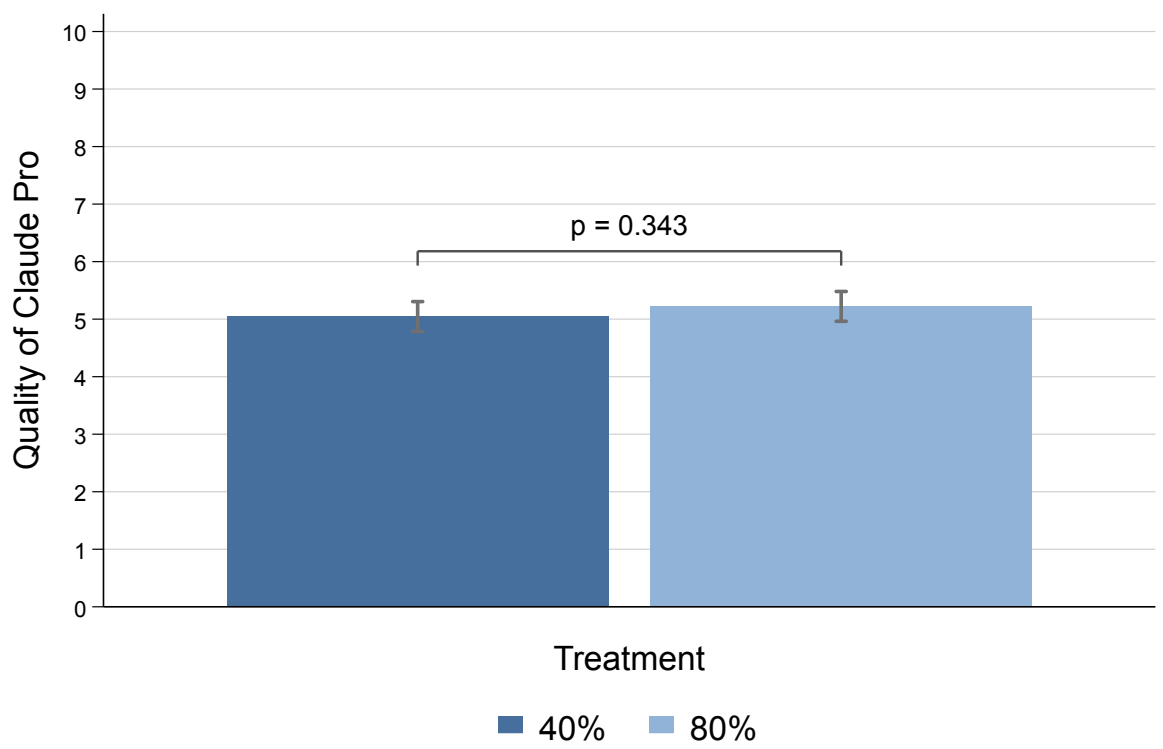


**(a)** Perceived Quality of Claude Pro

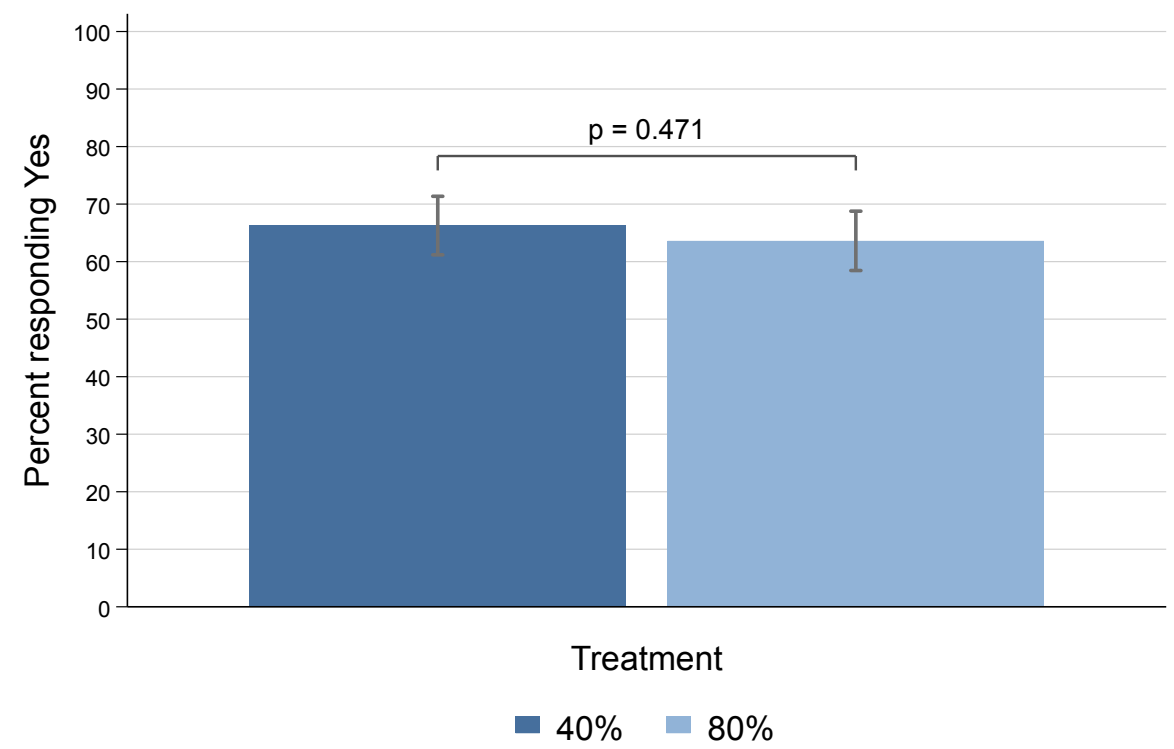


**(b)** Support for an AI Ban

*Notes:* This figure displays results from our fourth pre-registered experiment. Figure S10 Panel (a) shows average perceived quality of Claude Pro under two information treatments in which AI adoption among teenagers is either 40% or 80%. Panel (b) shows results for the same question about support for an AI ban from Experiment 3. The analysis uses data from Experiment 4, as defined in *Methods*, consisting of 673 respondents. Bars in dark blue represent the low-adoption (40%) condition ($n = 335$); bars in light blue represent the high-adoption (80%) condition ($n = 338$). The $p$-value is from a two-sided $t$-test. Error bars indicate 95% confidence intervals for the group means.

**Fig. S11. Minimal evidence of AI skill updating**

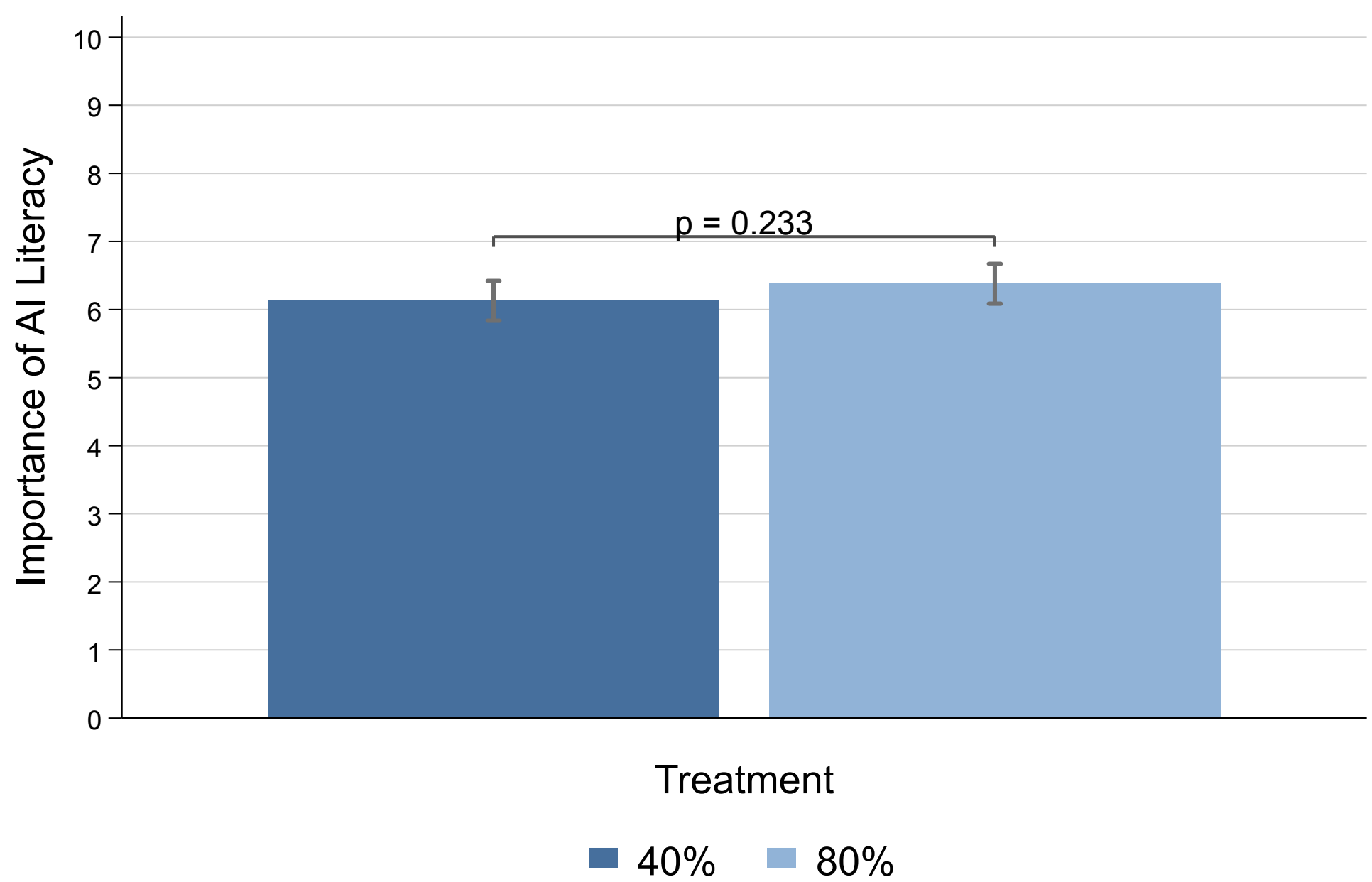


*Notes:* This figure displays results from our fourth pre-registered experiment. After seeing the erosion information, respondents were shown one of two information treatments in which adoption of AI among teenagers is either 40% or 80%. We collect the perceived importance of AI literacy. The analysis uses data from Experiment 4, as defined in Methods, consisting of 673 respondents. Bars in dark blue represent the low-adoption (40%) condition (n=335); bars in light blue represent the high-adoption (80%) condition (n=338). The p-value is from a two-sided t-test. Error bars indicate 95% confidence intervals for the group means.

**Fig. S12. Minimal evidence of general skill updating**

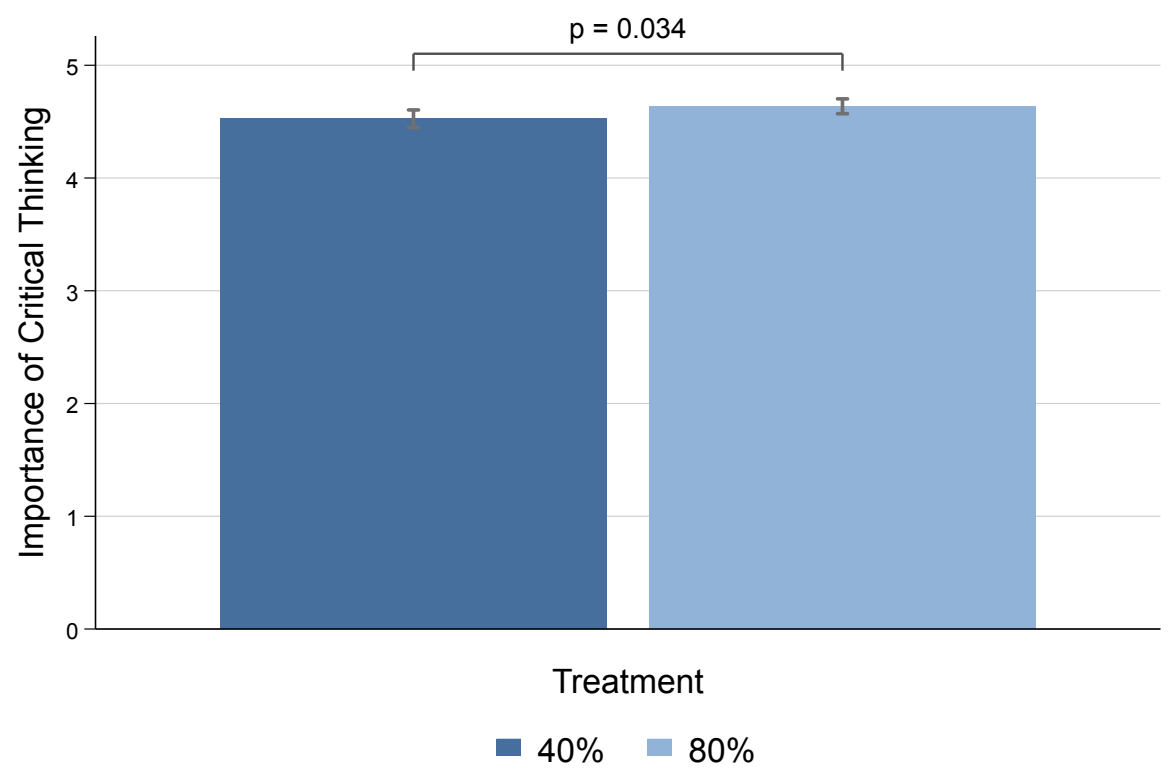


**(a)** Critical thinking

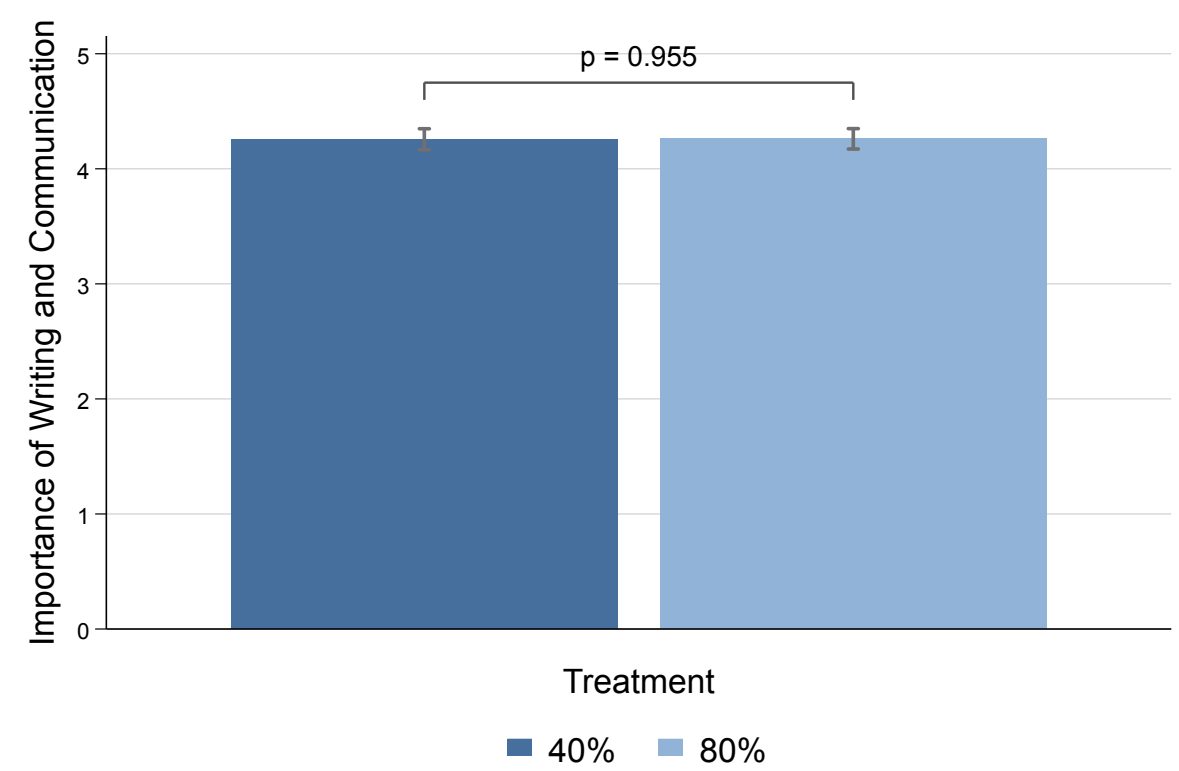


**(b)** Writing and communication

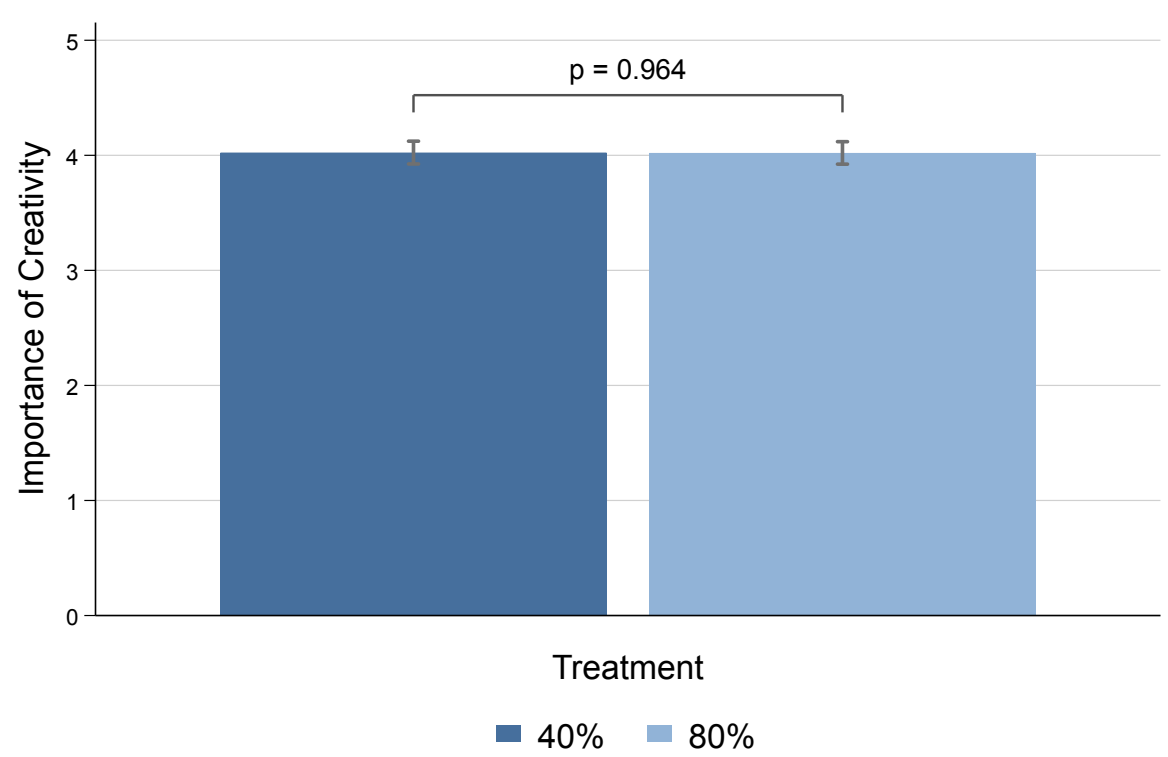


**(c)** Creativity

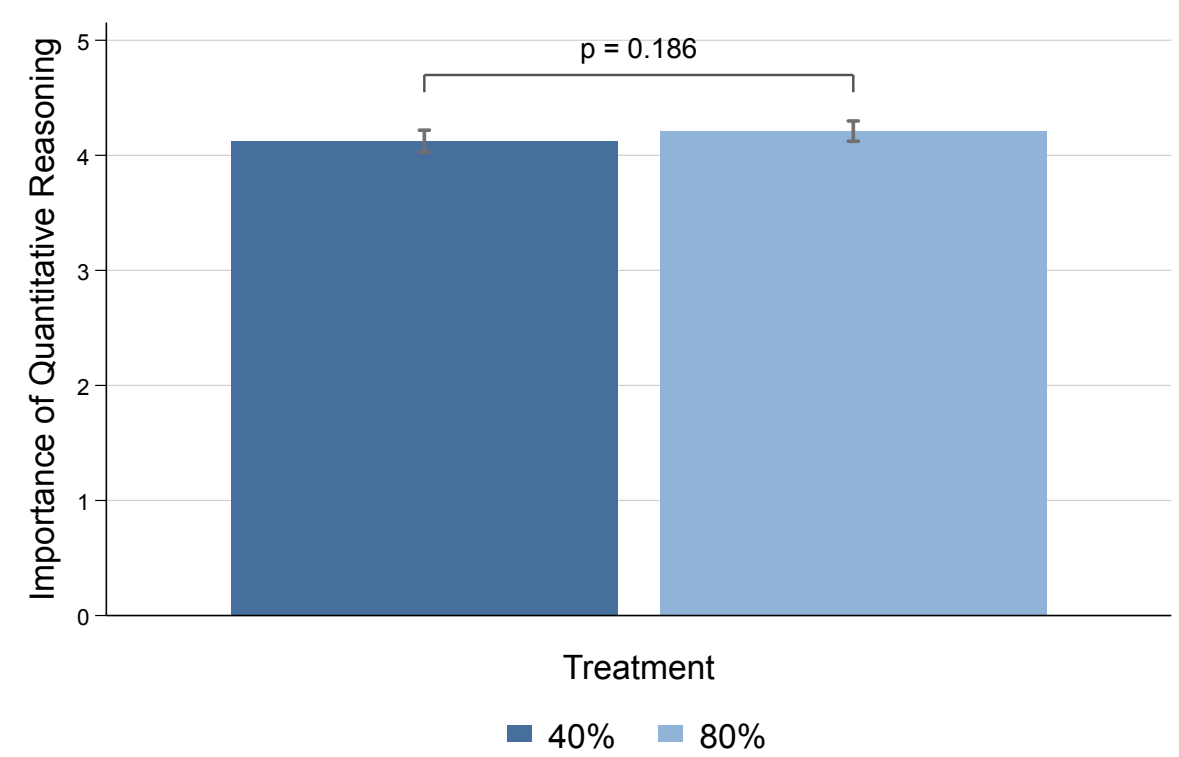


**(d)** Quantitative reasoning

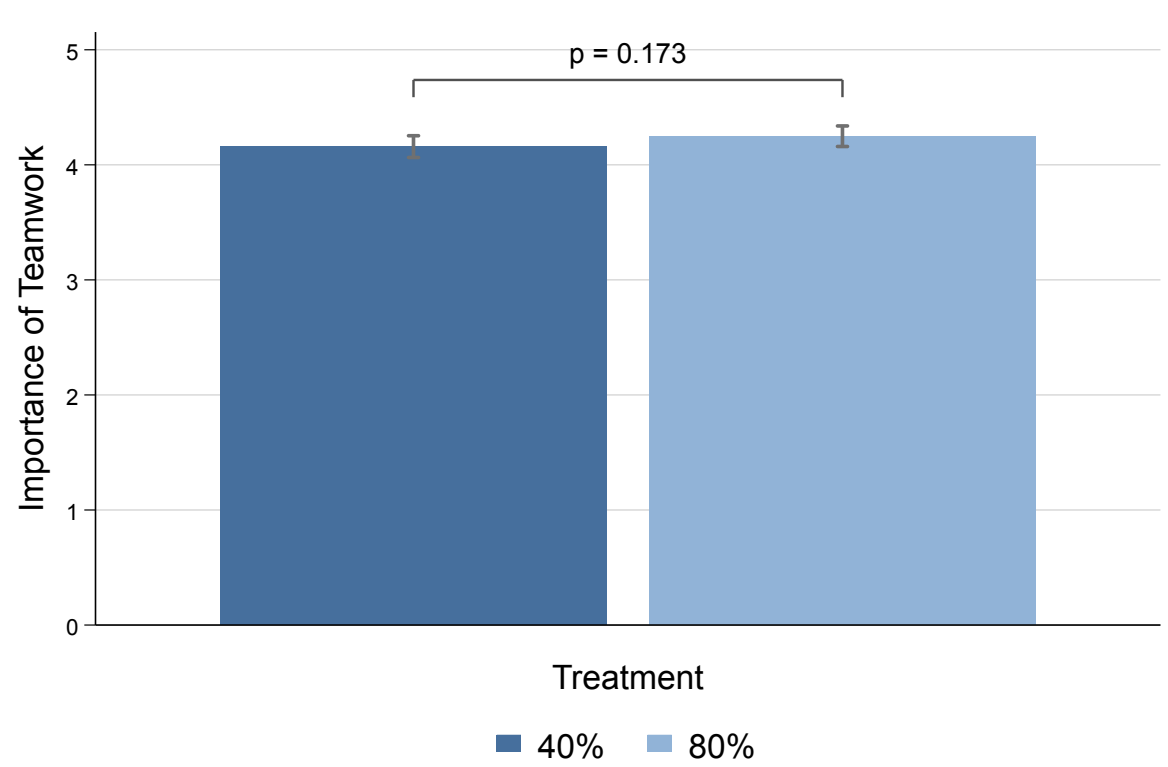


**(e)** Teamwork

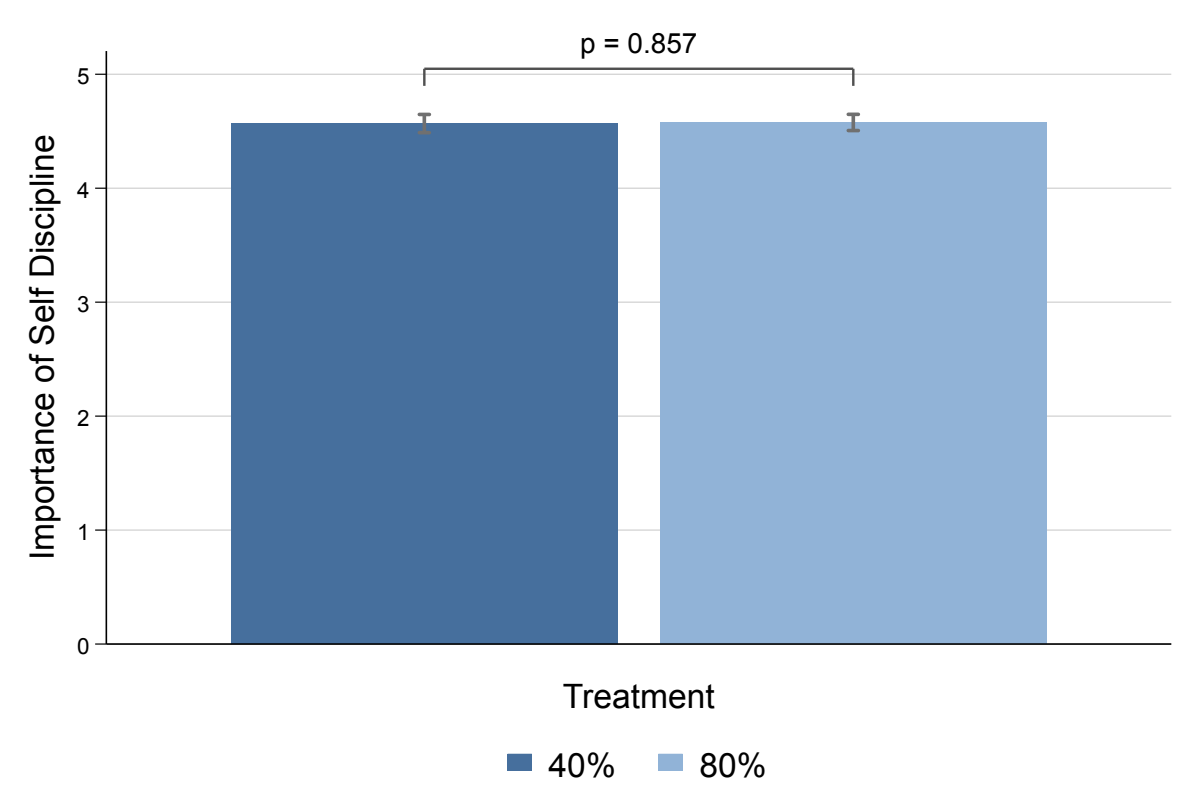


**(f)** Self discipline

*Notes:* This figure displays results from our fourth pre-registered experiment. Panels (a)–(f) display the perceived importance of long-term skills under two information treatments (low vs. high peer adoption). The analysis uses data from Experiment 4, as defined in *Methods*, consisting of 673 respondents. Bars in dark blue represent the low-adoption (40%) condition ($n = 335$); bars in light blue represent the high-adoption (80%) condition ($n = 338$). The $p$-value is from a two-sided $t$-test. Error bars indicate 95% confidence intervals for the group means.

345 ## 4. Supplementary Tables

**Table S1. Effect on willingness to pay: main experiment.**

| | (1) | (2) | (3) |
|---|---|---|---|
| Erosion Information | -0.359 | -0.359 | -0.367 |
| | (1.114) | (1.114) | (1.146) |
| Peer Adoption | | 0.183*** | 0.183*** |
| | | (0.009) | (0.012) |
| Erosion Information × Peer Adoption | | | 0.000 |
| | | | (0.017) |
| Observations | 3,972 | 3,972 | 3,972 |
| R-squared | 0.00 | 0.05 | 0.05 |
| Control mean | 22.88 | 22.88 | 22.88 |

**Table S2. Effect on willingness to pay: between subjects replication of main experiment.**

| | (1) | (2) | (3) |
|---|---|---|---|
| Erosion Information | -0.568 | -0.597 | -1.439 |
| | (1.858) | (1.837) | (3.559) |
| Peer Adoption | | 0.099*** | 0.091** |
| | | (0.031) | (0.043) |
| Erosion Information × Peer Adoption | | | 0.017 |
| | | | (0.061) |
| Observations | 410 | 410 | 410 |
| R-squared | 0.00 | 0.03 | 0.03 |
| Control mean | 22.86 | 22.86 | 22.86 |

**Table S3. Boundary conditions for social effect.**

| | (1) | (2) | (3) |
|---|---|---|---|
| Erosion information | -4.217*** | -4.217*** | -0.834 |
| | (1.063) | (1.063) | (1.094) |
| Peer Adoption | | 0.178*** | 0.209*** |
| | | (0.009) | (0.012) |
| Erosion information × Peer Adoption | | | -0.068*** |
| | | | (0.017) |
| Observations | 3,996 | 3,996 | 3,996 |
| R-squared | 0.01 | 0.06 | 0.06 |
| Control mean | 22.49 | 22.49 | 22.49 |

**Table S4. Effect of short-run information about benefits of AI.**

| | (1) |
|---|---|
| Peer Adoption | 0.142*** |
| | (0.012) |
| Short-Run Info | 2.159* |
| | (1.113) |
| Short-Run Info × Peer Adoption | 0.042** |
| | (0.017) |
| Observations | 3,812 |
| R-squared | 0.05 |
| Sample | Pooled |

**Table S5. Treatment conditions in Experiments 1 and 2.**

| | Treatment | Control |
|---|---|---|
| Exp. 1 | Short-Run Info + Erosion Info | Short-Run Info |
| Exp. 2 | Erosion Info | No Info |

**Table S6. Characteristics of sample for Experiment 1.**

| | Mean | Std. dev. | Median | Min | Max | Obs. |
|---|---|---|---|---|---|---|
| **Panel A**: Demographics | | | | | | |
| Age | 47.88 | 9.08 | 47 | 26 | 82 | 993 |
| % Female | 52.9 | 49.9 | 100 | 0 | 100 | 993 |
| *Panel A.1: Race and ethnicity* | | | | | | |
| % White | 77.6 | 41.7 | 100 | 0 | 100 | 993 |
| % Black or African American | 16.0 | 36.7 | 0 | 0 | 100 | 993 |
| % Asian | 3.5 | 18.4 | 0 | 0 | 100 | 993 |
| % Hispanic or Latino | 5.9 | 23.7 | 0 | 0 | 100 | 993 |
| *Panel A.2: Family* | | | | | | |
| Number of children 13-18 | 1.35 | 0.59 | 1 | 1 | 3 | 993 |
| **Panel B**: Parents' AI use | | | | | | |
| *Panel B.1: Tools* | | | | | | |
| % using ChatGPT | 83.6 | 37.1 | 100 | 0 | 100 | 993 |
| % using Gemini | 47.2 | 49.9 | 0 | 0 | 100 | 993 |
| % using Llama | 4.7 | 21.2 | 0 | 0 | 100 | 993 |
| % using Claude | 16.8 | 37.4 | 0 | 0 | 100 | 993 |
| % using other AI tools | 24.4 | 43.0 | 0 | 0 | 100 | 993 |
| *Panel B.2: Overall use* | | | | | | |
| % using AI tools | 94.5 | 22.9 | 100 | 0 | 100 | 993 |
| % using AI tools frequently | 47.5 | 50.0 | 0 | 0 | 100 | 993 |
| **Panel C**: Teenagers' AI use | | | | | | |
| *Panel C.1: Tools* | | | | | | |
| % using ChatGPT | 79.9 | 40.1 | 100 | 0 | 100 | 993 |
| % using Gemini | 39.9 | 49.0 | 0 | 0 | 100 | 993 |
| % using Llama | 3.1 | 17.4 | 0 | 0 | 100 | 993 |
| % using Claude | 9.9 | 29.8 | 0 | 0 | 100 | 993 |
| % using other AI tools | 11.8 | 32.3 | 0 | 0 | 100 | 993 |
| *Panel C.2: Overall use* | | | | | | |
| % using AI tools | 94.5 | 22.9 | 100 | 0 | 100 | 993 |
| % using AI tools frequently | 39.0 | 48.8 | 0 | 0 | 100 | 993 |

**Table S7. Characteristics of sample for Experiment 2.**

| | Mean | Std. dev. | Median | Min | Max | Obs. |
|---|---|---|---|---|---|---|
| **Panel A**: Demographics | | | | | | |
| Age | 48.44 | 8.44 | 48 | 15 | 80 | 999 |
| % Female | 52.3 | 50.0 | 100 | 0 | 100 | 999 |
| *Panel A.1: Race and ethnicity* | | | | | | |
| % White | 82.5 | 38.0 | 100 | 0 | 100 | 999 |
| % Black or African American | 8.7 | 28.2 | 0 | 0 | 100 | 999 |
| % Asian | 6.2 | 24.1 | 0 | 0 | 100 | 999 |
| % Hispanic or Latino | 2.5 | 15.6 | 0 | 0 | 100 | 999 |
| *Panel A.2: Family* | | | | | | |
| Number of children 13-18 | 1.32 | 0.54 | 1 | 1 | 3 | 999 |
| **Panel B**: Parents' AI use | | | | | | |
| *Panel B.1: Tools* | | | | | | |
| % using ChatGPT | 77.0 | 42.1 | 100 | 0 | 100 | 999 |
| % using Gemini | 36.4 | 48.1 | 0 | 0 | 100 | 999 |
| % using Llama | 3.0 | 17.1 | 0 | 0 | 100 | 999 |
| % using Claude | 7.3 | 26.0 | 0 | 0 | 100 | 999 |
| % using other AI tools | 21.2 | 40.9 | 0 | 0 | 100 | 999 |
| *Panel B.2: Overall use* | | | | | | |
| % using AI tools | 90.2 | 29.8 | 100 | 0 | 100 | 999 |
| % using AI tools frequently | 39.8 | 49.0 | 0 | 0 | 100 | 999 |
| **Panel C**: Teenagers' AI use | | | | | | |
| *Panel C.1: Tools* | | | | | | |
| % using ChatGPT | 70.9 | 45.5 | 100 | 0 | 100 | 999 |
| % using Gemini | 29.1 | 45.5 | 0 | 0 | 100 | 999 |
| % using Llama | 3.0 | 17.1 | 0 | 0 | 100 | 999 |
| % using Claude | 6.3 | 24.3 | 0 | 0 | 100 | 999 |
| % using other AI tools | 11.3 | 31.7 | 0 | 0 | 100 | 999 |
| *Panel C.2: Overall use* | | | | | | |
| % using AI tools | 90.9 | 28.8 | 100 | 0 | 100 | 999 |
| % using AI tools frequently | 34.4 | 47.5 | 0 | 0 | 100 | 999 |

**Table S8. Characteristics of sample for Experiment 3.**

| | Mean | Std. dev. | Median | Min | Max | Obs. |
|---|---|---|---|---|---|---|
| **Panel A**: Demographics | | | | | | |
| Age | 48.32 | 9.35 | 48 | 22 | 75 | 200 |
| % Female | 48.5 | 50.1 | 0 | 0 | 100 | 200 |
| *Panel A.1: Race and ethnicity* | | | | | | |
| % White | 80.5 | 39.7 | 100 | 0 | 100 | 200 |
| % Black or African American | 11.0 | 31.4 | 0 | 0 | 100 | 200 |
| % Asian | 4.0 | 19.6 | 0 | 0 | 100 | 200 |
| % Hispanic or Latino | 5.0 | 21.8 | 0 | 0 | 100 | 200 |
| *Panel A.2: Family* | | | | | | |
| Number of children 13-18 | 1.33 | 0.56 | 1 | 1 | 3 | 200 |

**Table S9. Characteristics of sample for Experiment 4.**

| | Mean | Std. dev. | Median | Min | Max | Obs. |
|---|---|---|---|---|---|---|
| **Panel A**: Demographics | | | | | | |
| Age | 46.76 | 8.06 | 46 | 26 | 75 | 673 |
| % Female | 59.9 | 49.1 | 100 | 0 | 100 | 673 |
| *Panel A.1: Race and ethnicity* | | | | | | |
| % White | 78.0 | 41.4 | 100 | 0 | 100 | 673 |
| % Black or African American | 14.3 | 35.0 | 0 | 0 | 100 | 673 |
| % Asian | 4.9 | 21.6 | 0 | 0 | 100 | 673 |
| % Hispanic or Latino | 7.3 | 26.0 | 0 | 0 | 100 | 673 |
| *Panel A.2: Family* | | | | | | |
| Number of children 13-18 | 1.36 | 0.57 | 1 | 1 | 3 | 673 |

**Table S10. Characteristics of sample for Experiment 5.**

| | Mean | Std. dev. | Median | Min | Max | Obs. |
|---|---|---|---|---|---|---|
| **Panel A**: Demographics | | | | | | |
| Age | 46.93 | 7.54 | 47 | 24 | 83 | 401 |
| % Female | 52.9 | 50.0 | 100 | 0 | 100 | 401 |
| *Panel A.1: Race and ethnicity* | | | | | | |
| % White | 84.3 | 36.4 | 100 | 0 | 100 | 401 |
| % Black or African American | 8.5 | 27.9 | 0 | 0 | 100 | 401 |
| % Asian | 5.7 | 23.3 | 0 | 0 | 100 | 401 |
| % Hispanic or Latino | 1.7 | 13.1 | 0 | 0 | 100 | 401 |
| *Panel A.2: Family* | | | | | | |
| Number of children 13-18 | 1.31 | 0.56 | 1 | 1 | 3 | 401 |
| **Panel B**: Parents' AI use | | | | | | |
| *Panel B.1: General-purpose LLMs* | | | | | | |
| % using ChatGPT | 83.8 | 36.9 | 100 | 0 | 100 | 401 |
| % using Gemini | 48.6 | 50.0 | 0 | 0 | 100 | 401 |
| % using Llama | 2.5 | 15.6 | 0 | 0 | 100 | 401 |
| % using Claude | 12.2 | 32.8 | 0 | 0 | 100 | 401 |
| % using other AI tools | 28.2 | 45.0 | 0 | 0 | 100 | 401 |
| *Panel B.2: Specialised AI tools* | | | | | | |
| % using tutoring tools (Khanmigo, etc.) | 0.5 | 7.1 | 0 | 0 | 100 | 401 |
| % using writing tools (Grammarly, etc.) | 13.0 | 33.6 | 0 | 0 | 100 | 401 |
| % using math solvers (Photomath, etc.) | 1.7 | 13.1 | 0 | 0 | 100 | 401 |
| *Panel B.3: Overall use* | | | | | | |
| % using AI tools | 97.0 | 17.1 | 100 | 0 | 100 | 401 |
| % using AI tools frequently | 51.9 | 50.0 | 100 | 0 | 100 | 401 |
| **Panel C**: Teenagers' AI use | | | | | | |
| *Panel C.1: General-purpose LLMs* | | | | | | |
| % using ChatGPT | 77.6 | 41.8 | 100 | 0 | 100 | 401 |
| % using Gemini | 33.7 | 47.3 | 0 | 0 | 100 | 401 |
| % using Llama | 1.0 | 9.9 | 0 | 0 | 100 | 401 |
| % using Claude | 3.5 | 18.4 | 0 | 0 | 100 | 401 |
| % using other AI tools | 8.5 | 27.9 | 0 | 0 | 100 | 401 |
| *Panel C.2: Specialised AI tools* | | | | | | |
| % using tutoring tools (Khanmigo, etc.) | 7.5 | 26.3 | 0 | 0 | 100 | 401 |
| % using writing tools (Grammarly, etc.) | 25.4 | 43.6 | 0 | 0 | 100 | 401 |
| % using math solvers (Photomath, etc.) | 13.5 | 34.2 | 0 | 0 | 100 | 401 |
| *Panel C.3: Overall use* | | | | | | |
| % using AI tools | 94.3 | 23.3 | 100 | 0 | 100 | 401 |
| % using AI tools frequently | 34.2 | 47.5 | 0 | 0 | 100 | 401 |

**Table S11. Characteristics of sample for Experiment 6.**

| | Mean | Std. dev. | Median | Min | Max | Obs. |
|---|---|---|---|---|---|---|
| **Panel A**: Demographics | | | | | | |
| Age | 46.42 | 7.53 | 46 | 31 | 76 | 202 |
| % Female | 66.8 | 47.2 | 100 | 0 | 100 | 202 |
| *Panel A.1: Race and ethnicity* | | | | | | |
| % White | 78.2 | 41.4 | 100 | 0 | 100 | 202 |
| % Black or African American | 8.9 | 28.6 | 0 | 0 | 100 | 202 |
| % Asian | 10.9 | 31.2 | 0 | 0 | 100 | 202 |
| % Hispanic or Latino | 3.0 | 17.0 | 0 | 0 | 100 | 202 |
| *Panel A.2: Family* | | | | | | |
| Number of children 13-18 | 1.23 | 0.50 | 1 | 1 | 3 | 202 |
| **Panel B**: Parents' AI use | | | | | | |
| *Panel B.1: General-purpose LLMs* | | | | | | |
| % using ChatGPT | 83.2 | 37.5 | 100 | 0 | 100 | 202 |
| % using Gemini | 46.5 | 50.0 | 0 | 0 | 100 | 202 |
| % using Llama | 2.0 | 14.0 | 0 | 0 | 100 | 202 |
| % using Claude | 12.4 | 33.0 | 0 | 0 | 100 | 202 |
| % using other AI tools | 20.8 | 40.7 | 0 | 0 | 100 | 202 |
| *Panel B.2: Specialised AI tools* | | | | | | |
| % using tutoring tools (Khanmigo, etc.) | 1.5 | 12.1 | 0 | 0 | 100 | 202 |
| % using writing tools (Grammarly, etc.) | 14.9 | 35.6 | 0 | 0 | 100 | 202 |
| % using math solvers (Photomath, etc.) | 3.0 | 17.0 | 0 | 0 | 100 | 202 |
| *Panel B.3: Overall use* | | | | | | |
| % using AI tools | 95.5 | 20.7 | 100 | 0 | 100 | 202 |
| % using AI tools frequently | 56.9 | 49.6 | 100 | 0 | 100 | 202 |
| **Panel C**: Teenagers' AI use | | | | | | |
| *Panel C.1: General-purpose LLMs* | | | | | | |
| % using ChatGPT | 79.2 | 40.7 | 100 | 0 | 100 | 202 |
| % using Gemini | 30.7 | 46.2 | 0 | 0 | 100 | 202 |
| % using Llama | 0.5 | 7.0 | 0 | 0 | 100 | 202 |
| % using Claude | 2.5 | 15.6 | 0 | 0 | 100 | 202 |
| % using other AI tools | 9.4 | 29.3 | 0 | 0 | 100 | 202 |
| *Panel C.2: Specialised AI tools* | | | | | | |
| % using tutoring tools (Khanmigo, etc.) | 8.4 | 27.8 | 0 | 0 | 100 | 202 |
| % using writing tools (Grammarly, etc.) | 30.7 | 46.2 | 0 | 0 | 100 | 202 |
| % using math solvers (Photomath, etc.) | 10.9 | 31.2 | 0 | 0 | 100 | 202 |
| *Panel C.3: Overall use* | | | | | | |
| % using AI tools | 90.6 | 29.3 | 100 | 0 | 100 | 202 |
| % using AI tools frequently | 39.6 | 49.0 | 0 | 0 | 100 | 202 |

**Table S12. Characteristics of sample for Experiment 7.**

| | Mean | Std. dev. | Median | Min | Max | Obs. |
|---|---|---|---|---|---|---|
| **Panel A**: Demographics | | | | | | |
| Age | 47.59 | 7.99 | 47 | 30 | 82 | 186 |
| % Female | 47.3 | 50.1 | 0 | 0 | 100 | 186 |
| *Panel A.1: Race and ethnicity* | | | | | | |
| % White | 82.3 | 38.3 | 100 | 0 | 100 | 186 |
| % Black or African American | 10.2 | 30.4 | 0 | 0 | 100 | 186 |
| % Asian | 10.2 | 30.4 | 0 | 0 | 100 | 186 |
| % Hispanic or Latino | 2.2 | 14.5 | 0 | 0 | 100 | 186 |
| *Panel A.2: Family* | | | | | | |
| Number of children 13-18 | 1.34 | 0.59 | 1 | 1 | 3 | 186 |
| **Panel B**: Parents' AI use | | | | | | |
| *Panel B.1: Tools* | | | | | | |
| % using ChatGPT | 79.6 | 40.4 | 100 | 0 | 100 | 186 |
| % using Gemini | 58.1 | 49.5 | 100 | 0 | 100 | 186 |
| % using Llama | 1.6 | 12.6 | 0 | 0 | 100 | 186 |
| % using Claude | 22.6 | 41.9 | 0 | 0 | 100 | 186 |
| % using other AI tools | 29.0 | 45.5 | 0 | 0 | 100 | 186 |
| *Panel B.2: Overall use* | | | | | | |
| % using AI tools | 95.7 | 20.3 | 100 | 0 | 100 | 186 |
| % using AI tools frequently | 62.9 | 48.4 | 100 | 0 | 100 | 186 |
| **Panel C**: Teenagers' AI use | | | | | | |
| *Panel C.1: Tools* | | | | | | |
| % using ChatGPT | 74.2 | 43.9 | 100 | 0 | 100 | 186 |
| % using Gemini | 44.1 | 49.8 | 0 | 0 | 100 | 186 |
| % using Llama | 0.0 | 0.0 | 0 | 0 | 0 | 186 |
| % using Claude | 10.2 | 30.4 | 0 | 0 | 100 | 186 |
| % using other AI tools | 9.1 | 28.9 | 0 | 0 | 100 | 186 |
| *Panel C.2: Overall use* | | | | | | |
| % using AI tools | 90.3 | 29.6 | 100 | 0 | 100 | 186 |
| % using AI tools frequently | 36.6 | 48.3 | 0 | 0 | 100 | 186 |

**Table S13. Characteristics of sample for Experiment 8.**

| | Mean | Std. dev. | Median | Min | Max | Obs. |
|---|---|---|---|---|---|---|
| **Panel A**: Demographics | | | | | | |
| Age | 46.59 | 7.62 | 46 | 24 | 74 | 410 |
| % Female | 55.4 | 49.8 | 100 | 0 | 100 | 410 |
| *Panel A.1: Race and ethnicity* | | | | | | |
| % White | 76.3 | 42.6 | 100 | 0 | 100 | 410 |
| % Black or African American | 12.4 | 33.0 | 0 | 0 | 100 | 410 |
| % Asian | 7.3 | 26.1 | 0 | 0 | 100 | 410 |
| % Hispanic or Latino | 6.3 | 24.4 | 0 | 0 | 100 | 410 |
| *Panel A.2: Family* | | | | | | |
| Number of children 13-18 | 1.34 | 0.56 | 1 | 1 | 3 | 410 |
| **Panel B**: Parents' AI use | | | | | | |
| *Panel B.1: Tools* | | | | | | |
| % using ChatGPT | 84.6 | 36.1 | 100 | 0 | 100 | 410 |
| % using Gemini | 62.9 | 48.4 | 100 | 0 | 100 | 410 |
| % using Llama | 2.7 | 16.2 | 0 | 0 | 100 | 410 |
| % using Claude | 29.3 | 45.6 | 0 | 0 | 100 | 410 |
| % using other AI tools | 30.7 | 46.2 | 0 | 0 | 100 | 410 |
| *Panel B.2: Overall use* | | | | | | |
| % using AI tools | 97.6 | 15.4 | 100 | 0 | 100 | 410 |
| % using AI tools frequently | 65.4 | 47.6 | 100 | 0 | 100 | 410 |
| **Panel C**: Teenagers' AI use | | | | | | |
| *Panel C.1: Tools* | | | | | | |
| % using ChatGPT | 76.1 | 42.7 | 100 | 0 | 100 | 410 |
| % using Gemini | 51.0 | 50.1 | 100 | 0 | 100 | 410 |
| % using Llama | 1.0 | 9.8 | 0 | 0 | 100 | 410 |
| % using Claude | 14.4 | 35.1 | 0 | 0 | 100 | 410 |
| % using other AI tools | 15.4 | 36.1 | 0 | 0 | 100 | 410 |
| *Panel C.2: Overall use* | | | | | | |
| % using AI tools | 93.9 | 24.0 | 100 | 0 | 100 | 410 |
| % using AI tools frequently | 43.9 | 49.7 | 0 | 0 | 100 | 410 |

**Table S14. Overview of coding scheme for open-ended responses.**

| Category | Definition | Examples |
|---|---|---|
| Belief Only | The response is a positive statement (e.g. a belief or assertion about facts), but does not explain why the respondent allows their teenager to use AI. | "I think it improves their thinking skills to not use AI" *or* "AI is helpful but it has also caused students to become lazy." |
| Social Pressure | The respondent allows AI use only because, regardless of their feelings about AI, these tools exist and others will use them, and thus it is individually optimal for their child to use them, too. The response is consistent with concerns that the existence or widespread use of AI imposes a negative externality on non-users, e.g. being at a disadvantage in school or later on the job market. | "Since AI is so common now, I think my teenager should know how to use it" *or* "They feel that all the other students are using it, so they need to use it to keep up" *or* "Because I know that in this day and age AI tools are socially acceptable and I feel that there is some instances where it's almost required in the same age." |
| Don't Control | The respondent says they cannot or do not control their child's AI use, due to age, independence, or context. | "She wants to" *or* "It is here and there are areas out of my control that allow its use, like school" *or* "I don't let her use it, she uses it without my knowledge at school." |
| Judgment Only | The response is a normative statement (e.g. a preference, imperative or value judgment), but does not explain why the respondent allows their teenager to use AI. | "Students need to learn critical thinking skills" *or* "AI can be helpful at times. However, it should not be relied upon excessively for information." |
| Limited Use | The respondent allows AI use in a restricted way, under supervision, or for specific tasks, with the implication that this is supposed to be less harmful to the child than unfettered AI use. | "I let them use it when we are answering questions but I do not let them use it for actual school work being turned in" *or* "That doesn't mean that they can never use it just not for everything, everyday." |
| Non-Academic Uses | The respondent allows AI use for non-school tasks such as hobbies, creativity, or fun—but not for academic work. | "She uses it for resumes because she is job hunting" *or* "I do not allow them to use it for school. They use it for creative development outside of school." |
| Required at School | The respondent allows AI use because it is assigned or required by school, teachers, or curriculum. | "Sometimes his school has a project where they have them us AI on purpose" *or* "She has too for school I personally think it makes them dumber." |

## 5. Experimental Instructions

### A. Experiment 1.

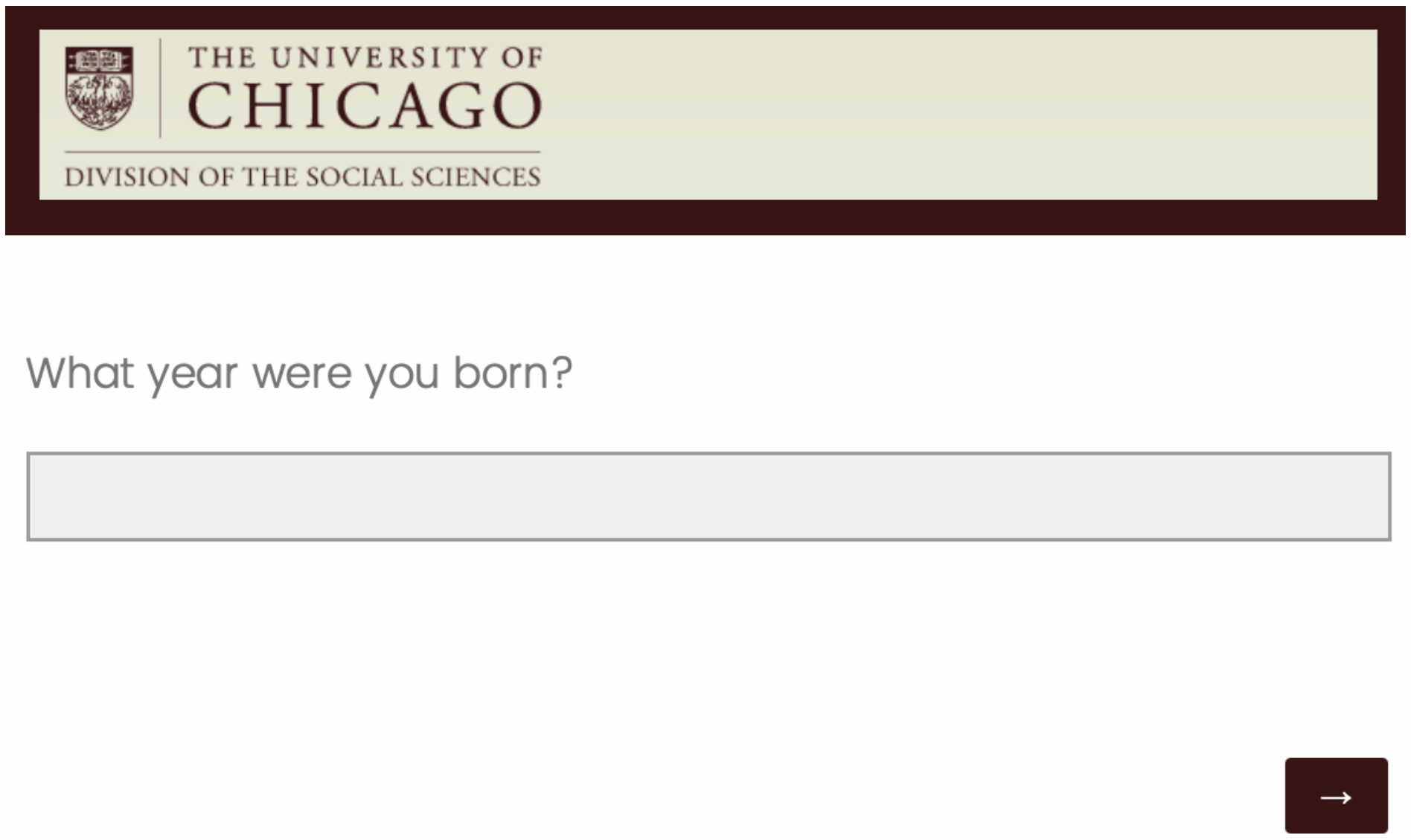


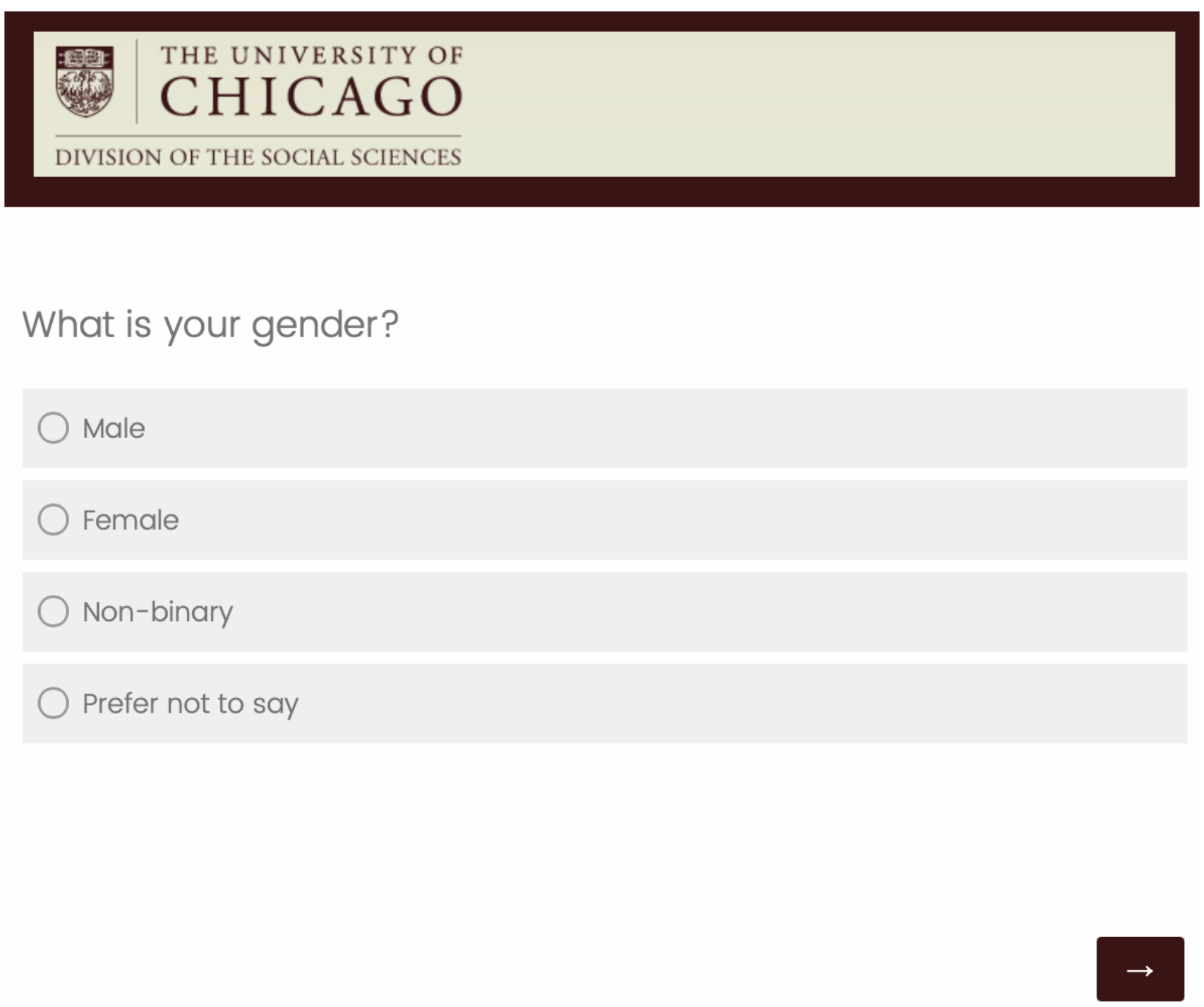

THE UNIVERSITY OF CHICAGO
DIVISION OF THE SOCIAL SCIENCES

What is your race/ethnicity? (Select all that apply)

- ☐ Hispanic or Latino
- ☐ White
- ☐ Black or African American
- ☐ Asian
- ☐ Native American or Alaska Native
- ☐ Native Hawaiian or Pacific Islander
- ☐ Other

→

THE UNIVERSITY OF CHICAGO
DIVISION OF THE SOCIAL SCIENCES

How many children do you have in each age group?

| | 0 | 1 | 2 | 3 or more |
|---|---|---|---|---|
| 0-4 years | ○ | ○ | ○ | ○ |
| 5-12 years | ○ | ○ | ○ | ○ |
| 13-18 years | ○ | ○ | ○ | ○ |

→

THE UNIVERSITY OF CHICAGO
DIVISION OF THE SOCIAL SCIENCES

What is the gender of your children? (Select all that apply)

- ☐ Male
- ☐ Female
- ☐ Non-binary
- ☐ Prefer not to say
- ☐ I do not have children

THE UNIVERSITY OF CHICAGO
DIVISION OF THE SOCIAL SCIENCES

Please consider only the children you have who are between 13-18 years old.

THE UNIVERSITY OF CHICAGO
DIVISION OF THE SOCIAL SCIENCES

Which of the following AI tools do **your teenagers** use? (Select all that apply)

- ☐ ChatGPT
- ☐ Claude
- ☐ Gemini
- ☐ Llama
- ☐ Others
- ☐ Not sure
- ☐ None

→

THE UNIVERSITY OF CHICAGO
DIVISION OF THE SOCIAL SCIENCES

How frequently do **your teenagers** use AI tools?

- ○ Never
- ○ Rarely
- ○ Occasionally
- ○ Frequently
- ○ Very frequently

→

THE UNIVERSITY OF CHICAGO
DIVISION OF THE SOCIAL SCIENCES

Which of the following AI tools do **you** use? (Select all that apply)

- ☐ ChatGPT
- ☐ Claude
- ☐ Gemini
- ☐ Llama
- ☐ Others
- ☐ Not sure
- ☐ None

→

THE UNIVERSITY OF CHICAGO
DIVISION OF THE SOCIAL SCIENCES

How frequently do **you** use AI tools?

- ○ Never
- ○ Rarely
- ○ Occasionally
- ○ Frequently
- ○ Very frequently

→

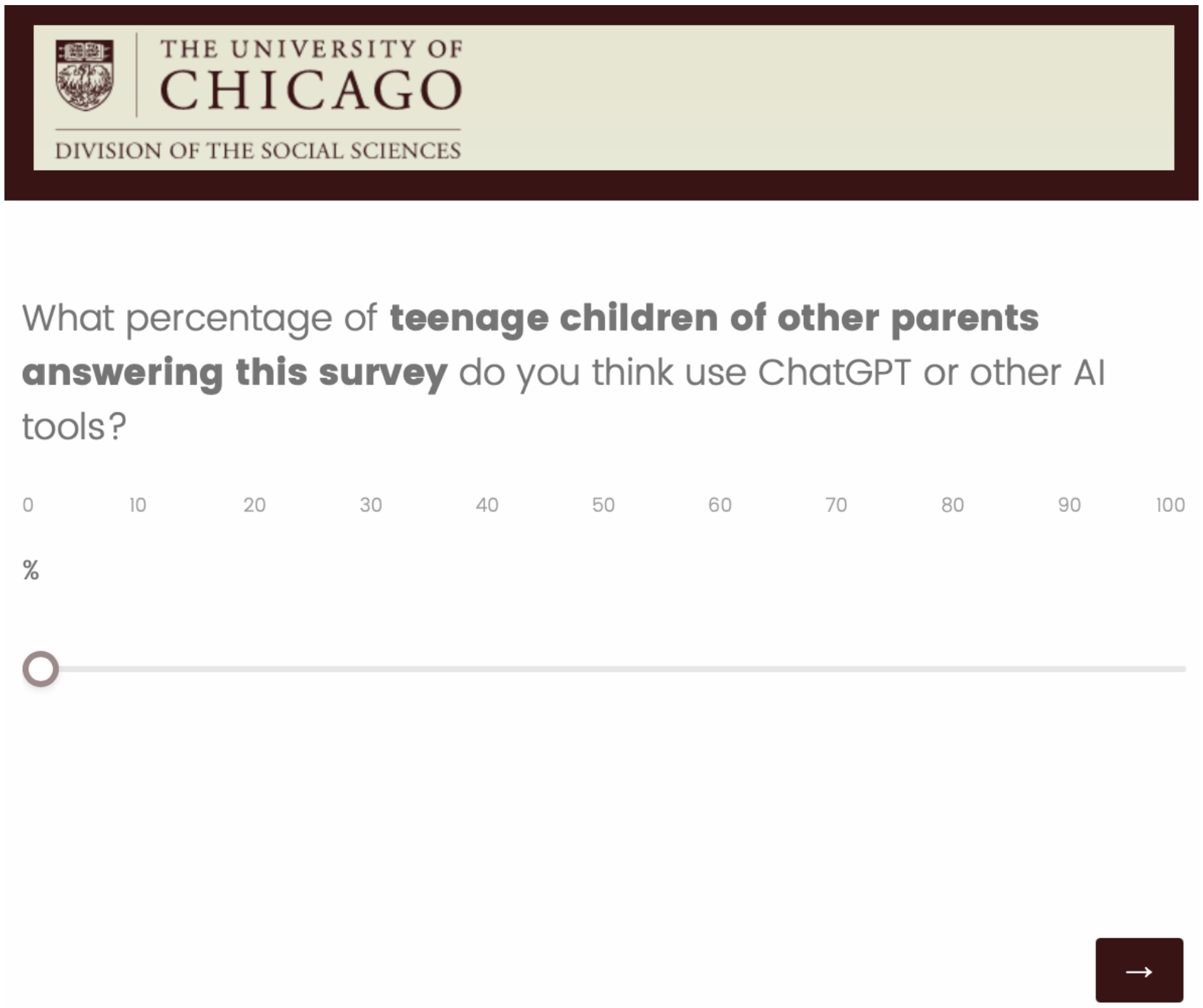
THE UNIVERSITY OF
CHICAGO
DIVISION OF THE SOCIAL SCIENCES
What percentage of **teenage children of other parents answering this survey** do you think use ChatGPT or other AI tools?
0 10 20 30 40 50 60 70 80 90 100
%
→

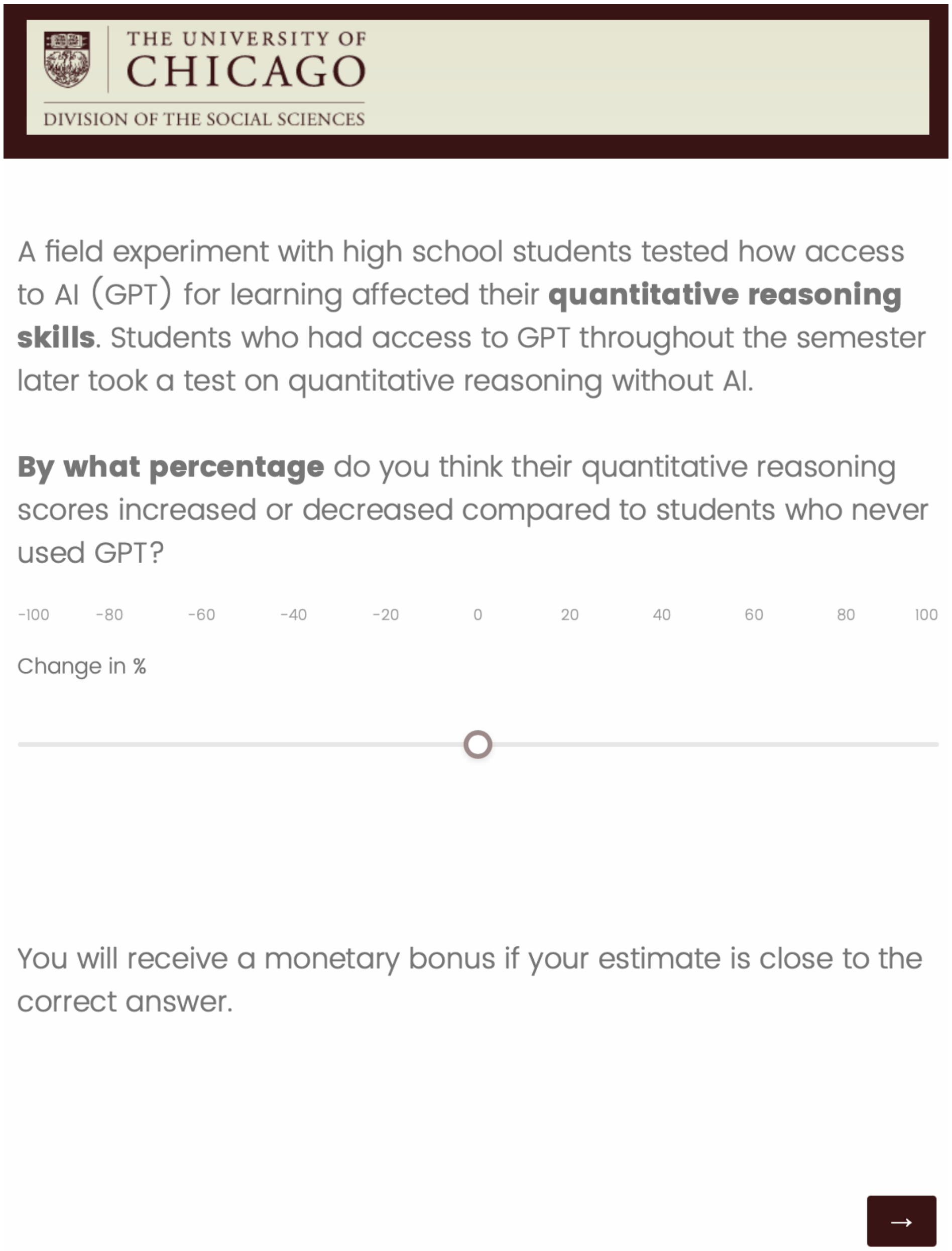

A field experiment with high school students tested how access to AI (GPT) for learning affected their **quantitative reasoning skills**. Students who had access to GPT throughout the semester later took a test on quantitative reasoning without AI.

**By what percentage** do you think their quantitative reasoning scores increased or decreased compared to students who never used GPT?

-100 -80 -60 -40 -20 0 20 40 60 80 100

Change in %

You will receive a monetary bonus if your estimate is close to the correct answer.

THE UNIVERSITY OF CHICAGO
DIVISION OF THE SOCIAL SCIENCES

In this part of the survey you will have the chance to win a three-month subscription to Claude Pro, currently valued at $60. The subscription is intended for your teenager's use, e.g., to help with homework, improve grammar and language, or search for information.

→

THE UNIVERSITY OF CHICAGO
DIVISION OF THE SOCIAL SCIENCES

We will allocate subscriptions using the following procedure: One out of every 100 participants will be randomly chosen to win either the Claude Pro subscription or a bonus payment. We will ask you to tell us the maximum bonus (in dollars) that you would be willing to give up to obtain the Claude Pro subscription. This is **your valuation of the Claude Pro subscription** for your teenager.

If you are selected, the computer will randomly generate a bonus to give you.

- If your valuation is higher than the computer's bonus, we will give you the subscription for your teenager.
- If your valuation is lower than the computer's bonus, we will give you the bonus.

As long as you truthfully state your valuation, this mechanism ensures that you receive the prize you actually prefer.

→

THE UNIVERSITY OF CHICAGO
DIVISION OF THE SOCIAL SCIENCES

Which of the following statements is **true** about how the Claude Pro subscription or bonus will be allocated?

- ○ Entering a higher valuation for the Claude Pro subscription makes it **less likely** that I will receive the subscription and more likely that I will receive the bonus.
- ○ The amount I am willing to forgo for the Claude Pro subscription **does not affect** whether I receive the subscription or the bonus.
- ○ Entering a higher valuation for the Claude Pro subscription makes it **more likely** that I will receive the subscription and less likely that I will receive the bonus.

→

THE UNIVERSITY OF CHICAGO

DIVISION OF THE SOCIAL SCIENCES

We are conducting this survey with a large population of parents with teenagers. From this survey, we will be able to estimate what percentage of teenagers use AI tools for things like schoolwork and research.

We would like you to consider your decision to get Claude Pro for your teenager **across different scenarios** that depend on how many other teenagers also use similar technology. For example, we will ask you how much you would be willing to pay for Claude Pro for your teenager in the case that 40% of other teenagers use and have access to AI tools.

Once we have collected all survey responses, we will determine the scenario that comes closest to the actual percentage of teenagers using AI in our sample. If you are randomly chosen to receive a reward, then whether you win the Claude Pro subscription or the bonus will be determined by your answer for this scenario only.

→

THE UNIVERSITY OF CHICAGO
DIVISION OF THE SOCIAL SCIENCES

Recent research has shown that using advanced AI models helps improve performance on writing and language tasks **while it is being used**.

On the other hand, a field experiment with high school students found that access to GPT for learning led to a **nearly 20% decline** in quantitative reasoning scores when students were later tested without AI.

Why did scores decline?

- **Over-reliance on AI:** Students used GPT to generate ideas rather than engaging in problem-solving themselves.
- **Weaker mental effort:** They became less practiced in structuring arguments and reasoning independently.
- **Reduced cognitive engagement:** AI-assisted learning replaced the effort required to develop analytical thinking.

THE UNIVERSITY OF CHICAGO
DIVISION OF THE SOCIAL SCIENCES

Recent research has shown that using advanced AI models helps improve performance on writing and language tasks **while it is being used**.

You will now enter how much you would be willing to pay (by forgoing your bonus) for a three-month subscription to Claude Pro for your teenager under a series of scenarios. The scenarios will include information on how many other teenagers in our sample - of the same age and from the same state as your own - use AI.

If **20% of teenagers** use AI, how much would you pay for a Claude Pro subscription for your teenager?

If **40% of teenagers** use AI, how much would you pay for a Claude Pro subscription for your teenager?

If **60% of teenagers** use AI, how much would you pay for a Claude Pro subscription for your teenager?

If **80% of teenagers** use AI, how much would you pay for a Claude Pro subscription for your teenager?

→

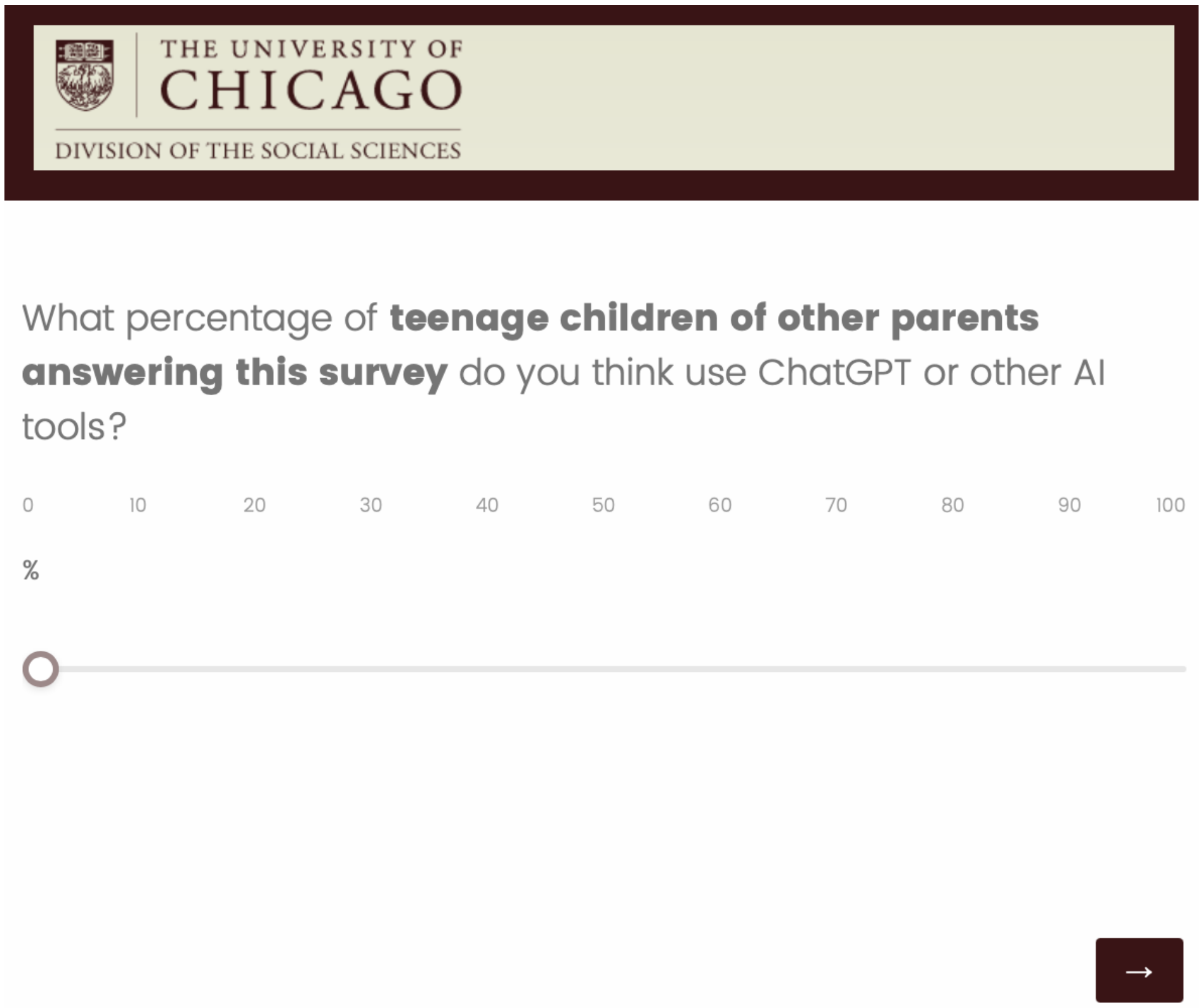
THE UNIVERSITY OF CHICAGO
DIVISION OF THE SOCIAL SCIENCES
What percentage of **teenage children of other parents answering this survey** do you think use ChatGPT or other AI tools?
0
10
20
30
40
50
60
70
80
90
100
%
→

THE UNIVERSITY OF CHICAGO

DIVISION OF THE SOCIAL SCIENCES

For each percentage below, please indicate **how likely** you think it is that this percentage comes closest to the actual share of teenagers using AI in our sample.

(0 = Very Unlikely, 100 = Very Likely)

0 10 20 30 40 50 60 70 80 90 100

20%

40%

60%

80%

→

THE UNIVERSITY OF CHICAGO
DIVISION OF THE SOCIAL SCIENCES

A different field experiment with college undergraduates tested how access to AI (GPT) for learning affected their **critical thinking skills**. Students who had access to GPT throughout the semester later took a test on critical thinking without AI.

**By what percentage** do you think their critical thinking scores increased or decreased compared to students who never used GPT?

-100 -80 -60 -40 -20 0 20 40 60 80 100

Change in %

You will receive a monetary bonus if your estimate is close to the correct answer.

→

THE UNIVERSITY OF CHICAGO
DIVISION OF THE SOCIAL SCIENCES

Would you prefer if no student was allowed to use AI tools and instead learned the way they used to before AI tools were introduced?

- ◯ Yes
- ◯ No

→

THE UNIVERSITY OF CHICAGO
DIVISION OF THE SOCIAL SCIENCES

You indicated that you would prefer if students were not allowed to use AI. You also indicated that you let your teenager use AI at least sometimes. Why is that?

→

THE UNIVERSITY OF CHICAGO
DIVISION OF THE SOCIAL SCIENCES

Do you agree with the following statement:
"I think AI tools will help my child now, but I'm worried about the long-term impact using AI tools will have on my child."

- ◯ Yes
- ◯ No

→

**B. Experiment 2.** The survey instructions are identical to those of experiment 1, with the sole exception of the information treatments. Experiment 1 has an active control, featuring information about short-run benefits of AI use which is shown to both groups. Experiment 2 drops this short-run information from the info screen:

THE UNIVERSITY OF CHICAGO
DIVISION OF THE SOCIAL SCIENCES

A field experiment with high school students found that access to GPT for learning led to a **nearly 20% decline** in quantitative reasoning scores when students were later tested without AI.

Why did scores decline?

- **Over-reliance on AI:** Students used GPT to generate ideas rather than engaging in problem-solving themselves.
- **Weaker mental effort:** They became less practiced in structuring arguments and reasoning independently.
- **Reduced cognitive engagement:** AI-assisted learning replaced the effort required to develop analytical thinking.

You will now enter how much you would be willing to pay (by forgoing your bonus) for a three-month subscription to Claude Pro for your teenager under a series of scenarios. The scenarios will include information on how many other teenagers in our sample - of the same age and from the same state as your own - use AI.

THE UNIVERSITY OF CHICAGO
DIVISION OF THE SOCIAL SCIENCES

You will now enter how much you would be willing to pay (by forgoing your bonus) for a three-month subscription to Claude Pro for your teenager under a series of scenarios. The scenarios will include information on how many other teenagers in our sample - of the same age and from the same state as your own - use AI.

### C. Experiment 3.

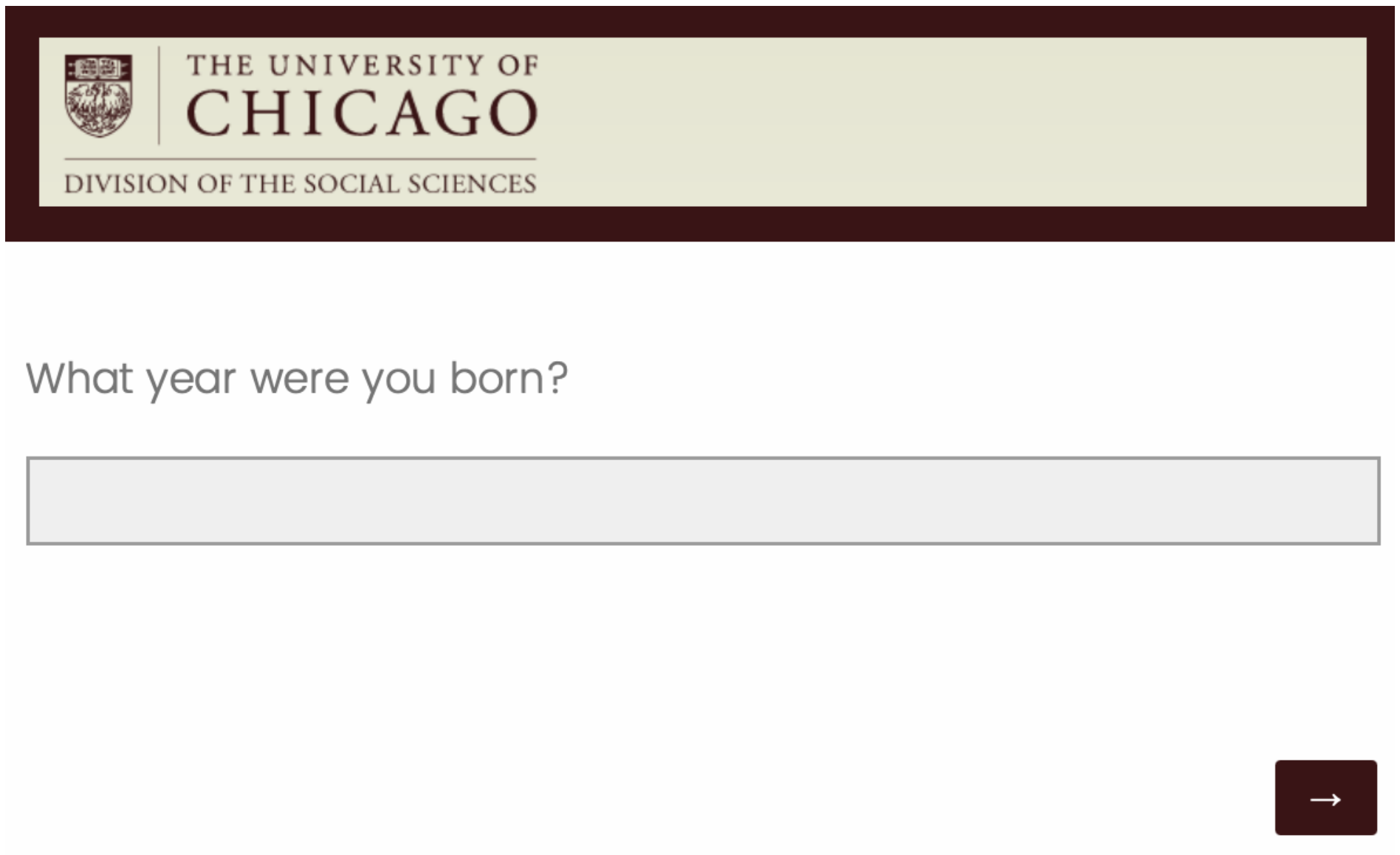


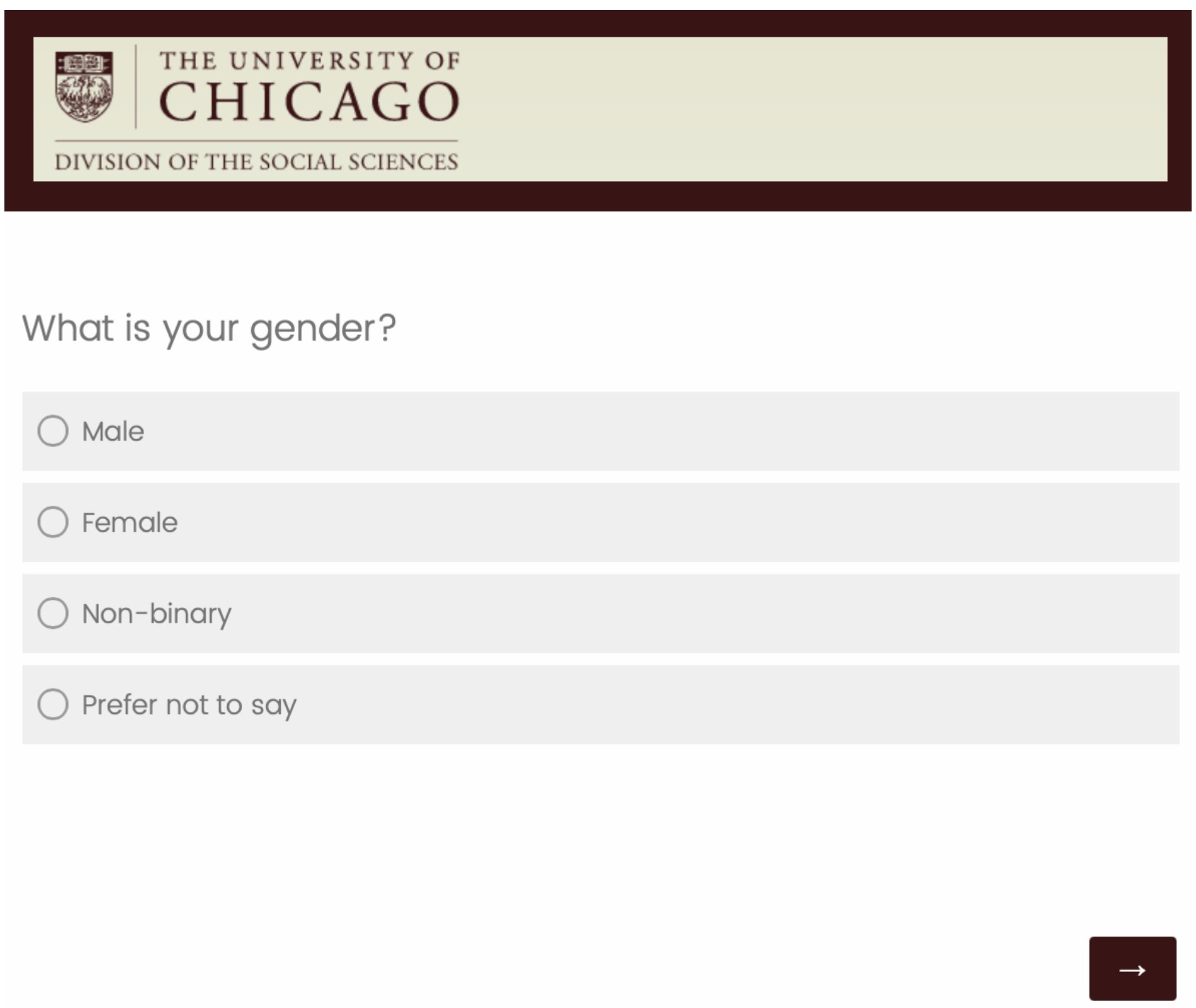

THE UNIVERSITY OF CHICAGO

DIVISION OF THE SOCIAL SCIENCES

What is your race/ethnicity? (Select all that apply)

- ☐ Hispanic or Latino
- ☐ White
- ☐ Black or African American
- ☐ Asian
- ☐ Native American or Alaska Native
- ☐ Native Hawaiian or Pacific Islander
- ☐ Other

→

THE UNIVERSITY OF CHICAGO

DIVISION OF THE SOCIAL SCIENCES

How many children do you have in each age group?

| | 0 | 1 | 2 | 3 or more |
|---|---|---|---|---|
| 0-4 years | ○ | ○ | ○ | ○ |
| 5-12 years | ○ | ○ | ○ | ○ |
| 13-18 years | ○ | ○ | ○ | ○ |

→

THE UNIVERSITY OF CHICAGO
DIVISION OF THE SOCIAL SCIENCES

What is the gender of your children? (Select all that apply)

- ☐ Male
- ☐ Female
- ☐ Non-binary
- ☐ Prefer not to say
- ☐ I do not have children

→

THE UNIVERSITY OF CHICAGO

DIVISION OF THE SOCIAL SCIENCES

Recent research has shown that using advanced AI models helps improve performance on writing and language tasks **while it is being used**.

On the other hand, a field experiment with high school students found that access to GPT for learning led to a **nearly 20% decline** in quantitative reasoning scores when students were later tested without AI.

Why did scores decline?

- **Over-reliance on AI:** Students used GPT to generate ideas rather than engaging in problem-solving themselves.
- **Weaker mental effort:** They became less practiced in structuring arguments and reasoning independently.
- **Reduced cognitive engagement:** AI-assisted learning replaced the effort required to develop analytical thinking.

→

THE UNIVERSITY OF CHICAGO

DIVISION OF THE SOCIAL SCIENCES

We will run a survey about AI use among teenagers. The survey will allow us to estimate the **share of teenagers in your country who use AI tools**. The next question will be hypothetical, based on what the outcome of that survey may be.

→

THE UNIVERSITY OF CHICAGO
DIVISION OF THE SOCIAL SCIENCES

Imagine the scenario that **80% of teenagers** use AI tools.

Would you prefer if no student was allowed to use AI tools and instead learned the way they used to before AI tools were introduced?

- ◯ Yes
- ◯ No

→

THE UNIVERSITY OF CHICAGO
DIVISION OF THE SOCIAL SCIENCES

Imagine the scenario that **20% of teenagers** use AI tools.

Would you prefer if no student was allowed to use AI tools and instead learned the way they used to before AI tools were introduced?

- ◯ Yes
- ◯ No

→

THE UNIVERSITY OF CHICAGO
DIVISION OF THE SOCIAL SCIENCES

Do you agree with the following statement:
“If my child does not use AI-based learning or tutoring tools, they will be at a disadvantage compared with their peers.”

- ◯ Strongly disagree
- ◯ Somewhat disagree
- ◯ Neither agree nor disagree
- ◯ Somewhat agree
- ◯ Strongly agree

→

THE UNIVERSITY OF CHICAGO

DIVISION OF THE SOCIAL SCIENCES

Do you agree with the following statement:
"My child will fall behind if they do not use AI."

- ◯ Strongly disagree
- ◯ Somewhat disagree
- ◯ Neither agree nor disagree
- ◯ Somewhat agree
- ◯ Strongly agree

→

THE UNIVERSITY OF CHICAGO

DIVISION OF THE SOCIAL SCIENCES

If no other children in your child's class used AI tools, how likely would you be to let your child use them?

- ○ Extremely unlikely
- ○ Somewhat unlikely
- ○ Neither likely nor unlikely
- ○ Somewhat likely
- ○ Extremely likely

→

### D. Experiment 4.

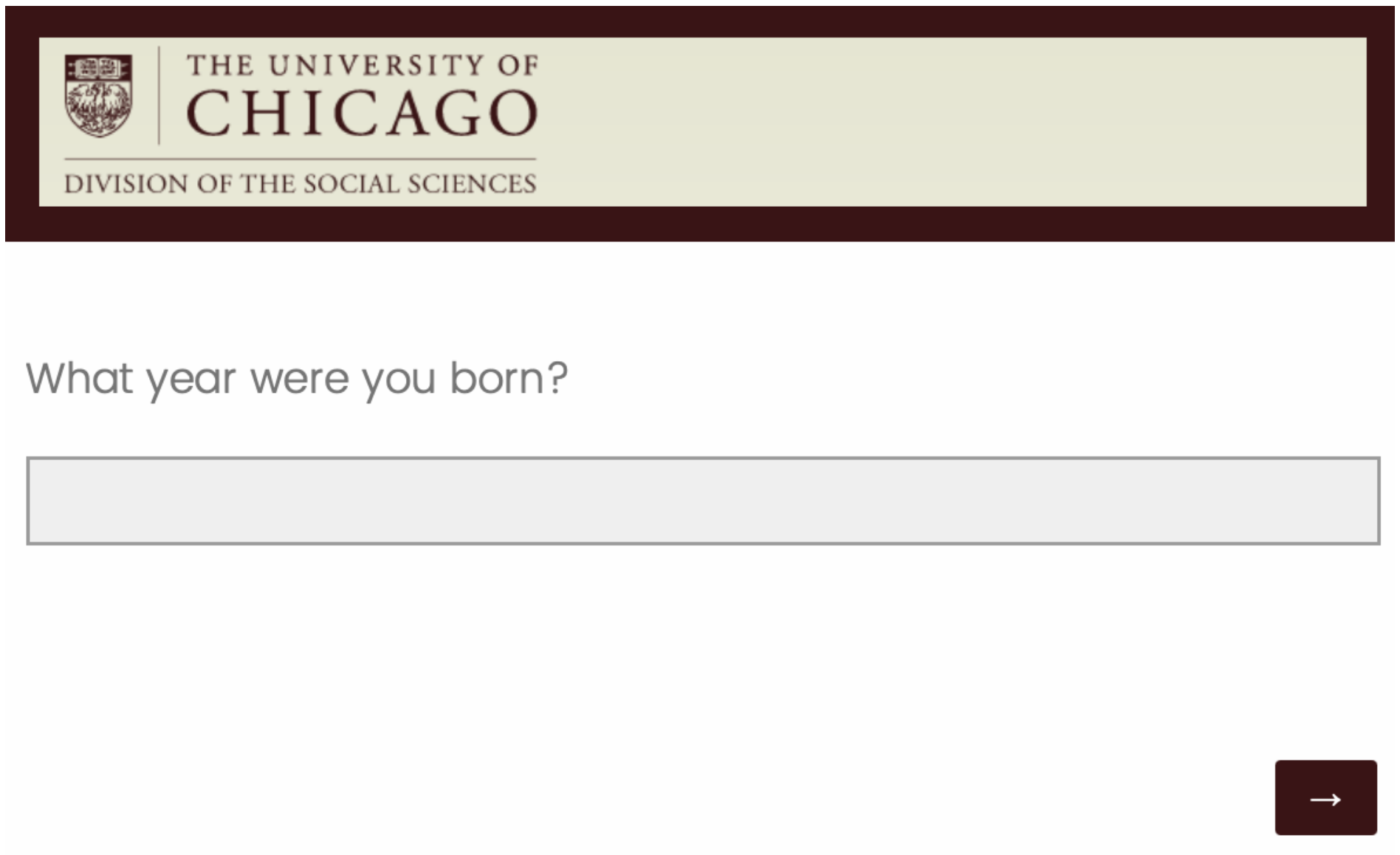
THE UNIVERSITY OF CHICAGO
DIVISION OF THE SOCIAL SCIENCES

What year were you born?

→

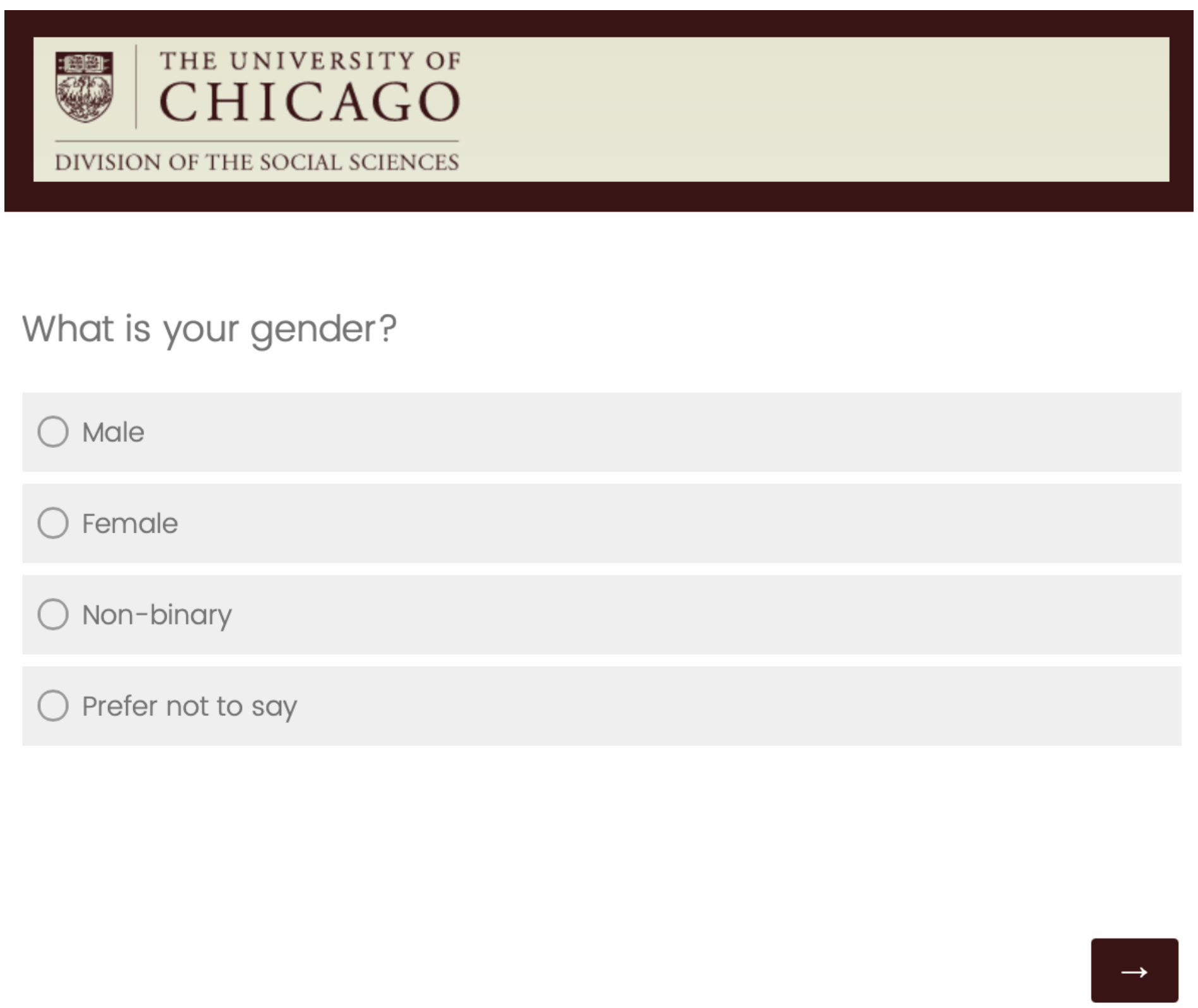
THE UNIVERSITY OF CHICAGO
DIVISION OF THE SOCIAL SCIENCES

What is your gender?

- Male
- Female
- Non-binary
- Prefer not to say

→

THE UNIVERSITY OF CHICAGO
DIVISION OF THE SOCIAL SCIENCES

What is your race/ethnicity? (Select all that apply)

- ☐ Hispanic or Latino
- ☐ White
- ☐ Black or African American
- ☐ Asian
- ☐ Native American or Alaska Native
- ☐ Native Hawaiian or Pacific Islander
- ☐ Other

→

THE UNIVERSITY OF CHICAGO
DIVISION OF THE SOCIAL SCIENCES

How many children do you have in each age group?

| | 0 | 1 | 2 | 3 or more |
|---|---|---|---|---|
| 0-4 years | ○ | ○ | ○ | ○ |
| 5-12 years | ○ | ○ | ○ | ○ |
| 13-18 years | ○ | ○ | ○ | ○ |

→

THE UNIVERSITY OF CHICAGO
DIVISION OF THE SOCIAL SCIENCES

What is the gender of your children? (Select all that apply)

- ☐ Male
- ☐ Female
- ☐ Non-binary
- ☐ Prefer not to say
- ☐ I do not have children

→

THE UNIVERSITY OF CHICAGO

DIVISION OF THE SOCIAL SCIENCES

Recent research has shown that using advanced AI models helps improve performance on writing and language tasks **while it is being used**.

On the other hand, a field experiment with high school students found that access to GPT for learning led to a **nearly 20% decline** in quantitative reasoning scores when students were later tested without AI.

Why did scores decline?

- **Over-reliance on AI:** Students used GPT to generate ideas rather than engaging in problem-solving themselves.
- **Weaker mental effort:** They became less practiced in structuring arguments and reasoning independently.
- **Reduced cognitive engagement:** AI-assisted learning replaced the effort required to develop analytical thinking.

→

THE UNIVERSITY OF CHICAGO
DIVISION OF THE SOCIAL SCIENCES

We recently ran a survey on AI use. Among a sample of these respondents in your state, we found that approximately **80% of teenagers** use AI tools.

→

THE UNIVERSITY OF CHICAGO
DIVISION OF THE SOCIAL SCIENCES

We recently ran a survey on AI use. Among a sample of these respondents in your state, we found that approximately **40% of teenagers** use AI tools.

→

THE UNIVERSITY OF CHICAGO
DIVISION OF THE SOCIAL SCIENCES

Would you prefer if no student was allowed to use AI tools and instead learned the way they used to before AI tools were introduced?

- Yes
- No

→

THE UNIVERSITY OF CHICAGO
DIVISION OF THE SOCIAL SCIENCES

We are also interested in your perceptions of AI products.

Please rate the **overall quality** of **Claude Pro** for your teenager's use.

Low quality — High quality

0 1 2 3 4 5 6 7 8 9 10

Claude Pro

→

THE UNIVERSITY OF CHICAGO
DIVISION OF THE SOCIAL SCIENCES

How important do you think the following skills will be to the success of your teenager over the next 10 years?

| | Not at all important | Slightly important | Moderately important | Very important | Extremely important |
|---|---|---|---|---|---|
| Critical thinking | ○ | ○ | ○ | ○ | ○ |
| Writing and communication | ○ | ○ | ○ | ○ | ○ |
| Creativity | ○ | ○ | ○ | ○ | ○ |
| Quantitative reasoning | ○ | ○ | ○ | ○ | ○ |
| Teamwork | ○ | ○ | ○ | ○ | ○ |
| Self discipline | ○ | ○ | ○ | ○ | ○ |

→

THE UNIVERSITY OF CHICAGO
DIVISION OF THE SOCIAL SCIENCES

How important do you think AI literacy is to the long term success of your teenager?

Not important — Very important

0 1 2 3 4 5 6 7 8 9 10

Importance

→

THE UNIVERSITY OF CHICAGO
DIVISION OF THE SOCIAL SCIENCES

Do you agree with the following statement:
“If my teenager does not use AI-based learning or tutoring tools, they will be at a disadvantage compared with their peers.”

- ◯ Strongly disagree
- ◯ Somewhat disagree
- ◯ Neither agree nor disagree
- ◯ Somewhat agree
- ◯ Strongly agree

→

THE UNIVERSITY OF CHICAGO
DIVISION OF THE SOCIAL SCIENCES

Do you agree with the following statement:
"My teenager will fall behind if they do not use AI."

- ◯ Strongly disagree
- ◯ Somewhat disagree
- ◯ Neither agree nor disagree
- ◯ Somewhat agree
- ◯ Strongly agree

→

THE UNIVERSITY OF CHICAGO

DIVISION OF THE SOCIAL SCIENCES

If no other student in your teenager's class used AI tools, how likely would you be to let your teenager use them?

- Extremely unlikely
- Somewhat unlikely
- Neither likely nor unlikely
- Somewhat likely
- Extremely likely

→

THE UNIVERSITY OF CHICAGO

DIVISION OF THE SOCIAL SCIENCES

What percentage of teenagers do you think use AI tools in your state?

0 10 20 30 40 50 60 70 80 90 100

%

→

THE UNIVERSITY OF CHICAGO

DIVISION OF THE SOCIAL SCIENCES

Note that in our full sample of respondents across the US, we found that over 90% of teenagers use AI tools.

→

**E. Experiment 5.** The survey instructions closely follow experiment 1. Of note, there is a new information screen for treatment, and adjustments to other questions, all shown below.

Which of the following AI tools do **your teenagers** use? (Select all that apply)

- ☐ ChatGPT
- ☐ Claude
- ☐ Gemini
- ☐ Llama
- ☐ Math solvers (Photomath, etc.)
- ☐ Tutoring tools (Khanmigo, etc.)
- ☐ Writing tools (Grammarly, etc.)
- ☐ Others
- ☐ Not sure
- ☐ None

How competitive would you describe the academic environment at your teenager's school?

- ○ Not at all competitive
- ○ Slightly competitive
- ○ Moderately competitive
- ○ Very competitive
- ○ Extremely competitive

Recent research has shown that using advanced AI models helps improve performance on writing and language tasks **while it is being used.**

On the other hand, a field experiment with high school students found that access to GPT for learning led to a **nearly 20% decline** in quantitative reasoning scores when students were later tested without AI.

Why did scores decline?

- **Over-reliance on AI:** Students used AI to generate ideas rather than engaging in problem-solving themselves.
- **Weaker mental effort:** They became less practiced in structuring arguments and reasoning independently.
- **Reduced cognitive engagement:** AI-assisted learning replaced the effort required to develop analytical thinking.

As well, a recent Brookings Institution study reviewing hundreds of research articles warns that AI in education can "undermine children's foundational development" and that “at this point in its trajectory, the risks of utilizing AI in education overshadow its benefits”. Researchers caution that the very skills students need to succeed in the workforce could be affected and limit their future professional success.

**Experiment 6.** The survey instructions follow the same logic as experiments 1, 2, and 5, but focus on two different AI tools (as opposed to Claude Pro in previous experiments). New product descriptions and ban questions are shown below.

In this part of the survey you will have the chance to win a one-year subscription to Khanmigo, Khan Academy's AI tutor, currently valued at $44. Unlike regular AI tools that provide direct answers, Khanmigo guides your teenager through problems step-by-step —asking questions, giving hints, and helping them work through solutions themselves. The subscription is intended for your teenager's use to help with homework and learning.

In this part of the survey you will have the chance to win a one-year subscription to Photomath Plus, a math-solving app, currently valued at $70. Photomath lets your teenager take a photo of a math problem and instantly see the answer along with step-by-step explanations of how to solve it. The subscription is intended for your teenager's use to help with math homework and learning.

Would you prefer if no student was allowed to use AI tutoring tools, like Khanmigo, and instead learned the way they used to before AI tools were introduced?

- Yes
- No

Would you prefer if no student was allowed to use AI math-solving tools, like Photomath, and instead learned the way they did before AI tools were introduced?

- Yes
- No

**Experiment 7.** The survey instructions follow the same logic as experiments 1 and 2, but only ask each participant about one scenario. The scenarios are shown below.

> We are conducting this survey with a large population of parents with teenagers. From this survey, we will be able to estimate what percentage of teenagers use AI tools for things like schoolwork and research.
>
> We would like you to consider your decision to get Claude Pro for your teenager **in a scenario** that depends on how many other teenagers also use similar technology. For example, we could ask you how much you would be willing to pay for Claude Pro for your teenager in the case that 40% of other teenagers use and have access to AI tools.
>
> Once we have collected all survey responses, we will determine if your scenario comes to the actual percentage of teenagers using AI in our sample. If your scenario comes close (within 10 percentage points) and if you are randomly chosen to receive a reward, then whether you win the Claude Pro subscription or the bonus will be determined by your answer.

You will now enter how much you would be willing to pay (by forgoing your bonus) for a three-month subscription to Claude Pro for your teenager under your assigned scenario. The scenario will include information on how many other teenagers in our sample - of the same age and from the same country as your own - use AI.

**Scenario:**

**80%** of teenagers use AI.

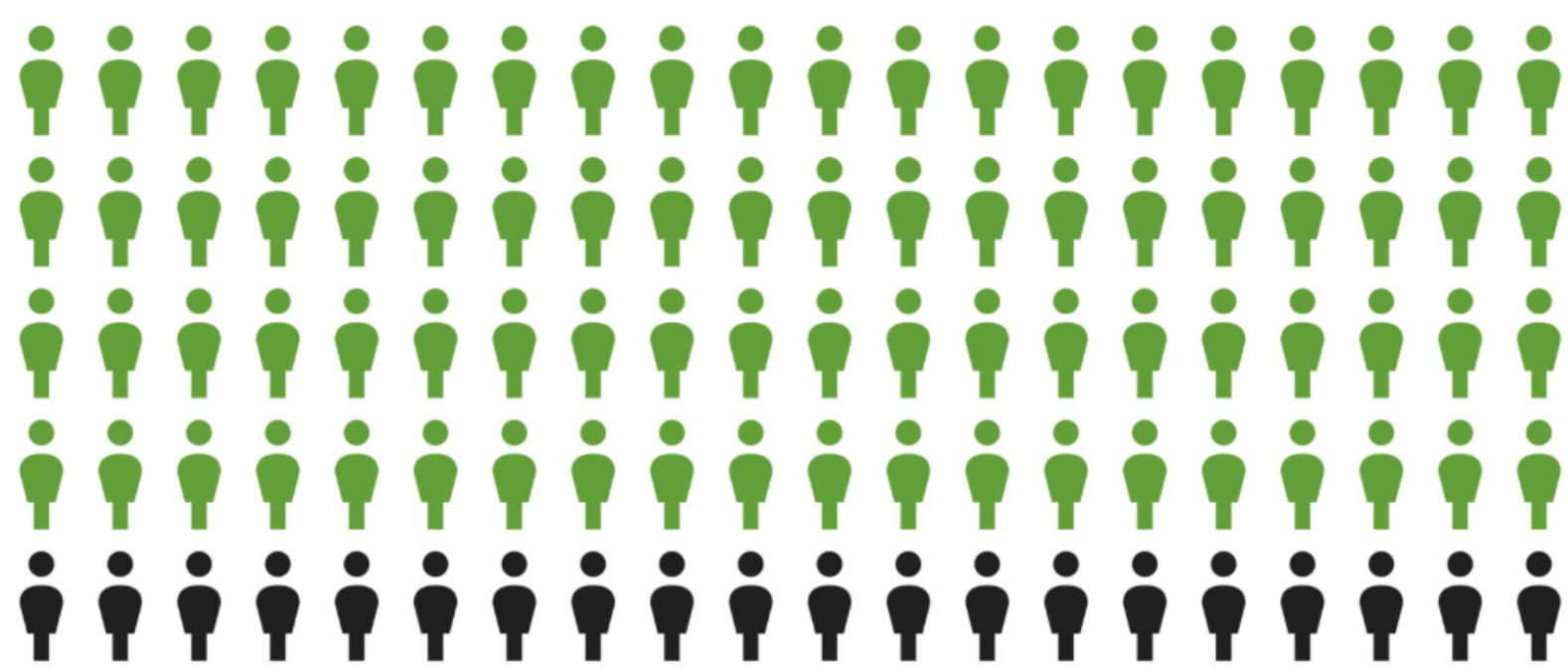

If **80% of teenagers** use AI, how much would you pay for a Claude Pro subscription for your teenager?

You will now enter how much you would be willing to pay (by forgoing your bonus) for a three-month subscription to Claude Pro for your teenager under your assigned scenario. The scenario will include information on how many other teenagers in our sample - of the same age and from the same country as your own - use AI.

**Scenario:**

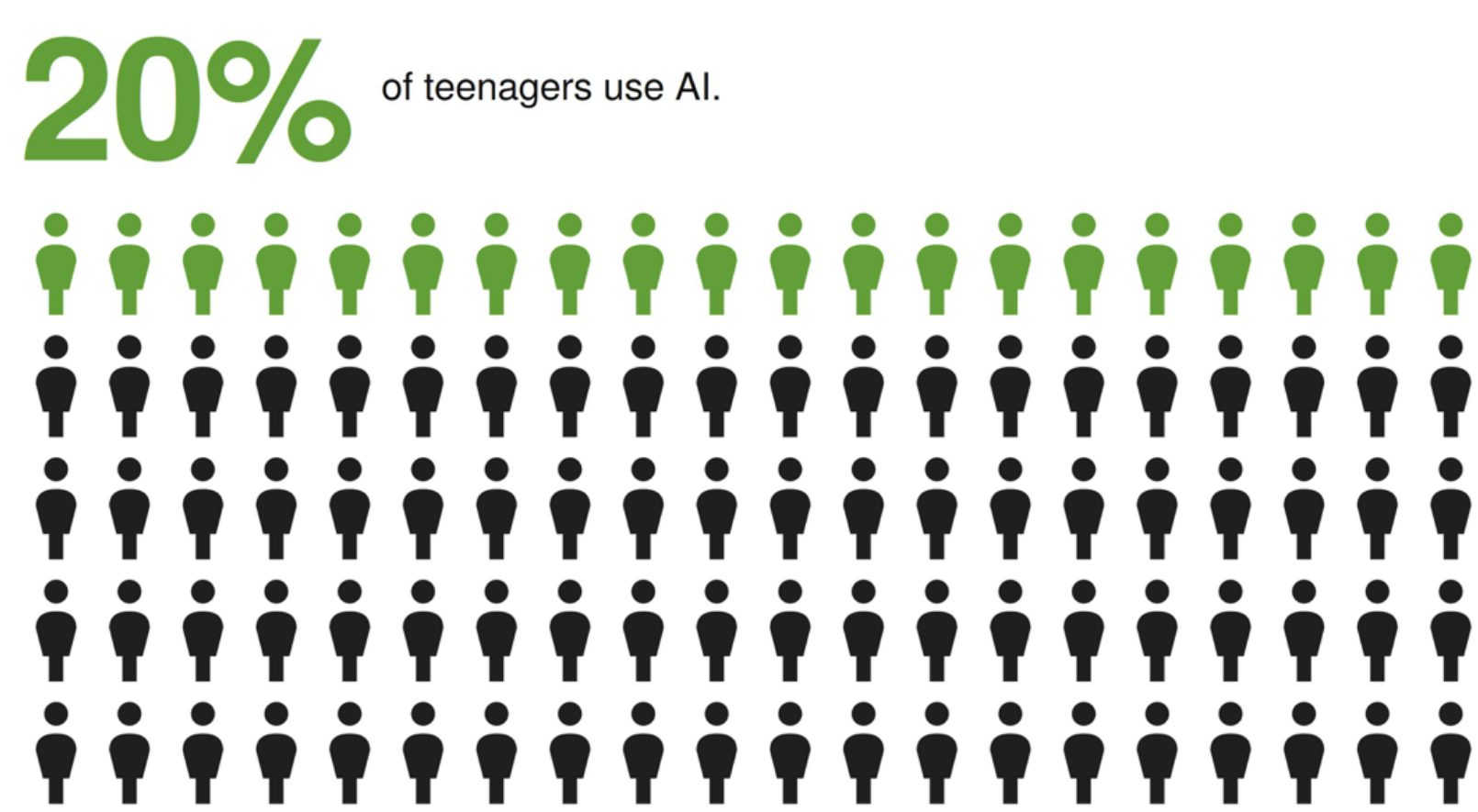


If **20% of teenagers** use AI, how much would you pay for a Claude Pro subscription for your teenager?

**Experiment 8.** The survey instructions follow the same scenarios as experiment 7 but also cross-randomizes the information treatment from experiment 1.

## References


1. H Bastani, et al., Generative AI without guardrails can harm learning: evidence from high school mathematics. *Proc. Natl. Acad. Sci.* **122**, e2422633122 (2025).
2. HB Essel, D Vlachopoulos, AB Essuman, JO Amankwa, ChatGPT effects on cognitive skills of undergraduate students: receiving instant responses from AI-based conversational large language models (llms). *Comput. Educ. Artif. Intell.* **6**, 100198 (2024).
3. GM Becker, MH DeGroot, J Marschak, Measuring utility by a single-response sequential method. *Behav. Sci.* **9**, 226–232 (1964).
4. P Andre, T Boneva, F Chopra, A Falk, Misperceived social norms and willingness to act against climate change. *Rev. Econ. Stat.* (2024).
5. I Haaland, C Roth, J Wohlfart, Designing information provision experiments. *J. Econ. Lit.* **61**, 3–40 (2023).
6. S Stantcheva, How to run surveys: A guide to creating your own identifying variation and revealing the invisible. *Annu. Rev. Econ.* **15**, 205–234 (2023).
